# Light‐Induced Entropy for Secure Vision

**DOI:** 10.1002/adma.202516947

**Published:** 2026-01-29

**Authors:** Juhyung Seo, Seungme Kang, Chaehyun Kim, Taehyun Park, Youngwoo Yoo, Yeong Kwon Kim, Wonjun Shin, Byung Chul Jang, Young‐Joon Kim, Hocheon Yoo

**Affiliations:** ^1^ Department of Electronic Engineering Hanyang University Seoul Republic of Korea; ^2^ Department of Semiconductor Engineering Gachon University Seongnam Gyeonggi‐do Republic of Korea; ^3^ School of Electronic and Electrical Engineering Kyungpook National University Daegu Republic of Korea; ^4^ Department of Electrical and Computer Engineering Sungkyunkwan University (SKKU) Suwon Republic of Korea; ^5^ Department of Electronic Engineering Gachon University Seongnam Gyeonggi‐do Republic of Korea; ^6^ Department of Artificial Intelligence Semiconductor Engineering Hanyang University Seoul Republic of Korea

**Keywords:** image security, photodetectors, photospike, stochastic electronics, true random number generator

## Abstract

We present a photospike‐based true random number generator (PS‐TRNG) that exploits the intrinsic randomness from light–matter interaction and stochastic charge trapping. By integrating copper vanadate (CuV_2_O_6_) nanostructures with a tin dioxide quantum dot (SnO_2_ QD) layer, the device induces probabilistic trapping–de‐trapping dynamics, producing random photospike currents under optical pulse trains. The spike currents show high entropy and enable multi‐level random number generation beyond binary, providing ternary outputs with near‐ideal statistics (33.30% uniformity, 33.28% inter‐Hamming distance) and full success in all 15 NIST tests. We further develop an image authenticity verification system by integrating the PS‐TRNG with a mobile platform and custom‐designed circuit board, enabling hardware‐based detection of unauthorized image modifications. The random numbers are embedded as a hidden layer within the image data without degrading visual quality, enabling detection of unauthorized modifications. The system can successfully identify image modifications, even those involving highly sophisticated manipulations generated by artificial intelligence (AI)‐based image editing tools. The device maintains stable operation over 2 million cycles and remains reliable even after more than 460 days, demonstrating its long‐term stability.

## Graphical abstract

This work realized a ternary true random number generator by exploiting stochastic traps emerging within multiple junction interfaces, and quantitatively validated the generation of high‐quality random numbers. Furthermore, it successfully demonstrated diverse applications, including AI‐resilient image security, thereby providing a valuable guide for future studies on random number generators.

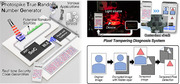

## 1 Introduction

The rapid advancement of the information society has led to an exponential increase in the amount of data generated and processed by humanity. The total volume of global data, which was approximately 2 zettabytes (ZB) in 2010, is projected to reach 175 ZB by 2025, which is an 87‐fold increase over just 15 years [1, 2]. Many conventional services have either evolved into intelligent, data‐driven systems that offer highly personalized user experiences or have become obsolete. While these technologies bring great convenience and personalization, they also heighten the risk of data misuse. The biggest concern is the leakage or unauthorized use of personal information, which can lead to serious cybercrimes [3, 4]. Throughout history, various encryption schemes have been developed to secure information transmission and usage, ranging from early manual ciphers to modern cryptographic systems based on pseudo‐random number generators (PRNGs) grounded in advanced mathematics [5]. However, as the name suggests, pseudo‐random number generators produce sequences that are not truly random, but rather deterministically derived from an initial seed using mathematical algorithms. Thus, such systems suffer from inherent vulnerabilities to security breaches, especially when the seed is exposed or the underlying algorithm is compromised via reverse engineering.

As an alternative approach to overcome the limitations of existing methods, true random number generators (TRNGs) are emerging as alternative technologies that exploit diverse sources of physical entropy [6, 7, 8, 9, 10]. One notable approach is demonstrated by Cloudflare, a global cybersecurity company in San Francisco, which constructed a ‘wall of entropy’ composed of dozens of lava lamps. The unpredictable motion of the glowing fluid wax inside the lamps, captured by cameras, has been used to generate true random numbers for large‐scale cryptographic systems, serving as a practical demonstration of physical entropy [11]. Another approach involves quantum random number generators (QRNGs), which utilize the quantum nature of light to produce random numbers based on the probabilistic arrival of single photons [12, 13, 14]. While these platforms have successfully demonstrated high‐quality entropy, their implementations often involve system‐level optical components or external modules that may not be readily adaptable to compact, device‐level integration.

As a physical stimulus that is both abundant and readily accessible, light offers inherent unpredictability when interacting with matter, making it a promising candidate for entropy generation [15, 16]. These brief pulses of light, through their interaction with matter, provide a robust physical source of entropy for generating true random numbers essential to cryptographic security [17, 18, 19]. Such a physical source of randomness offers a promising pathway for securing data streams. When implemented at the hardware level, light‐driven entropy sources provide a reliable foundation for cryptographic functions. Rather than relying on algorithmic approaches, which are inherently deterministic and subject to reverse engineering, physically generated random numbers are non‐reproducible and device‐specific [20]. These characteristics enable secure key generation, challenge‐response authentication, data obfuscation, and physically unclonable functions (PUFs) within compact and scalable hardware architectures [21, 22].

Meanwhile, multi‐valued systems are attracting attention for their benefits in data compression and security. For example, while a four‐digit binary PIN has a 6.25% chance of being guessed, a ternary PIN reduces this to only 1.2%, showing how higher data density greatly strengthens security. In this context, exploiting light as an entropy source not only induces diverse photo‐responsive physical changes but also enables multi‐valued random number generation, where a single optical source can facilitate parallel entropy harvesting across multiple generators. Such an approach offers an approach toward high‐throughput randomness generation, thereby bridging physical entropy with practical implementations in secure hardware.

Based on light‐driven entropy that induces photo‐responsive variations and parallel multi‐valued randomness, we present a photospike‐based true random number generator (PS‐TRNG) that works with image sensors, can be integrated into circuits, and produces multi‐valued random logic in real time. The system utilizes the stochastic trapping and detrapping of photo‐excited carriers in oxide heterostructures to produce spike‐like photocurrent signals. These spike‐like photocurrent signals are amplified and sampled through a PRNG‐guided readout process, which converts their stochastic intensity variations into discrete multi‐valued logic states. Through this process, the intrinsic fluctuations generated by trap–detrap dynamics are effectively transferred into the electrical domain, producing ternary random numbers with enhanced entropy and stable randomness. The generated random output exhibited an ideal uniformity of 33.30% and an inter‐Hamming distance (Inter‐HD) of 33.28%, while successfully passing all 15 items of the National Institute of Standards and Technology (NIST) randomness test suite [23], outperforming the binary PS‐TRNG input used in the sampling process. We implemented a real‐time pixel tampering diagnosis (PTD) system that embeds a physically unclonable random‐number‐based hidden layer within image data. This system accurately detected artificial intelligence (AI)‐generated forgeries that were visually indistinguishable from authentic images [24, 25, 26, 27, 28]. Further validation revealed that the entropy originated intrinsically from device‐level dynamics, not the light source, and the system maintained reliable performance over 460 days and millions of data points.

## 2 Results and Discussion

**FIGURE 1:**
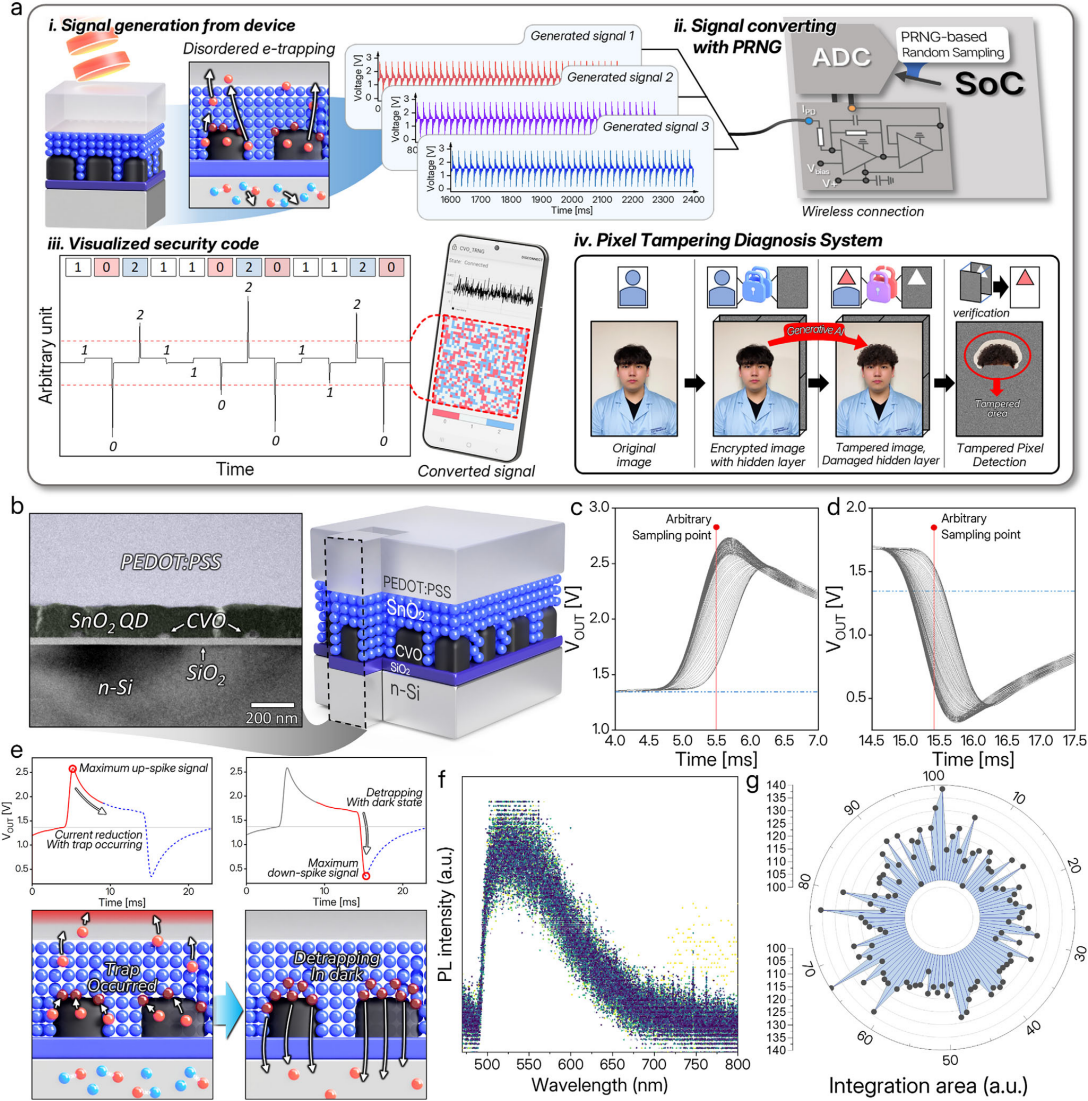
Signal output system of the PS‐TRNG device and triggering mechanism of spike peaks. (a) Schematic of a ternary random number generation system based on a PS‐TRNG device and its application to the PTD system. (b) Schematic of the fabricated PS‐TRNG device and TEM cross‐sectional images. Distribution of photocurrent changes and spike peaks when light is switched to (c) on and (d) off. (e) Mechanism of photocurrent variation based on charge trapping in PS‐TRNG devices. (f) PL analysis of the SnO_2_ QDs/CVO NDs heterostructure and (g) integrated area distribution from 100 repetitive PL spectrum measurements. Images of individuals are used with consent.

As illustrated in Figure 1a, a circuit system was implemented to generate ternary‐valued true random numbers in real‐time. In heterojunction devices, the unpredictable potential random source generated with red light pulses was sampled and amplified by a custom‐built System‐on‐Chip (SoC). The sampled data was transmitted wirelessly to a commercial mobile device, where it was classified based on a predefined threshold, to successfully demonstrate ternary random numbers and visualize the generated security code in real‐time. This process was utilized to encrypt an image with a hidden layer, and the encrypted code successfully detected tampered pixels in the sensing image. The origin of the physically unclonable potential random source embedded in such devices arises from the complex charge trapping and detrapping phenomena between two oxide semiconductor nanostructures. This PS‐TRNG device is characterized by a structure comprising a bottom *n*‐Si, a following copper vanadate (CuV_2_O_6_, CVO) nano dots (NDs), and top tin dioxide quantum dots (SnO_2_ QDs), with the top electrode being poly(3,4‐ethylenedioxythiophene): polystyrene sulfonate (PEDOT:PSS) [29, 30], as shown in the high‐resolution transmission electron microscope image (TEM) and corresponding schematic illustration in Figure 1b. The detailed crystallographic and morphological characteristics of the fabricated layers are described in Note S1 and Figures S1–S3.

To implement true randomness with a high level of entropy, we introduced light‐driven stochastic trapping and de‐trapping, with device‐level variability achieved through defect engineering [31]. Under pulsed light excitation, the PS‐TRNG device exhibited spike‐like photocurrent peaks, consisting of positive transients during illumination and negative ones following light‐off conditions (Figure S4). As shown in Figure 1c,d, both the peak intensity and rise time varied significantly across cycles, reflecting the inherently random nature of the underlying carrier dynamics. This unpredictability in the current response allows for the generation of random outputs by sampling points along with the positive and negative transients. In this configuration, PRNG signals were applied to the readout circuit as external sampling triggers to eliminate periodicity arising from light‐induced currents, while ensuring that the extracted random outputs were governed by the stochastic trapping and de‐trapping dynamics of the device. Consequently, the extracted data reflected the stochastic trapping‐de‐trapping behavior of the device rather than the periodic characteristics of optical excitation.

These spike peaks, representing transient photo response, have been reported in previous studies, where the main factor contributing to the photocurrent spike was suggested to be trap‐assisted carrier recombination [32, 33]. In the proposed PS‐TRNG, under illumination at defined wavelengths, photocarriers are generated in *n*‐Si region owing to the ultra‐wide bandgap of top SnO_2_ QDs and separated by the presence of built‐in electric fields between *n*‐Si and SnO_2_ QDs (described in detail in Note S2 and Figures S5–S7). As shown in Figure 1e and Figure S8a,b, photoexcited electrons are transported from the *n*‐Si toward the PEDOT:PSS electrode, during which they become trapped at surface defect sites along randomly distributed pathways through the SnO_2_QDs. This process generates transient spike currents characterized by a rapid rise and decay in photocurrent. Once the available trap states are saturated, the photocurrent stabilizes at a steady level, as shown in Figure S8c. Upon termination of the optical excitation, the built‐in electric field reverses due to the accumulated trapped charges, resulting in the release of detrapped electrons and a subsequent reverse current. This leads to negative spike photocurrents associated with the light‐off response. To investigate the chaotical charge trapping and de‐trapping dynamics in the proposed device scheme, we performed the photoluminescence (PL) analysis. As shown in Figure 1f, a randomly varied distribution of defect‐related PL peaks was observed under repeated excitation and emission measurement of 100 times, which suggests the trapped and detrapped charge‐assisted random movements of carriers through the input excitation light pulses, as shown in Figure S9. Integrating area distributions of each PL spectrum in Figure 1g further quantitatively represents the chaotical change in charge trap dynamics. Moreover, we investigated photocarrier trapping/de‐trapping dynamics by performing low‐frequency noise (LFN) measurements, with a detailed analysis in Note S3 and Figures S10 and S11.

**FIGURE 2:**
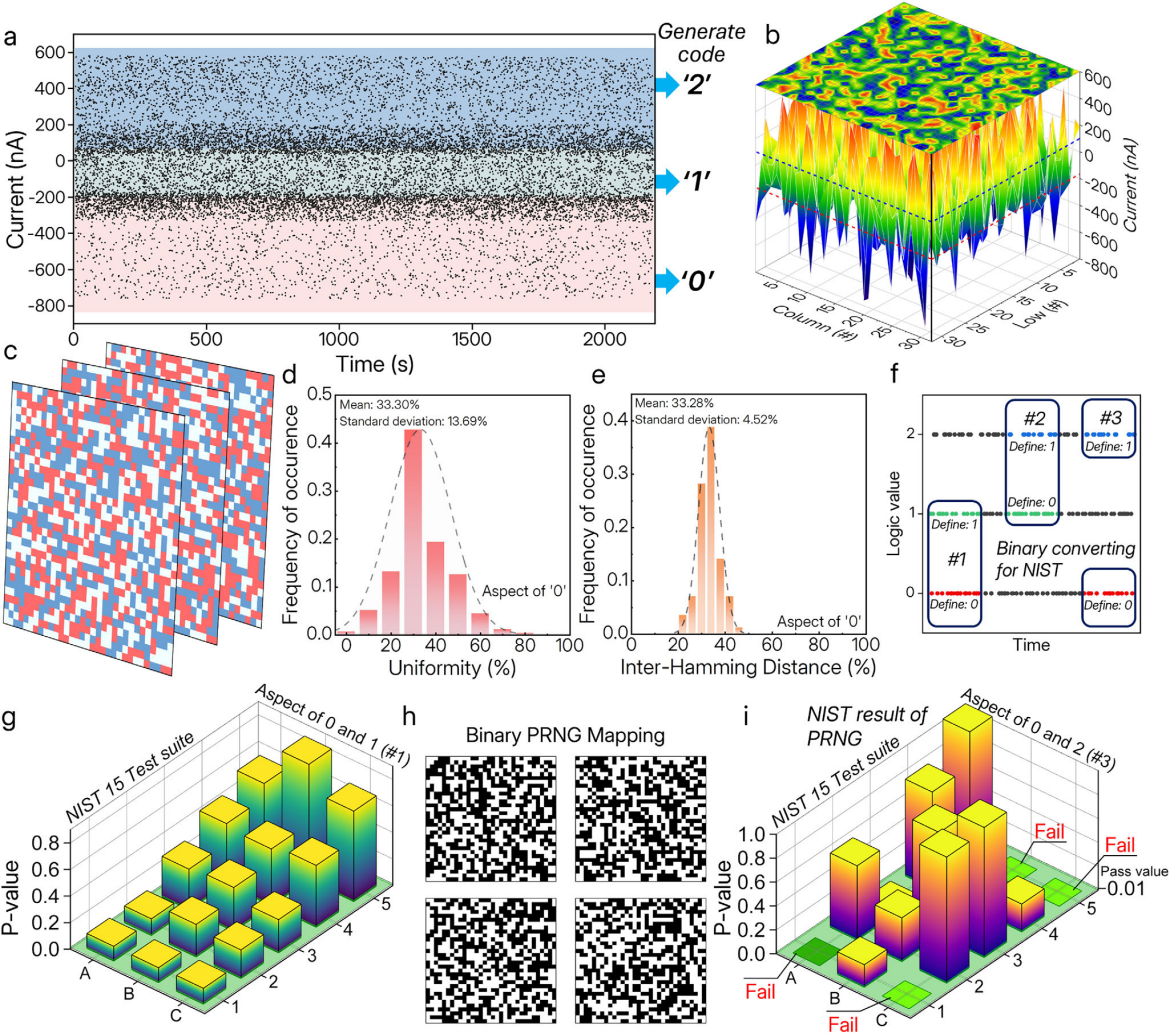
Randomness evaluation and NIST testing of the output ternary random number signal. (a) Ternary trit division of the photocurrent distribution generated by the PS‐TRNG device and (b) 3D contour mapping of the data. (c) The obtained ternary bit‐mapping image. Evaluation of randomness via the (d) uniformity and (e) inter‐HD metric for each of the ‘0’ trit. (f) Explanation of NIST verification method for ternary random numbers and (g) Results of NIST statistical tests for the proposed ternary random numbers (Aspect of 0 and 1). (h) Visualization of the extracted bitmaps generated by the PRNG source. (i) NIST statistical test results indicating failure to meet randomness criteria, underscoring the limitations of the PRNG‐based approach.

Building on the unpredictable and unclonable behavior of the heterojunction device, we implemented the PS‐TRNG, as shown in Figure 2. The device operates under zero external bias, utilizing only the photocurrent generated by light excitation. Photocurrents generated under 660 nm pulsed red light (0.53 mW·cm^−2^, 50 Hz) were digitized into ternary values (‘0’, ‘1’, ‘2’) using predefined thresholds: below –187.5 nA (‘0’), above 91.8 nA (‘2’), and intermediate values as ‘1’. To validate the true randomness for security applications, we analyzed the statistical characteristics of 10 240 ternary outputs using uniformity, inter‐HD. Uniformity quantifies the balance in the distribution among the possible logic states. For a ternary system, ideal uniformity corresponds to an equal probability (33.3%) for each state, indicating unbiased output and consistent randomness. Inter‐HD quantifies how similar or dissimilar each row is to other rows within the matrix. As with uniformity, the ideal value is 33.33%, which reflects the level of uniqueness and unpredictability required for hardware‐level security. The logic states appeared with nearly equal probability—33.30% for ‘0’, 33.32% for ‘1’, and 33.37% for ‘2’ (Figure 2d; Figure S17a,b). Inter‐HD analysis confirmed uniqueness across the dataset, yielding 33.28% for ‘0’, 33.39% for ‘1’, and 33.32% for ‘2’ (Figure 2e; Figure S17c,d), which closely match the ideal value for ternary random generation. As shown in Figure S17e, the entropy value was measured to be 1.552, which is close to the theoretical maximum of 1.585, confirming that the generated ternary outputs exhibit near‐ideal randomness. It is noted that since no standard test metrics exist for ternary physical random numbers, we devised an evaluation method here to assess their quality.

Figure S13 presents the signal‐to‐noise ratio (SNR) analysis of the PS‐TRNG output to verify that the photocurrent spikes used for ternary conversion exhibit sufficient signal quality. When the PS‐TRNG is optically switched, transient spiking peaks appear under both light‐on and light‐off conditions, corresponding to positive and negative photocurrents, respectively. Although the peak intensity and rise/decay time of each spike vary across cycles owing to stochastic trapping and de‐trapping dynamics, the measured SNR remains sufficiently high. The SNR values derived from the maximum photospike currents exceed 77 for the light‐on state and 95 for the light‐off state, confirming that the PS‐TRNG maintains high‐quality photocurrent signals suitable for reliable ternary random number generation. In addition, Figure S14 compares and presents security patterns of different scales to verify stability against variations in mapping size. The security pattern represents a visualized matrix of the extracted ternary random stream, in which each random value is sequentially arranged in rows and columns to form a 32 × 32 map. Each element in the map corresponds to the output state (‘0’, ‘1’, or ‘2’) and is displayed in three different color tones for visual clarity (blue: ‘0’, light blue: ‘1’, red: ‘2’). To confirm that this mapping process does not affect the statistical characteristics of randomness, we analyzed the uniformity and inter‐HD of maps with different matrix sizes (16 × 16, 32 × 32, and 64 × 64). As shown in Figures S15 and S16, all cases exhibited nearly identical results, with uniformity and inter‐HD values close to 33.33%, indicating that the visual mapping of the security pattern does not influence the statistical performance of the PS‐TRNG.

After defining the two thresholds used for ternary digitization, we further analyzed how these boundary values were determined and how stable they remain under possible variations. The threshold values are selected from the measured current distribution of each device, where the three conduction levels associated with the ternary outputs appear as distinct clusters. The boundaries are placed at the separation regions between these clusters so that each read current can be reliably assigned to a specific ternary state. Since these thresholds arise from the measured electrical response of the device, they may shift under variations in operating conditions such as temperature drift or bias fluctuation. To evaluate how such threshold variations influence the evaluated figures of merit, we applied threshold shifts of ±5% and ±10% to the nominal values. These shifts are intended to emulate possible changes in the decision boundaries under environmental fluctuations. As shown in Figure S12, uniformity values exhibit gradual and predictable trends as the thresholds are shifted. The uniformity for trit ‘0’ ranged from 29.61% to 38.57%, for trit ‘1’ from 25.80% to 37.95%, and for trit ‘2’ from 32.44% to 35.62%. These variations reflect the redistribution of samples among the three ternary levels when the boundaries move, and the values fluctuate around the statistical ideal of 33.33% for a uniformly distributed ternary source. The inter‐HD remained close to its statistical ideal value, ranging between 33.31% and 34.31%, which corresponds to a maximum variation of approximately 1% relative to the expected value of about 33.33%. This result indicates that pairwise differences between ternary strings are not significantly affected by moderate threshold shifts. The entropy values ranged from 1.5466 to 1.5645, which is close to the statistical ideal log_2_3 ≈ 1.585. The degree of variation was small relative to the ideal value, suggesting that the overall information content of the ternary output remains stable. These results clarify that while threshold shifts cause predictable changes in the symbol balance, the core randomness indicators (inter‐HD and entropy) remain near their statistical ideal values. This classification produced about 15 000 ternary data points in 2000 s (Figure 2a), with distributions visualized via 32 × 32 contour maps (Figure 2b,c). The obtained logic states were evenly distributed, indicating unbiased randomness. The speed at this point is 7.5 trit/s, and based on the entropy conversion relation, this equals approximately 11.89 bit/s.

For a more rigorous assessment of the quality of randomness, we evaluated the output of the PS‐TRNG using the NIST statistical test suite. In the absence of a standardized framework for evaluating ternary random numbers [34, 35], the randomness of the generated output was assessed through an indirect approach. To enable evaluation using the NIST test suite, the ternary random number stream was decomposed into three binary subsets, as illustrated in Figure 2f. About 570 000 ternary trits were reorganized into three pairwise groupings: ‘0’ and ‘1’, ‘1’ and ‘2’, and ‘0’ and ‘2’. The resulting datasets yielded over 1.1 million binary bitstreams, allowing us to have a sufficiently high data density for reliable statistical evaluation. As shown in Figure 2g and Figure S18, all 45 evaluations (15 tests applied to each of the three binary subsets) passed the NIST test suite with p‐values exceeding the threshold of 0.01, confirming that the output exhibits the characteristics of true randomness (detailed results are provided in Tables S1–S3). The PRNG employed in this system functions only as an auxiliary controller to randomize the sampling instants and is not involved in entropy generation. To suppress the deterministic periodicity imposed by the pulsed‐light excitation, pseudorandom sampling signals generated by the PRNG were applied to the readout circuit. This sampling procedure only determines the timing of the measurement and does not influence or replace the stochastic trapping and de‐trapping of photo‐generated carriers, which remain the sole physical source of entropy in the system [36, 37]. This dispersed sampling timing amplifies the observable variations produced by stochastic trapping‐de‐trapping events, enabling robust ternary randomness. Since the entropy originates from intrinsic device physics, knowledge of the PRNG does not allow an attacker to predict the resulting random outputs. As a baseline comparison, the control input from the PRNG used in the sampling process was evaluated using the same NIST test suite, as shown in Figure 2h,i. The results showed that 4 out of 15 tests failed, indicating the limitations of PRNGs, which use deterministic algorithms instead of true entropy sources [38, 39, 40]. A detailed summary of the results is provided in Table S4, and the specifications of the circuit components used in the measurement system are listed in Table S5.

To further evaluate the security of the proposed PS‐TRNG, we conducted additional modeling‐attack simulations using Fourier‐basis regression and LSTM‐based recurrent neural networks. Both models failed to predict the generated trits beyond random‐guessing accuracy, indicating that no exploitable temporal or statistical patterns exist in the output stream. A detailed description of the analysis pipeline and full quantitative results are provided in Note S4 and Figure S19. The statistical independence of the generated ternary random outputs was further verified by autocorrelation function (ACF) analysis. As described in Note S5 and Figure S20, the ACF profile exhibited symmetric fluctuations around zero without distinct periodicity, confirming the absence of temporal correlation in the generated sequences. Additionally, we performed further analysis to compute the minimum entropy for each sampled trit through the Most Common Value (MCV) estimator described in NIST SP 800–90B. This estimator quantifies the worst‐case predictability of the source by measuring the information contained in each trit, thereby providing a conservative entropy estimate beyond standard NIST statistical testing. As shown in Figure S21, the values derived from 5 windows produced a mean minimum entropy of 1.58472 bits/trits, a standard deviation of 2.34 × 10–5 bits/trits. The lowest observed value of 1.58467 bits/trits corresponding to >99.98% of the theoretical upper bound, log_2_3 (≈ 1.58496) bits/trits. These results suggest that the proposed ternary TRNG exhibits statistically stable and near‐uniform randomness.

**FIGURE 3:**
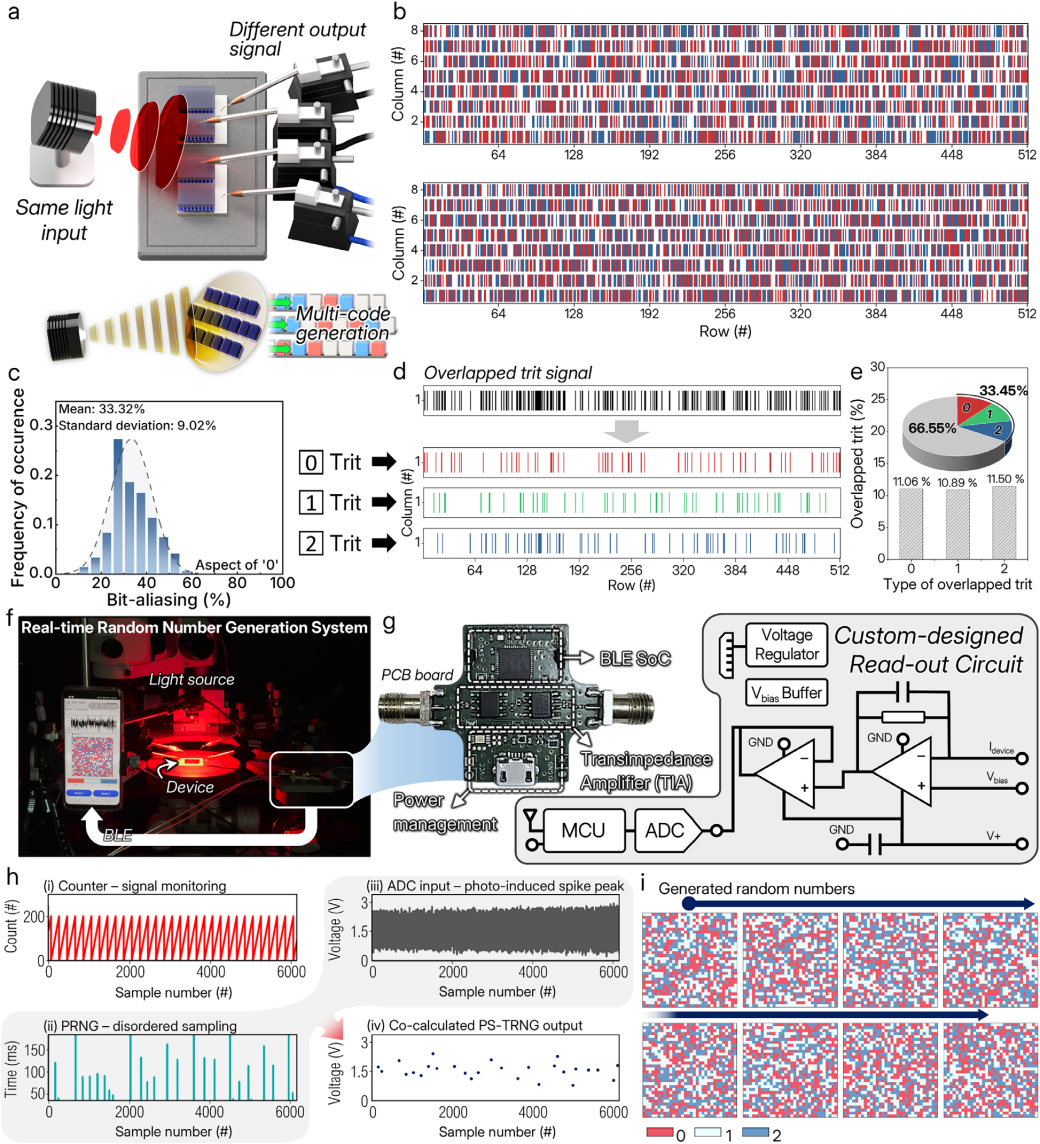
Intrinsic Entropy Validation of PS‐TRNG through Multi‐Device Comparison, and circuit‐level integration of the device. (a) Parallel operation of multiple PS‐TRNG devices under identical illumination, and a conceptual diagram of their simultaneous multi‐code generation for future scalable architectures. (b) Comparison of random numbers generated in parallel from two distinct PS‐TRNG devices (4096 data points). (c) Validation of bit‐aliasing between two random number data sets. (d) Classification of overlapping data from two data point streams. (e) Internal distribution of overlapping random number data. (f) Photograph of the integrated system connecting the PS‐TRNG device and the readout circuit, along with (g) a photo and schematic of the circuit used for integration. (h) Processing sequence of random number data within the customized circuit. (i) 32 × 32 mapping image of the output random numbers.

While this statistical verification confirmed the reliability of the generated randomness, the physical origin of the entropy remained to be clarified. In particular, it was necessary to determine whether the randomness stemmed from intrinsic device dynamics or from external optical excitation. Therefore, to identify the source of the observed randomness, we compared the outputs of two independent PS‐TRNG devices operated under identical light pulse conditions (Figure 3a). The distributions of 4096 ternary random numbers generated by each device, shown in Figure 3b, revealed no apparent correlation. Each output followed a unique path, shaped not by the light that triggered them but by the internal disorder of the system. Quantitative analyses using direct matching, statistical comparison, and bit‐aliasing (Figure 3c–e) further supported this independence. In Figure 3d, the overlap of 512 synchronized data points showed a uniform spread across logic values. The overall overlap ratio of 33.45% closely approached the theoretical value. Individual logic levels contributed nearly evenly, with values of 11.06, 10.89, and 11.5% for ‘0’, ‘1’, and ‘2’, respectively. These observations suggest that the entropy in the PS‐TRNG output is not imposed by the input signal but instead emerges from within the device itself, shaped by stochastic charge dynamics that resist replication or prediction.

To examine the relationship between independently generated outputs, we performed a bit‐aliasing analysis on two ternary random number streams, as shown in Figure 3c and Figure S22. Each of the 10 240 data points was grouped into twelve security key combinations, yielding a bit‐aliasing ratio of 33.32%. For comparison, binary random numbers ideally exhibit a 50% mismatch across twelve bits, while the ternary system used here produces a mismatch closer to three bits out of twelve. This greater variation strengthens resistance to key duplication and can offer improved security in high‐dimensional encoding schemes [35]. The observation that two devices, operating under identical optical input, produce entirely distinct random outputs supports the conclusion that entropy arises from internal device characteristics rather than the light source.

To validate the unpredictability, independence, and scalability of the parallel measurement scheme using a larger statistical sample, 16 samples were evaluated under identical illumination conditions by organizing them into four parallel groups of four (Figure S23). The uniformity, inter‐HD, and entropy values obtained from 4096 random data points generated by each sample are summarized in Figure S23a–g, all of which are close to their respective statistical ideals. Based on these results, bit‐aliasing analysis performed within each group showed that all four groups exhibited ratios close to the ideal value of 33.33%, confirming that each sample produced statistically independent random outputs under identical optical input conditions (Figure S24). To further evaluate the linear and nonlinear dependencies among the generated bitstreams, Pearson correlation coefficient and mutual information analyses were performed [41]. As summarized in Figure S23h,i, the mean values of all sets remained within the criteria of |ρ| < 0.05 and I <10^−3^ bits, and no individual set exhibited any outlier beyond these thresholds. These results confirm that all concurrently measured samples produced unpredictable and statistically independent random outputs. Detailed analyses for each of the 16 samples are provided in Figures S25–S28. We performed an intra‐HD evaluation by dividing 1 024 000 generated trits into 1024 individual 32 × 32 maps and comparing them pairwise. Except for the self‐matched cases along the diagonal, all map‐to‐map comparisons yielded the ideal Hamming distance of 66.66%, confirming that every mapping is statistically distinct from the others (Figure S29). Then, based on the two‐device parallel measurement scheme, we extended the setup to simultaneously measure more devices under the same optical source. This approach not only proportionally increased the random number generation rate but also allowed us to verify whether the independent randomness observed in individual devices remained consistent across multiple devices. Figure S30a presents the eight‐channel measurement circuit and the corresponding parallel‐measurement schematic, designed to enable simultaneous acquisition of random outputs from eight devices. Based on this configuration, we evaluated both the generation rate and the statistical independence of the produced random numbers. Figure S30b visualizes the combined output of 65 536 data points (8 channels × 8192 points each) as a 256 × 256 mapping obtained over 420 s. The measured generation rate reached 156.15 trit/s, confirming that the throughput increased with the number of operating devices. As shown in Figure S30c,d, both the uniformity and inter‐HD values were close to the ideal ternary distribution. The uniformity values were 32.42%, 33.27%, and 34.32% for ‘0’, ‘1’, and ‘2’, respectively, while the inter‐HD were 32.32%, 33.49%, and 34.13%, consistent with theoretical expectations. Furthermore, to examine potential correlations among the eight generated random sequences, the Pearson correlation coefficient and mutual information were calculated, as shown in Figure S30e,f. The two metrics respectively evaluate linear and nonlinear dependencies, and all 28 pairwise combinations exhibited stable values within the ideal range (|ρ| < 0.05 and I < 10–3 bits), confirming the absence of inter‐channel correlation. Additionally, the entropy of the generated random sequences reached 1.578 (Figure S30g), which is close to the theoretical ideal of 1.585, demonstrating uniform and stable entropy generation across the parallel architecture.

Building upon this evidence of device‐intrinsic entropy, we further examined the spectral and temporal robustness of the PS‐TRNG. We evaluated its response to optical stimulation at various wavelengths, including green (530 nm), blue (455 nm), UV‐A (365 nm), and UV‐B (310 nm) pulses. In all cases, the device consistently produced ternary outputs with inter‐HD values close to the theoretical 33.33% and entropy values approaching the ideal 1.585, confirming its wavelength‐independent entropy performance (Note S6 and Figures S31–S35). Long‐term stability was verified over a period of 460 days and more than two million measurements, during which the entropy characteristics remained stable (Figure S36), underscoring the device's practical viability for secure and persistent operation. Furthermore, the PS‐TRNG was also evaluated under various biasing modes (Figures S37–S40) and environmental conditions, including temperature, humidity, mechanical vibration, and other related factors. The device maintained consistent operational characteristics across all conditions, demonstrating its robustness under diverse practical environments. Detailed results are provided in Note S7 and Figures S41–S55.

As a step toward practical implementation, the proposed PS‐TRNGs were integrated with a custom‐designed printed circuit board (PCB) that wirelessly communicates with a commercial mobile device via low‐power Bluetooth (BLE) (Figure 3f,g). The PCB includes a power management unit for energy supply, a transimpedance amplifier (TIA) for analog signal acquisition, and a BLE system‐on‐chip (SoC) for digital signal processing. The photospike current generated by the PS‐TRNG in response to optical stimulation is captured by the TIA and amplified within a 0–3.3 V range. Based on predefined thresholds, signals below 1.44 V were classified as ‘0’, above 1.87 V as ‘2’, and those in between as ‘1’, forming ternary random values. Figure 3h illustrates this sequence of processes. The second and third panels of Figure 3h show the arbitrarily assigned readout timing and the time‐resolved continuous data points generated by the system, respectively. Consequently, the proposed system generates random number data in the form shown in the fourth panel of Figure 3h, representing the intersection of the graphs from the second and third panels. The digitized outputs were subsequently transmitted in real time to a paired mobile device via BLE, enabling continuous and wireless delivery of entropy signals. As a result, eight distinct datasets of ternary‐mapped outputs were successfully obtained (Figure 3i).

**FIGURE 4:**
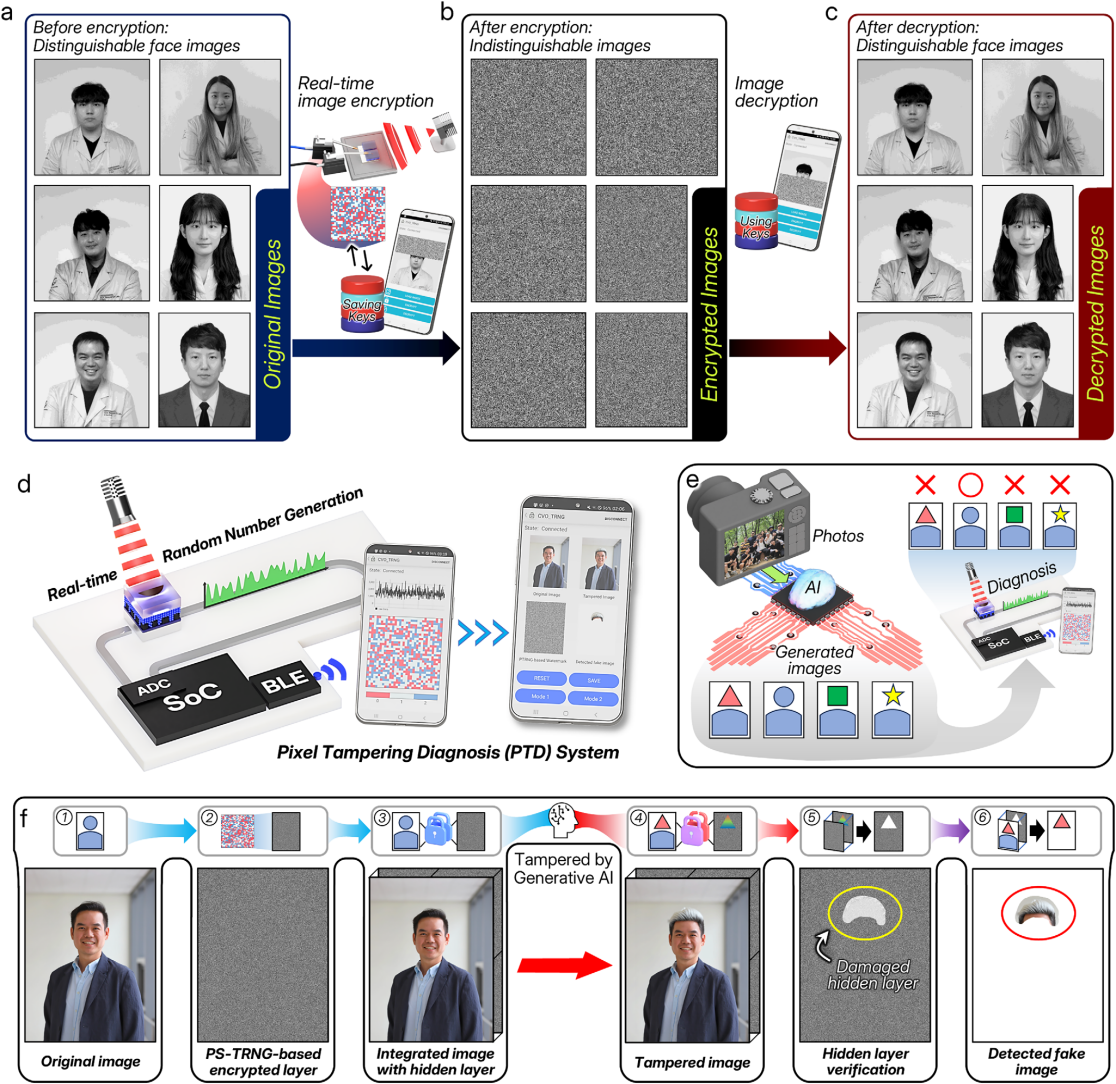
Real‐time image encryption/decryption and PTD system demonstration using circuit‐level integration. Image encryption/decryption using generated random numbers: (a) Original images of the authors; (b) Encrypted image; (c) Decrypted images of the authors. (d) Schematic illustration of the operation of the proposed PTD system. (e) Conceptual schematic of a future PTD system operating at the image acquisition level. (f) General workflow of the PTD system for detecting tampered images of a portrait of the author, Hocheon Yoo. Images of individuals are used with consent.

Having verified the true randomness of the PS‐TRNG outputs through comprehensive statistical analysis, including the NIST test suite, we next explored their practical applicability. Accordingly, building upon the circuit‐level integration of the PS‐TRNG, we implemented an Advanced Encryption Standard (AES)‐based image encryption application utilizing the generated random numbers, as demonstrated in Figure 4. Image encryption is a basic application widely adopted to protect personal information from unauthorized or malicious access. As a proof of concept, we performed real‐time random number generation and used the resulting data to encrypt images, as demonstrated using the facial images of the authors in Figure 4a–c and Movie S1. This process was performed in real‐time on each individual pixel of the grayscale images using the AES algorithm [42]. The randomly generated numbers were utilized to create a secret key for encryption. As a result, the original images were encrypted and visually obfuscated, rendering the individuals' faces and features unrecognizable, as shown in Figure 4b. However, when the stored random number keys were used for decryption, the images were fully restored to their original form, identical to those shown in Figure 4a, as presented in Figure 4c.

We also implemented the PTD system that embeds physically unclonable entropy directly into the image. As shown in Figure 4d–f, random numbers generated by the PS‐TRNG are used to construct an encrypted layer that mirrors the resolution of the original image. During the process, information of each pixel in the encryption layer is distributed and inserted into the two least significant bits (LSBs) of the corresponding original image pixel components. We employed an image steganography technique for data concealing. The embedding process preserves visual fidelity while introducing a hidden layer that is cryptographically verifiable and resistant to replication. Details of the embedding structure and pixel‐level bit distribution are provided in Note S8 and Figures S56–S60 and Table S6.

To evaluate its robustness, we used a generative AI tool to subtly alter the hairstyle of the subject in the hidden‐layered image. The modified output appeared visually authentic, yet the hidden layer revealed clear signs of tampering. Because the embedded information originates from true random numbers, it cannot be regenerated or imitated, even by advanced AI systems. As shown in Stages 5 and 6, the system accurately localized the altered region, demonstrating sensitivity down to the pixel level. In principle, even a single‐bit change is detectable. To further confirm this sensitivity of the PS‐TRNG‐based image verification system, we investigated its ability to detect small‐scale modifications in background features rather than large hairstyle changes. As shown in Figure S61, a PS‐TRNG‐generated random watermark was embedded into an original image containing a single flying bird. The image was then intentionally modified by adding three additional birds to form a minor tampered region. The system successfully identified the altered area by detecting localized damage in the embedded watermark, demonstrating that even small‐scale or subtle image modifications can be effectively recognized. The full demonstration, including real‐time image encryption, tampering, and pixel‐level detection, is provided in Movie S2, which further highlights the reliability of our system in detecting AI‐based image tampering. **[R2Q5]** Collectively, these results demonstrate a seamless transition from device‐level entropy generation to system‐level secure image verification, highlighting the practical viability of the PS‐TRNG platform.

## 3 Conclusions

In summary, we showed a PS‐TRNG, where stochastic charge trapping and de‐trapping dynamics within oxide heterojunctions produce hardware‐rooted entropy. The proposed system demonstrated stable statistical performance, achieving values close to the ideal 33.33% in both uniformity and inter‐HD. During the evaluation, the proposed device successfully passed all 15 statistical tests specified by NIST, confirming its robustness and reliability. Through a series of validation experiments, we further confirmed the uniqueness of the entropy source by showing that device‐specific random outputs are produced even under identical optical inputs, verifying its physically unclonable nature. A custom‐designed readout circuit enables real‐time digitization and wireless transmission of the ternary outputs, supporting downstream applications such as AES‐based image encryption and pixel‐level tampering detection. With device‐level entropy shown in this work, the proposed approach supports real‐time image verification and strong defense against deepfakes, guiding future on‐device security. While the proposed PS‐TRNG and PTD system demonstrate robust performance across diverse operating conditions, several remaining limitations also highlight clear directions for future development. The current LSB‐based embedding scheme enables pixel‐level tamper localization but remains sensitive to lossy compression and certain image‐processing operations that may degrade the hidden layer. Although lossless format conversion and adaptive hidden‐layer resizing preserve functionality, broader robustness across common processing pipelines will require further refinement. Approaches such as frequency‐domain or hybrid multi‐domain embedding, together with more advanced local statistical models, represent promising pathways for improving resilience and quantitative localization accuracy. These considerations outline a clear path for enhancing the practicality, scalability, and system‐level applicability of the proposed approach in future on‐device security systems. The operating speed of PS‐TRNGs can be further advanced through continued improvements in optical driving schemes and readout circuit design, including the use of faster and more efficient light sources. Such developments are expected to enhance the performance of individual devices and broaden their applicability. Beyond single‐device operation, the effective throughput can be increased by integrating multiple PS‐TRNGs into parallel array architectures. In this context, the proposed PS‐TRNG is well aligned with imaging and vision systems, in which random numbers are required locally across pixels or pixel groups, rather than being supplied by a single centralized high‐speed source. Accordingly, massive parallelism at the pixel or sub‐pixel level provides a natural pathway toward high‐throughput operation. Future work will focus on system‐level integration and optimization in order to maximize this potential while maintaining the intrinsic advantages of light‐induced entropy generation. Additionally, Table S7 presents a comparison and analysis of optical TRNG studies reported in recent years, focusing on key parameters such as entropy source, optical conditions, bit type, bit rate, power consumption, and stability.

## 4 Experimental Section

### 4.1 Materials and Chemicals

Copper(I) chloride (CuCl, 97%), 2‐methoxyethanol (CH_3_OCH_2_CH_2_OH, 99.8%), Ethanolamine (NH_2_CH_2_CH_2_OH, ≥99.5%), Tin tetrachloride (SnCl_4_, 98%), dichloromethane (CH_2_Cl_2_, ≥99.8%), *tert*‐butanol (C_4_H_10_O, ≥99.5%), chlorobenzene (C_6_H_5_Cl, ≥99.5%), chloroform (CHCl_3_, ≥99%), and 2‐propanol (C_3_H_8_O, IPA) were purchased from Sigma–Aldrich (USA). Ethanol (C_2_H_6_O, anhydrous, 99.8%) was supplied by Alfa Aesar (South Korea). Ammonium metavanadate (NH_4_VO_3_, 99%) was purchased from DAEJUNG (South Korea).

### 4.2 Preparing CVO NDs Precursor Solution and SnO2 QDs Solution (Synthetic Procedure)

To prepare the CVO NDs precursor solution, a vanadium precursor solution was prepared by dissolving 0.116 g of NH_4_VO_3_ (0.099 M) in 10 mL of deionized water. Separately, 0.099 g of CuCl (0.100 M) was dissolved in 10 mL of 2‐methoxyethanol to form the copper precursor. Each solution was stirred for 24 h after the addition of 0.2395 mL of ethanolamine, which served as a capping agent. The two precursor solutions were then mixed in a 1:1 volumetric ratio and stirred for an additional 1 h to obtain a homogeneous solution. For the synthesis of SnO_2_ QDs, 5.9 mL of SnCl_4_ was dissolved in 44.1 mL of dichloromethane and subsequently mixed with 100 mL of tert‐butanol. After stirring for 30 min at room temperature, the mixture was transferred into a 50 mL Teflon‐lined stainless‐steel autoclave and subjected to solvothermal treatment at 100°C for 24 h. The resulting colloidal solution was centrifuged at 4000 rpm for 10 min and washed three times with acetone to remove residual organic species. The purified SnO_2_ QDs were redispersed in a mixed solvent of chloroform and ethanol (3:1, v/v) at a concentration of 60 mg·mL^−1^.

### 4.3 PS‐TRNG Device Fabrication Process

The *n*‐Si substrates were cleaned and ultra‐sonicated in acetone and isopropyl alcohol (IPA) for 10 min and then dried on a hotplate at 100°C. The cleaned *n*‐Si substrate was UV‐ozone treated for 15 min, followed by a 0.5 mL drop of CVO NDs precursor solution. The precursor film was fabricated via spin‐coating at 3000 rpm for 30 s. Subsequently, the film underwent a two‐step calcination process: thermal annealing carried out in ambient air at 400°C for 2 h to promote precursor decomposition and oxidation; this was followed by a high‐temperature annealing step at 900°C for 2 h under an inert argon atmosphere to induce crystallization and phase formation. SnO_2_ QD films were spin‐coated at 3000 rpm, 30 s with 0.3 mL of SnO_2_ QD solution. After spin‐coating, they were annealed on a hotplate at 120°C for 30 min. UV‐ozone treatment was performed for 20 min to form PEDOT:PSS as the top electrode. It was spin‐coated at 2400 rpm for 30 s with 0.5 mL and annealed at 120°C for 30 min on a hotplate.

### 4.4 Characterization

The time‐dependent current signal of the PS‐TRNG was obtained using a Keithley 2400 semiconductor parametric analyzer, and all measurements were performed at room temperature. The cross‐section images of PS‐TRNG showing CVO NDs, SnO_2_ QDs, and lattice structure were obtained using TEM (JEM‐2100F, JEOL). The specimens were etched with a Ga^+^ ion beam from 30 to 5 keV using a focused ion beam and electron beam system (NX2000, Hitachi Ltd.). The crystal structure of the synthesized CVO NDs was analyzed using X‐ray diffraction (XRD, SmartLab, Rigaku). An atomic force microscope (AFM, NX‐10, Park Systems) was used to analyze the surface topology and work function of CVO NDs. The Kelvin probe force microscopy (KPFM) reference work function was set to a highly oriented pyrolytic graphite sample of 4.76 eV, using an ElectriMulti75‐G cantilever. The absorbance of SnO_2_ QDs was analyzed using a UV–vis spectrophotometer (Lambda 750, Perkin Elmer), and the optical bandgap was measured using a Tauc plot. Fermi levels of CVO NDs and SnO_2_ QDs were obtained from ultraviolet photoelectron spectroscopy (UPS, AXIS SUPRA, Kratos Inc.) analysis. The stability of the optical source was independently verified using a reference Si photodiode, confirming consistent intensity and periodicity without any spiking behavior (Figure S62).

### 4.5 Randomness Evaluation

The randomness of the security key patterns was evaluated in terms of uniformity, inter‐HD, and bit‐aliasing derived from the following mathematical models [43, 44, 45]:

(1) $$\mathrm{Uniformity}\;\;\left(\%\right)=\frac{1}{n}\sum_{i=1}^{n}r_{i}\times 100$$

where *r*_i_ is the number for bit ‘0’, ‘1’, or ‘2’, and *n* is the length of the security key. Uniformity is a security index representing the probability that the responses of the security keys are uniformly distributed as ‘0’, ‘1’, and ‘2’, indicating the randomness of the response bits. In the case of a ternary random signal, a uniformity of 33.33% demonstrates a high level of randomness

(2) $$\begin{array}{ccc} & & \mathrm{Inter}-\mathrm{HD}\;\left(\%\right)\\ & = & \frac{2}{k\left(k-1\right)}\sum_{i-1}^{k-1}\sum_{j=i+1}^{k}\frac{\mathrm{Hamming}\;\mathrm{distance}\;\left(R_{i},R_{j}\right)}{n}\times 100\; \end{array}$$

where *R_i_* and *R_j_* are the responses of the security keys *i* and *j* to the given challenge, respectively, *k* is the number of security keys and *n* is the string length of the security key. Inter‐HD is a measure of how unique the response of random patterns is when given the same challenge for ternary random trits, an ideal value of Inter‐HD is 33.33%.

(3) $$\mathrm{Bit}-\mathrm{Aliasin}g_{l}\left(\%\right)=\frac{1}{k}\sum_{i=1}^{k}R_{i}\left(j\right)\times 100\left[\%\right]$$

where *R_i_* (*j*) is the *j*th bit in an *n*‐bit response from the *i*th response of the security key. Bit‐aliasing indicates the similarity of responses from different security keys. When the responses have ternary values, an ideal scenario is that 33.33% of the responses are identical.

(4) $$H_{min,i}=-\log_{2}\left(\max\left(p_{0},p_{1},p_{2}\right)\right)$$

where *H_min,i_* represents the estimated minimum entropy of the *i*‐th window, and p_0_, p_1_, and p_2_, denote the empirical probabilities of the three possible output symbols (0, 1, and 2), respectively. The raw output sequence, consisting of 500 k trit, was divided into 5 non‐overlapping segments of 100 k trit each, and the per‐trit minimum entropy of each segment was calculated using the following equation [46].

### 4.6 LFN Measurement Method

LFN measurements were performed using a transimpedance amplifier and dynamic signal analyzer under DC biases of 0.05, 0.075, and 0.1 V. The current time traces were acquired in the dark and under pulsed illumination, then converted into power spectral density by Fourier transformation. The normalized *SI*/*I* ^2^ spectra were examined to identify Lorentzian contributions associated with interface trap dynamics [47].

### 4.7 Pearson Correlation Coefficient and Mutual Information Calculation

The cross‐correlation between two binary subsets was quantified using the Pearson correlation coefficient at zero lag, which is equivalent to the phi coefficient for binary variables. For two binary streams *X* and *Y*, the coefficient is given by

(5) $$\rho_{XY}=\frac{\sum_{i=1}^{n}\left(x_{i}-\bar{x}\right)\left(y_{i}-\bar{y}\right)}{\sqrt{\sum_{i=1}^{n}{\left(x_{i}-\bar{x}\right)}^{2}}\sqrt{\sum_{i=1}^{n}{\left(y_{i}-\bar{y}\right)}^{2}}}$$

where *x_i_* and *y_i_* are the bit values (0 or 1), and $\bar{x}$ and $\bar{y}$ are their respective mean values. As shown in Figure S58b, the resulting |ρ| values were below 0.05 for all 15 subset pairs, indicating negligible linear dependence.

Mutual information (MI) between the binary subsets was calculated based on Shannon entropy, defined as

(6) $$I\;\left(X;Y\right)=\sum_{x\in X}\sum_{y\in Y}p\left(x,y\right)log_{2}\left(\frac{p\left(x,y\right)}{p\left(x\right)p\left(y\right)}\right)\;$$

Where *p*(*x*, *y*) is the joint probability distribution of variables *X* and *Y*, and *p*(*x*) and *p*(*y*) are their marginal distributions.

## Conflicts of Interest

The authors declare no conflicts of interest.

## Supporting information




**Supplemental File 1**: adma72319‐sup‐0001‐SuppMat.docx.

**Supplemental File 2**: adma72319‐sup‐0002‐Supplementary Movie 1.mp4.

**Supplemental File 3**: adma72319‐sup‐0003‐Supplementary Movie 2.mp4.

## Data Availability

The data that support the findings of this study are available from the corresponding author upon reasonable request.
