# Supplementary material for: Light‐Induced Entropy for Secure Vision
Source: Adv Mater. 2026 Jan 29;38(33):e16947. doi: 10.1002/adma.202516947 (PMC13261377; doi:10.1002/adma.202516947)
Supplement: Supplementary file 1 — Supplemental File 1: adma72319‐sup‐0001‐SuppMat.docx. [file ADMA-38-e16947-s002.docx]

Supporting Information

**Light-Induced Entropy for Secure Vision**

Juhyung Seo^1,†^, Seungme Kang^1,†^, Chaehyun Kim^2,†^, Taehyun Park^1,†^, Youngwoo Yoo^2,†^,
Yeong Kwon Kim^3^, Wonjun Shin^4^, Byung Chul Jang^3,*^, Young-Joon Kim^2,5,*^, and Hocheon Yoo^1,*^

J. Seo, S. Kang, T. Park, Prof. H. Yoo
^1^Department of Electronic Engineering, Hanyang University, Seoul 04763, Republic of Korea

^*^E-mail: hocheon@hanyang.ac.kr

C. Kim, Y. Yoo, Prof. Y-J Kim
^2^Department of Semiconductor Engineering, Gachon University, 1342 Seongnam-daero, Seongnam, Gyeonggi-do, 13120, Korea

^*^E-mail: youngkim@gachon.ac.kr

Y.K. Kim, Prof. B.C. Jang
^3^School of Electronic and Electrical Engineering, Kyungpook National University, Daegu 41566, Republic of Korea

^*^E-mail: bc.jang@knu.ac.kr

Prof. W. Shin

^4^Department of Electrical and Computer Engineering, Sungkyunkwan University (SKKU), Suwon 16419, Republic of Korea

Prof. Y-J Kim
^5^Department of Electronic Engineering, Gachon University, 1342 Seongnam-daero, Seongnam, Gyeonggi-do, 13120, Korea

^*^E-mail: youngkim@gachon.ac.kr

^†^These authors contributed equally to this work.

Keywords: True Random Number Generator; Photospike; Image Security; Stochastic Electronics; Photodetectors

**<Table of Contents>**

**Supporting Note**

Supporting Note 1. Morphological and crystallographic characterization of CVO NDs and SnO_2_ QDs. S6

Supporting Note 2. Investigation of the energy band diagram of the PS-TRNG device. S7

Supporting Note 3. Analysis of trapping/de-trapping dynamics using low-frequency noise. S9

Supporting Note 4. Resistance of the PS-TRNG to Machine-Learning-Based Modeling Attacks. S11

Supporting Note 5. Autocorrelation analysis of ternary random numbers. S13

Supporting Note 6. Randomness analysis of ternary random numbers by multiple light sources. S14

Supporting Note 7. Environmental stability and operational robustness of the PS-TRNG. S15

Supporting Note 8. Process description of the pixel tampering diagnosis system. S17

**Supporting Figures**

Figure S1. Crystallographic and morphological characterization of CVO NDs and SnO_2_ QDs. S20

Figure S2. The crystal structure of the SnO_2_ QDs by XRD analysis. S21

Figure S3. The crystal structure of the CVO NDs by XRD analysis. S22

Figure S4. Photoresponse of the device under periodic light stimulation with 20 ms on/off cycles. S23

Figure S5. Optical and electronic structure characterization of SnO_2_ QD. S24

Figure S6. Surface potential analysis of CVO NDs using KPFM. S25

Figure S7. Energy Band diagram of PS-TRNG device. S26

Figure S8. Schematic illustration of the photospike sequence in the PS-TRNG device. S27

Figure S9. Schematic illustration of irregular photoluminescence peaks induced by defect states. S28

Figure S10. Frequency-dependent *S_I_*/*I*² characteristics under dark and illuminated conditions. S29

Figure S11. *S_I_*/*I*² behavior under repeated on/off illumination cycles. S30

Figure S12. Randomness characteristics of PS-TRNG outputs under threshold variation. S31

Figure S13. Signal-to-noise ratio characteristics under illuminated and dark conditions. S32

Figure S14. Schematic of the iterative downscaling process from 64 × 64 to 16 × 16 matrices. S33

Figure S15. Uniformity analyses for the 64 × 64, 32 × 32, and 16 × 16 matrices. S34

Figure S16. Inter-HD and entropy analyses for the 64 × 64, 32 × 32, and 16 × 16 matrices. S35

Figure S17. Detailed evaluation of the randomness characteristics of the generated trits. S36

Figure S18. NIST statistical test results for the proposed ternary random numbers. S37

Figure S19. Machine-learning-based modeling attack analysis demonstrates the PS-TRNG’s resistance to predictive algorithms. S38

Figure S20. Autocorrelation analysis confirms the absence of temporal correlation in the ternary random sequence. S40

Figure S21. Minimum-entropy evaluation of five 100-k-trit segments using the NIST SP 800-90B MCV estimator. S41

Figure S22. Validation of bit-aliasing between parallel output data sets. S42

Figure S23. Statistical evaluation of ternary random sequences from sixteen samples. S43

Figure S24. Bit-aliasing analysis within sample groups #A–#D. S44

Figure S25. Statistical comparison of simultaneously generated bitstreams in group #A. S45

Figure S26. Statistical comparison of simultaneously generated bitstreams in group #B. S48

Figure S27. Statistical comparison of simultaneously generated bitstreams in group #C. S51

Figure S28. Statistical comparison of simultaneously generated bitstreams in group #D. S54

Figure S29. Intra-HD heatmap confirming statistical independence across 1,000 ternary mappings. S57

Figure S30. Parallel operation of eight-channel PS-TRNG and randomness assessment. S58

Figure S31. PS-TRNG randomness and security-code generation under diverse illumination wavelengths. S59

Figure S32. Visible-light-induced photoresponse characteristics of the PS-TRNG. S60

Figure S33. Ultraviolet-light photoresponse characteristics of the PS-TRNG. S61

Figure S34. Inter-HD characteristics of PS-TRNG outputs under visible and UV illumination. S62

Figure S35. Uniformity analysis of PS-TRNG outputs under visible and UV illumination. S63

Figure S36. Long-term stability and randomness behavior of the PS-TRNG after 460 days. S64

Figure S37. Bias-dependent uniformity of ternary outputs under positive bias modes. S65

Figure S38. Bias-dependent inter-HD and entropy characteristics under positive bias modes. S66

Figure S39. Bias-dependent uniformity of ternary outputs under negative bias modes. S67

Figure S40. Bias-dependent inter-HD and entropy characteristics under negative bias modes. S68

Figure S41. Temperature-dependent uniformity of PS-TRNG ternary random outputs. S69

Figure S42. Temperature-dependent inter-HD and entropy characteristics of the PS-TRNG. S70

Figure S43. Humidity-dependent uniformity characteristics of the generated ternary random numbers. S71

Figure S44. Humidity-dependent inter-HD and entropy characteristics of the PS-TRNG. S72

Figure S45. Randomness stability of the PS-TRNG under mechanical vibration. S73

Figure S46. Uniformity characteristics under varying optical power densities. S74

Figure S47. Inter-HD and entropy characteristics under varying optical power densities. S75

Figure S48. Uniformity characteristics under symmetric optical pulse conditions. S76

Figure S49. Inter-HD and entropy characteristics under symmetric optical pulse conditions. S77

Figure S50. Uniformity characteristics under asymmetric optical pulse conditions (off modulation). S78

Figure S51. Inter-HD and entropy characteristics under asymmetric optical pulse conditions (off modulation). S79

Figure S52. Uniformity characteristics under asymmetric optical pulse conditions (on modulation). S80

Figure S53. Inter-HD and entropy characteristics under asymmetric optical pulse conditions (on modulation). S81

Figure S54. Randomness evaluation of the PS-TRNG under ambient indoor light. S82

Figure S55. Randomness evaluation of the PS-TRNG under natural sunlight. S83

Figure S56. Pixel tampering diagnosis system using PS-TRNG-based real-time ternary encryption. S84

Figure S57. Inter-HD, uniformity, and entropy of binary subsets derived from ternary pairs S1–S3. S85

Figure S58. Correlation analysis of binary subsets derived from the ternary sequence. S86

Figure S59. Robustness of the PTD system under image format conversion and compression. S87

Figure S60. Robustness of the PTD system under image resizing and filtering. S88

Figure S61. Demonstration of PS-TRNG-based watermark generation and tamper detection. S89

Figure S62. Stability verification of the optical source using a reference Si photodiode. S90

**Supporting Table**

Table S1. NIST test suite results of PS-TRNG (Aspect of 0 and 1). S91

Table S2. NIST test suite results of PS-TRNG (Aspect of 1 and 2). S91

Table S3. NIST test suite results of PS-TRNG (Aspect of 0 and 2). S92

Table S4. NIST test suite results of binary PRNG. S92

Table S5. Readout circuit specifications for the PS-TRNG system. S93

Table S6. Evaluation of hidden-layer robustness under format conversion and filtering. S94

Table S7. Comparison of optical TRNGs covering optical conditions, random number generation capability, power consumption, and stability. S95

**Supporting Movie**

Movie S1. Demonstration of real-time ternary random number generation followed by image encryption and decryption using the PS-TRNG. S97

Movie S2. Demonstration of the PTD system operation using generated random numbers. S97

**Reference**

**Supporting Note 1: Morphological and crystallographic characterization of CVO NDs and SnO_2_ QDs.**

The surface morphology and crystal structure of the fabricated SnO₂ quantum dot (QD) thin films were characterized using topographical and crystallographic analyses. Nanodot-like surface patterns were observed after CuV₂O₆ (CVO) deposition on *n*-Si, as shown in Figure S1a,b, and further confirmed by atomic force microscopy (AFM) (Figure S1c). Cross-sectional TEM imaging clearly revealed the CVO nanodots (Figure S1d), and lattice-resolved TEM confirmed crystalline SnO₂ and Si phases (Figure S1e), supported by FFT analysis of the SnO₂ QDs (Figure S1f).

X-ray diffraction (XRD) analysis verified the crystallinity of the SnO₂ QDs, with diffraction peaks at 26.6° (110), 33.9° (101), 37.9° (200), 51.7° (211), and 64.7° (301), matching JCPDS 41-1445 (Figure S2). Crystallographic signatures of the CVO nanodots were also confirmed by XRD, with peaks at 22.0°, 27.9°, and 44.8°, corresponding to the (−102), (112), and (−204) planes of CuV₂O₆ (Figure S3), excluding other possible phases such as CuVO₂^[1]^.

**Supporting Note 2: Investigation of the energy band diagram of the PS-TRNG device.**

The optical and electronic properties of SnO₂ QDs and CVO NDs were systematically characterized to understand their roles in the heterostructure and charge carrier dynamics. The optical bandgap of the SnO₂ QDs was determined using ultraviolet-visible (UV–Vis) spectroscopy and Tauc analysis. As shown in Figure S5a, the Tauc plot indicates a direct bandgap of approximately 4.45 eV, which corresponds well with the strong absorption observed in the deep ultraviolet (DUV) region (Figure S5b). This widened bandgap is attributed to quantum confinement effects arising from the nanoscale dimensions of the QDs. Electronic energy levels were further investigated using ultraviolet photoelectron spectroscopy (UPS). In the case of SnO₂ QDs, the secondary electron cutoff and valence band region (Figure S5c) indicate a work function of 4.47 eV, with the valence band maximum (*E*_V_) located 4.21 eV below the Fermi level (*E*_F_). Based on this, the *E*_V_ relative to vacuum is approximately $-$8.68 eV, and the conduction band minimum (*E*_C_) can be estimated at $-$4.23 eV, considering the 4.45 eV bandgap. These values confirm the *n*-type nature of the SnO₂ QDs and are consistent with previously reported energy level configurations for SnO₂ QDs^[2]^.

In contrast, direct optical and photo-emission-based characterization of CVO NDs was not feasible due to the discontinuous nanodot morphology and limited film uniformity. Instead, the surface electronic structure of CVO was probed using kelvin probe force microscopy (KPFM). As shown in Figure S6a, localized work function mapping revealed consistent values across four distinct regions (Points A–D), ranging from 4.78 to 4.81 eV, with an average of approximately 4.80 eV (Figure S6b). This average surface work function was used as a reference for estimating the *E*_F_ position in CVO NDs. The *E*_C_ and *E*_V_ of CVO were adopted from previously reported values due to the absence of direct measurements^[3]^. Literature suggests that CuV₂O_6_ possesses an indirect bandgap ranging from 1.90 eV, with the *E*_V_ typically positioned 1.78 eV below the *E*_F_. The built-in electric fields-induced charge carrier dynamics were investigated through the energy band diagram derived from specific energy levels of each material.

As represented in Figure S8, in the absence of external bias and under light illumination, the photo-generated electron and hole pairs (EHPs) in *n*-Si are separated owing to the built-in electric fields applied at SnO_2_ QDs/CVO NDs/*n*-Si junctions, further confirmed by open circuit voltage (*V*_OC_) shifts shown in Figure S16a. Clearly distinguishable *V*_OC_ shifts were observed regardless of the wavelengths under zero bias condition, suggesting that the photo response of the proposed device is mainly originating from the built-in electric fields-induced photovoltaic effects.

**Supporting Note 3: Analysis of trapping/de-trapping dynamics using low-frequency noise.**

We performed low-frequency noise (LFN) measurements, which are a well-established technique to probe carrier transport dynamics and defect-related trapping processes in semiconductor junctions. LFN analysis offers a direct electrical means to quantify the fluctuation dynamics of charge carriers and to identify the presence of trap states and their characteristic time constants without requiring additional structural modification. LFN spectra were obtained from the proposed two-terminal device under various DC bias conditions. The current fluctuations were recorded in the time domain and converted into the power spectral density (PSD) using Fourier transformation. Measurements were conducted under both dark and illuminated conditions to decouple the role of photogenerated carriers from intrinsic trap-assisted conduction. By comparing the spectral features in both conditions, the influence of illumination on charge conduction and trap occupancy could be systematically analyzed.

In the dark condition, the normalized spectra are well described by a 1/*f* ^2^ noise with plateau in the 20–70 Hz band regardless of applied bias (0.05, 0.075, and 0.1 V), as shown in Figure S10. This feature is characteristic of a Lorentzian contribution from discrete traps (random telegraph noise (RTN) behavior) localized at the CVO NDs/SnO_2_ QDs interface. The plateau bounds yield a characteristic time-constant window via

$f_{c}=\frac{1}{2\pi\tau}\Rightarrow\tau=\frac{1}{2\pi f_{c}}\approx2.3\text{ ms (at 70 Hz) to }8.0\text{ ms (at 20 Hz)}$ (S1)

These millisecond time constants indicate traps that exchange carriers with the transport channel on experimental time scales and thus contribute dominantly to carrier-number fluctuations. Microscopically, in the dark the relevant interfacial states are largely empty, so carriers conducting the junction are intermittently captured and emitted, producing the observed RTN-like signature and the plateau in the normalized noise (*S*_I_/*I*^2^). Under illumination, on the other hand, two concomitant effects are observed: (i) the overall normalized noise magnitude decreases, and (ii) the 20–70 Hz plateau disappears. Both observations are consistent with photo-filling of the interfacial traps by electron–hole pairs: increasing the occupancy drives the binomial variance *p*(1-*p*) of trap occupancy toward zero, thereby suppressing carrier-number fluctuations and eliminating the Lorentzian corner. At the same time, once these interface centers are filled, carriers experience a more continuous conduction path across the junction, so the LFN becomes dominated by distributed background 1/*f* noise behavior rather than discrete capture–emission events in a localized region.

Moreover, the frequency range of the dark-state plateau (20–70 Hz) closely overlaps the device operating bandwidth used for photospike generation (50 Hz). This proximity indicates that the same interfacial trap kinetics that set the Lorentzian corner in the LFN spectra also modulate the spike-to-spike variability that underpins our random fluctuations. In other words, the LFN-derived trapping/de-trapping dynamics are operative on the very timescales relevant to our PS-TRNG, providing a direct physical link between the measured noise and the randomness source. Importantly, repeated acquisitions under identical conditions reproducibly show the dark-state plateau and its suppression under illumination, while the overall *S*_I_/*I*^2^ trends remain unchanged across runs (Figure S11). This repeatability, together with the bias-independent appearance of the plateau and its interfacial assignment, reinforces that the fluctuations originate from the CVO NDs/SnO_2_ QDs interface rather than from extrinsic instrumentation or environmental drift.

**Supporting Note 4: Resistance of the PS-TRNG to Machine-Learning-Based Modeling Attacks**

We have further conducted a modeling attack simulation using machine learning regression and recurrent neural network (RNN). The analysis pipeline consists of three steps: (i) key generation, (ii) test set preparation and modeling, and (iii) training and evaluation.

For our analysis, we used a dataset consisting of 300,000 trits obtained from a single TRNG device. The sequence was divided into 30 windows, each containing 10,000 trits. Each window was further partitioned into non-overlapping keys of 32 trits, achieving a total of 9,360 regression samples. Each model was trained on 70% of the sequence and validated on the remaining 30%, following the same protocol as previous modeling-attack studies^[4,5]^. The Fourier basis order was varied from n = 2 to n = 12, and representative results for n = 6 are shown in Figure S19a. The left panel of Figure S19a shows the trained segment, where the red regression curve partially follows the blue ground-truth trits, indicating that short-range statistical correlations in the training data can be represented by the regression basis. When the same model was applied to the unseen test set (right panel), however, the predicted sequence diverged from the actual output. Although the model maintained smooth continuity in its waveform, the predicted trits no longer matched the true data, and the accuracy dropped to 34.375%, which corresponds to random guessing in the ternary case. This result demonstrates that the learned model captures only limited local patterns and fails to generalize beyond the training data, consistent with the behavior expected from an ideal random source.

Figure S19b shows the per-bit prediction accuracy across all key segments and trit indices. The accuracy distribution remains close to random-guessing levels (mean: 33.20%, standard deviation: 5.26%), and no systematic bias or deterministic trend is observed. In Figure S19c, the averaged prediction accuracy across different Fourier regression orders (n = 2-12) remains statistically constant within the error range (standard deviation < 6.5%). These results demonstrate that increasing model complexity does not improve prediction performance, confirming the robustness of the TRNG output against spectral or regression-based modeling attacks.

To further assess the robustness of the TRNG output against more complex learning-based attacks, we additionally implemented a RNN model based on the long short-term memory (LSTM) architecture, as suggested in previous works^[5]^. Each segment of the 300,000-trit dataset used in the Fourier regression analysis was independently trained using the similar preprocessing pipeline (sequence length = 6), but the LSTM model was configured with one recurrent layer (128 units), a dense layer of 64 neurons (sigmoid activation), and a dropout rate of 0.2. The model was optimized using the Adam optimizer (learning rate = 0.001) and the weighted cross-entropy loss function, which compensates for potential class imbalance among ternary symbols (0, 1, 2). For each configuration, the dataset was divided into training and test subsets with varying ratios of 30%, 50%, and 70%, and each segment was trained independently from scratch without sharing model parameters across segments.

As shown in Figure S19d, the training curves demonstrate that the model converges rapidly within 50 epochs. The corresponding test accuracies remain in the range of 33–36% across all training ratios, which closely aligns with the theoretical probability of random guessing in the ternary space (1/3 = 33.3%). The confusion matrices (Figure S19e) further confirm that all three output classes appear with approximately uniform probability, and no systematic bias or deterministic trend is observed. Even when the training proportion was increased to 70%, the final test accuracy reached only 35.7%, respectively. This result indicates that the LSTM network could not extract any exploitable temporal correlation from the trit sequence (Figure S19f).

Through the above-described modeling simulation, we verified that even when exposed to machine learning attacks, the generated trit stream maintains statistical independence and unpredictability, validating its suitability for cryptographic and physical security applications.

**Supporting Note 5: Autocorrelation analysis of ternary random numbers.**

In this work, the random outputs were directly generated as ternary random symbols (0, 1, 2), and the analysis was conducted in the ternary domain without binary conversion to preserve the full probabilistic characteristics of the data. A sequence of 50,000 ternary trit was analyzed using the standard autocorrelation estimator:

$ACF \left( r_{k} \right)= \frac{\sum_{t=1}^{N-k} \left( x_{t}-\bar{x} \right)\left( x_{t+k}-\bar{x} \right)}{\sum_{t=1}^{N} \left( x_{t}-\bar{x} \right)^{2}}$ (S2)

where *x_t_* denotes the *t*-th ternary symbol in the sequence, 𝑥̄ is the sample mean of all *N* symbols, and *k* represents the lag. This formulation quantifies the linear dependence between symbols separated by *k* positions. The ACF was computed up to lag = 500, and the 95% confidence interval (CI) for uncorrelated data was defined as ±1.96 / $\sqrt{N}$ ≈ ±0.0087. As represented in Figure S20, among the 500 lag values, 20 (4%) slightly exceeded this CI, which is fully consistent with the statistical expectation under the independence assumption (Binomial (500, 0.05), 95% prediction interval = 15 - 35). The overall ACF profile exhibited symmetric fluctuations around zero with no significant periodicity or drift, confirming that the generated ternary random sequence is statistically independent.

**Supporting Note 6. Randomness analysis of ternary random numbers by multiple light sources.**

We obtained ternary random numbers from different wavelengths of light sources and extracted inter-HD, uniformity, and bit-aliasing to analyze the randomness. The light sources used were green (530 nm), blue (455 nm), UV-A (365 nm), and UV-B (310 nm). Also, the number of ternary random numbers used in the randomization analysis was 4,096 data points for each wavelength. In Figure S34, we extracted the inter-HD of the ‘0’, ‘1’, and ‘2’ trits of the ternary random number output by switching each light source. As a result, the inter-HD values under the green (term of ‘0’: 33.53%, term of ‘1’: 32.99%, and term of ‘2’: 33.48%) and blue light (term of ‘0’: 34.85%, term of ‘1’: 31.86%, and term of ‘2’: 33.28%) pulse inputs show an ideal value close to 33%. Similarly, the inter-HD values under UV-A (term of ‘0’: 32.59%, term of ‘1’: 33.70%, and term of ‘2’: 33.70%) and UV-B light (term of ‘0’: 34.06%, term of ‘1’: 31.71%, and term of ‘2’: 34.21%) pulse environments also exhibit ideal values.

In addition, Figure S35 shows the uniformity of the ternary random numbers over the light conditions. Highly uniform values were commonly obtained for green (term of ‘0’: 33.52%, term of ‘1’: 32.96%, and term of ‘2’: 33.50%), blue (term of ‘0’: 34.84%, term of ‘1’: 31.79%, and term of ‘2’: 33.35%), UV-A (term of ‘0’: 32.62%, term of ‘1’: 33.60%, and term of ‘2’: 33.77%), and UV-B light (term of ‘0’: 34.03%, term of ‘1’: 31.60%, and term of ‘2’: 34.35%). The corresponding entropy values were measured to be 1.548 for green, 1.554 for blue, 1.545 for UV-A, and 1.543 for UV-B, all close to the theoretical maximum of 1.585, confirming consistent randomness across illumination conditions.

**Supporting Note 7: Environmental stability and operational robustness of the PS-TRNG.**

To verify the environmental stability of the PS-TRNG, the device was systematically tested under diverse conditions, including temperature, humidity, mechanical vibration, optical intensity, various optical pulse profiles, indoor ambient light, and natural sunlight. As shown Figures S41–S55, the ternary random generation characteristics remained stable across all tested environments.

In Figures S41 and S42, the operational stability of the device was verified by performing random number generation at temperatures ranging from room temperature (RT) to 100 ℃ in 20 ℃ intervals. Temperature changes can directly affect the carrier mobility within the semiconductor and the trapping/de-trapping rates, potentially influencing the random number generation characteristics. However, even under high-temperature conditions up to 100 ℃, the ternary mapping characteristics and the distribution of the generated random numbers remained nearly identical, with no significant variation in the photospike current levels or probability distribution, except for a slight increase in the off current.

Furthermore, we performed the operation of the PS-TRNG under 50% and 80% relative humidity conditions (Figures S43 and S44). The results showed that the ternary mapping characteristics and the distribution of the generated random numbers remained nearly identical to those under dry ambient conditions, even in high-humidity environments. These results indicate that surface moisture adsorption or leakage path variations caused by humidity do not substantially affect the light-induced trap/detrap-based stochastic operation mechanism.

In addition to humidity, we also examined the device’s stability under mechanical vibration. To further evaluate the stability of the PS-TRNG under external vibration conditions, we set up an environment in which a 132 mW vibration module was operated 10 cm away from the device (Figure S45). Similarly, the results obtained from the ternary mapping characteristics and distribution analyses revealed no significant variation that could interfere with random number generation.

In Figures S46–S53, we evaluated the impact of light intensity and optical pulse parameters on the random output behavior. Figures S46 and S47 presents the random numbers generated under 660 nm illumination at five different optical power densities (optical power density = 0.17 mW·cm⁻², 0.35 mW·cm⁻², 0.53 mW·cm⁻², 0.71 mW·cm⁻², and 0.90 mW·cm⁻²). Furthermore, to examine the effect of temporally fluctuating optical inputs, the on/off pulse times were varied as shown in Figures S48–S53. The measurements were performed under three configurations: identical on/off timing (10 ms/10 ms, 30 ms/30 ms, 50 ms/50 ms), fixed on-time with variable off-time (10 ms/10 ms, 10 ms/30 ms, 10 ms/50 ms), and fixed off-time with variable on-time (10 ms/10 ms, 30 ms/10 ms, 50 ms/10 ms). As a result, despite changes in light intensity/pulse parameters, the uniformity, inter-HD, and entropy values of the generated random numbers remained close to their ideal values (33.33%, 33.33%, and 1.58, respectively). These results confirm that the PS-TRNG maintains stable ternary mapping characteristics and high randomness quality even under fluctuating optical input conditions.

As shown in Figure S54a, we first measured the random-number generation of the PS-TRNG under indoor ambient lighting. Since indoor lighting typically exhibits 60 Hz frequency, it could potentially degrade the quality of the generated randomness. However, as shown in Figure S55b,c, the 32 × 32 mapping of the random numbers generated under indoor lighting demonstrates that the trits are evenly distributed, and the calculated entropy was 1.553, which is close to the theoretical ideal of 1.585. In addition, the generated random numbers exhibited uniformity values of 33.69% (Aspect of ‘0’), 33.50% (Aspect of ‘1’), and 32.81% (Aspect of ‘2’), which are close to the ideal ternary value of 33.33%. The inter-HD also remained near the ideal value of 33.33%, yielding 33.67%, 33.13%, and 33.06%, respectively. These results confirm that indoor ambient light does not affect the entropy characteristics of the generated randomness.

Subsequently, we tested the device under natural sunlight (Figure S55), which provides a broadband illumination that could potentially influence the randomness quality. The generated random outputs exhibited stable statistical behavior, with an entropy value of 1.555 that is close to the theoretical ideal of 1.585. The uniformity values of 33.69% (Aspect of ‘0’), 33.50% (Aspect of ‘1’), and 32.81% (Aspect of ‘2’) and inter-HD values of 34.05%, 32.96%, and 32.54% also remained near the ideal 33.33%, confirming that the entropy characteristics of the TRNG are preserved under solar illumination and that environmental sunlight does not affect the entropy quality of the generated random signals.

**Supporting Note 8. Process description of the pixel tampering diagnosis system.**

In this work, the PTD system is designed to detect pixel-level tampering of authentic images by verifying the embedded hidden layer. Images generated entirely by AI do not contain this hardware-embedded layer, enabling them to be distinguished from genuine sensor-captured images within a proof-of-origin framework. Both encryption and insertion of the hidden layer process are implemented on the smartphone as shown in Figure S56a,b. The signal generated by the PS-TRNG is transmitted via Bluetooth Low Energy (BLE), enabling in two operation modes. The first mode displays a real-time signal graph based on the coded PRNG source. These data are partitioned into ternary random data according to the predefined threshold values, which are continuously updated into a 32 × 32 security code. The thresholds were determined as the values that ensure a constant ratio, calculated based on signals measured over a period of approximately 10 min.

In the second mode, the generated security code is utilized to perform image encryption and verification. The encryption process employs the Advanced Encryption Standard (AES) algorithm, utilizing only 128-bit of the security code to generate a single security key. This key was employed to encrypt and decrypt the entire image, thereby ensuring the secure protection of the image. Since the current AES algorithm lacks compatibility with ternary random numbers, we converted the generated random numbers from ternary to binary when applying them, including in the PTD system discussed later. To ensure a stable and unbiased ternary-to-binary conversion, we followed the same approach used in the NIST evaluation. Specifically, three binary subsets were extracted from the ternary sequence, corresponding to the trit pairs ‘0–1 (S1)’, ‘1–2 (S2)’, and ‘0–2 (S3)’, and were utilized as three independent binary random streams. All three subsets (S1, S2, and S3) exhibited inter-HD values of 49.76%, 49.65%, and 49.41%, respectively, which are close to the ideal 50 %. The corresponding uniformity values were 43.95%, 50.88%, and 45.41%, also approaching the ideal balance. The entropy of each subset reached 0.97 on average, approaching the theoretical maximum of 1 (Figure S57). In addition, to confirm that the three binary streams were statistically independent, we calculated the Pearson correlation coefficient and mutual information. These indicators evaluate the linear and nonlinear dependencies between random sequences, respectively. As shown in Figure S58, all pairwise combinations among the three subsets exhibited ideal values (|ρ| < 0.05 and I < 10⁻³ bits), confirming the absence of measurable correlation between them. Furthermore, the encrypted data was utilized for image verification by embedding the watermark to safeguard against forgery attempts by external attackers. To ensure the imperceptibility of the watermark, the least significant bit (LSB) modification method based on steganography was employed. This approach allows the embedded watermark to remain visually undetectable. As shown in Figure S56c-1, the original image comprised four components per pixel: alpha, red, green, and blue. The encrypted data in Figure S56c-2, generated as described above, was embedded within the two LSB of each pixel component, resulting in an image that remains visually indistinguishable from the original image (Figure S56c-3). resulting in an image that remains visually indistinguishable from the original image. Figure S56c-4 illustrates an image arbitrarily generated using a generative AI. To determine whether the given image had been altered by AI, the embedded watermark was extracted. By performing an exclusive-OR operation between the original watermark and the watermarks invisibly embedded into each pixel, distortions introduced during the AI alteration process were identified as shown in Figure S56c-5. Based on these distorted watermarks, the specific image transformations induced by the AI were characterized as shown in Figure S56c-6.

To further validate robustness, we tested the system under common image transformations, such as compression and resizing. As shown in Figure S59, we applied the same LSB-embedded hidden layer to a BMP-format image of a mug and then converted it into PNG format. This conversion primarily modifies the file structure and metadata without affecting the LSB domain. As a result, after compression and format conversion, the image quality remained unchanged while the file size was reduced by about 46%. As shown in Figure S59, the hidden-layer information embedded in the LSB domain remained stable after compression, and the detection system maintained reliable performance, accurately identifying the modification in the handle of the mug in the compressed PNG image. This stability was further confirmed in other file formats, including TIFF and WEBP, where the LSB domain remained unaffected during conversion. Results for other file formats are presented in Table S6. Meanwhile, in the filtering process that resamples the number of pixels through resizing, the system can mitigate performance degradation by applying adaptive hidden-layer resizing, as illustrated in Figure S60. In this approach, the hidden layer of the original image is first separated, then sliced and rescaled to match the resolution of the filtered image, and subsequently reinserted into the resized image. Although the image itself passes through a low-pass filter during the resizing process, the hidden layer stored in the LSB domain is independently adjusted outside the filtering pipeline, allowing it to be preserved without any loss. As a result, as shown in Figure S60, the system was able to reliably detect the modification in the lid of the mug in the resized and filtered image.

**
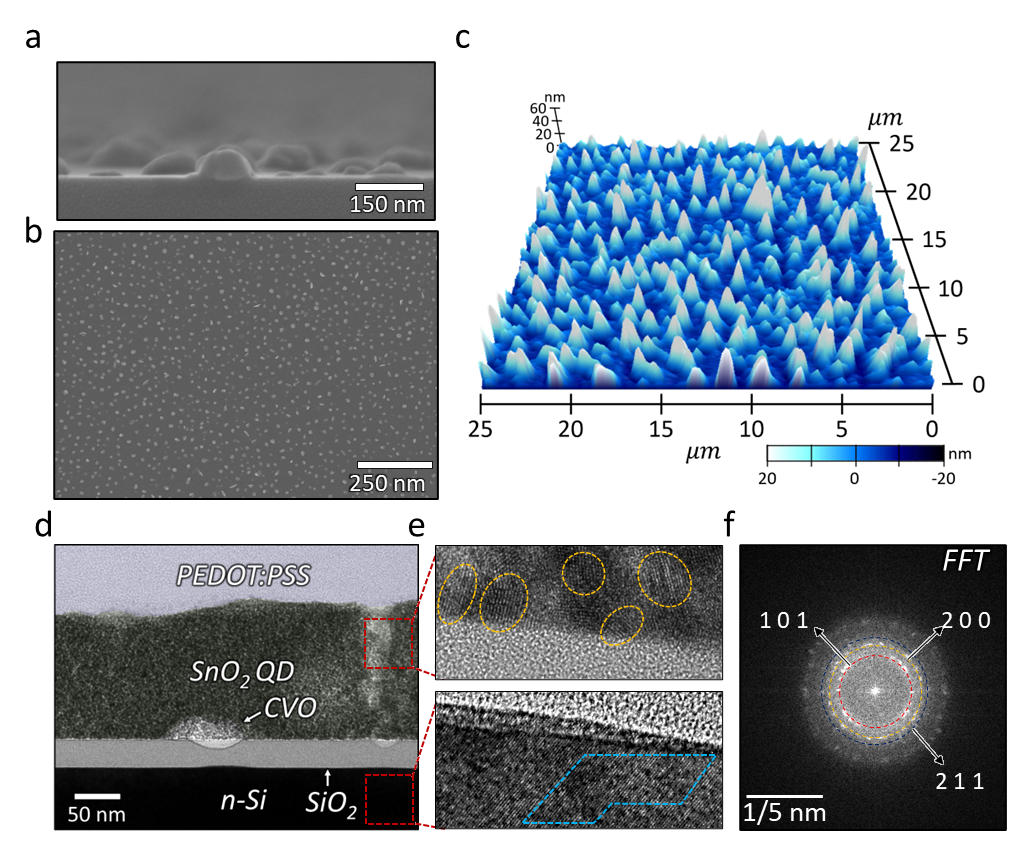
**

**Figure S1.** Crystallographic and morphological characterization of CVO NDs and SnO_2_ QDs. (a) Cross-sectional SEM image of CVO NDs, revealing the layered nanodot structure. (b) Top-view SEM image of the CVO NDs, showing their lateral distribution and morphology. (c) The surface of the CVO NDs through AFM phase analysis. (d) Cross-sectional TEM image of the PS-TRNG device, visualizing the internal stack structure. (e) High-resolution TEM image showing the lattice structure of the Si and SnO₂ layers. (f) FFT pattern corresponding to the SnO₂ QD region, confirming its crystallographic orientation.


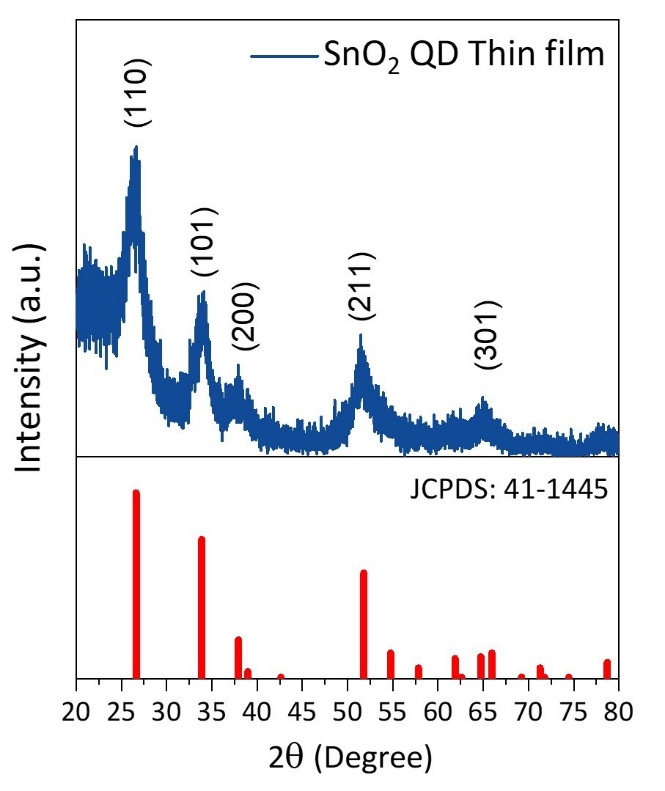


**Figure S2.** The crystal structure of the SnO_2_ QDs by XRD analysis.


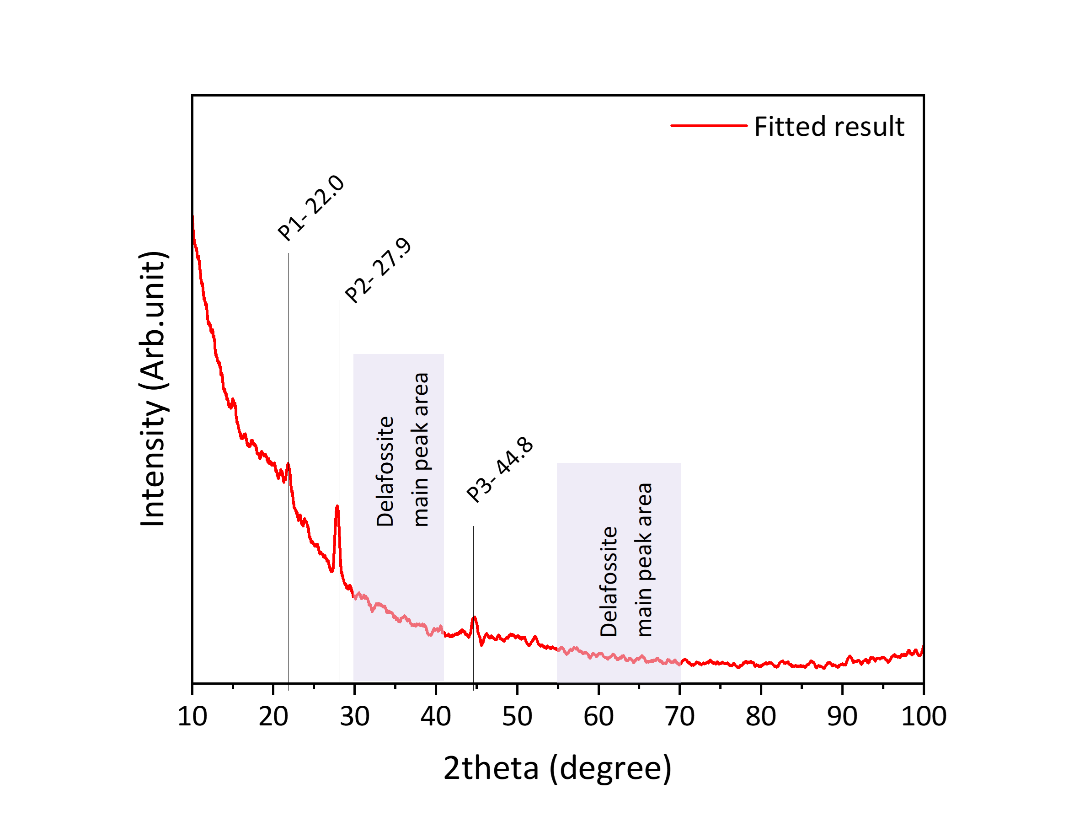


**Figure S3.** The crystal structure of the CVO NDs by XRD analysis.


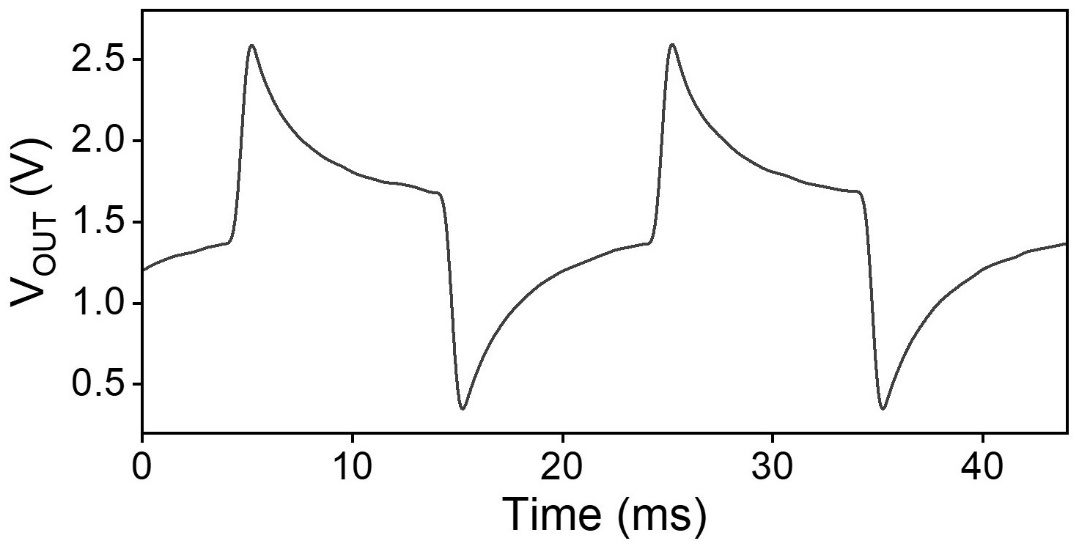


**Figure S4.** Photoresponse of the device under periodic light stimulation with 20 ms on/off cycles.


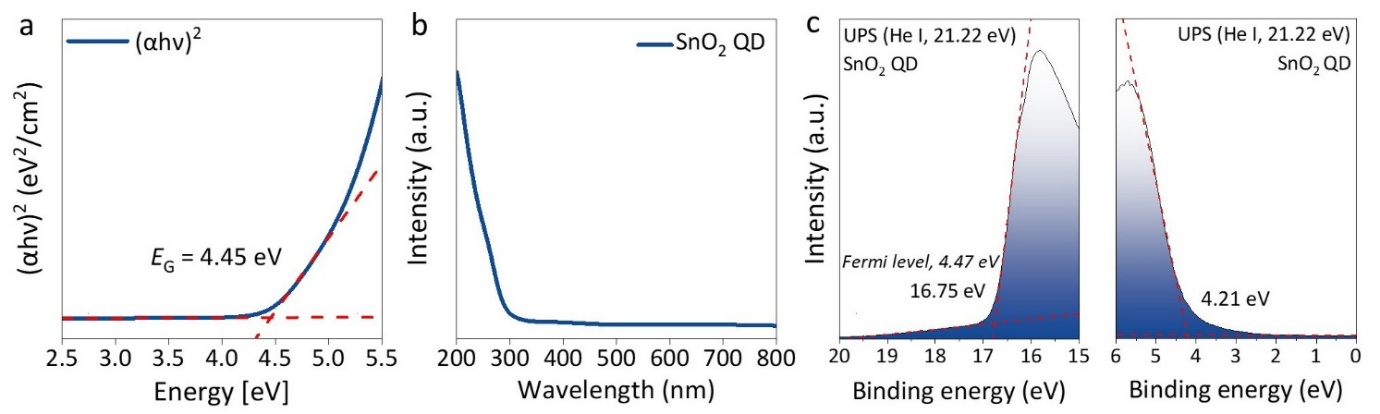


**Figure S5.** Optical and electronic structure characterization of SnO_2_ QD. (a) Tauc plot and (b) UV-Vis absorbance. (c) UPS analysis including the secondary electron cut-off region, used to determine the work function and valence band maximum.


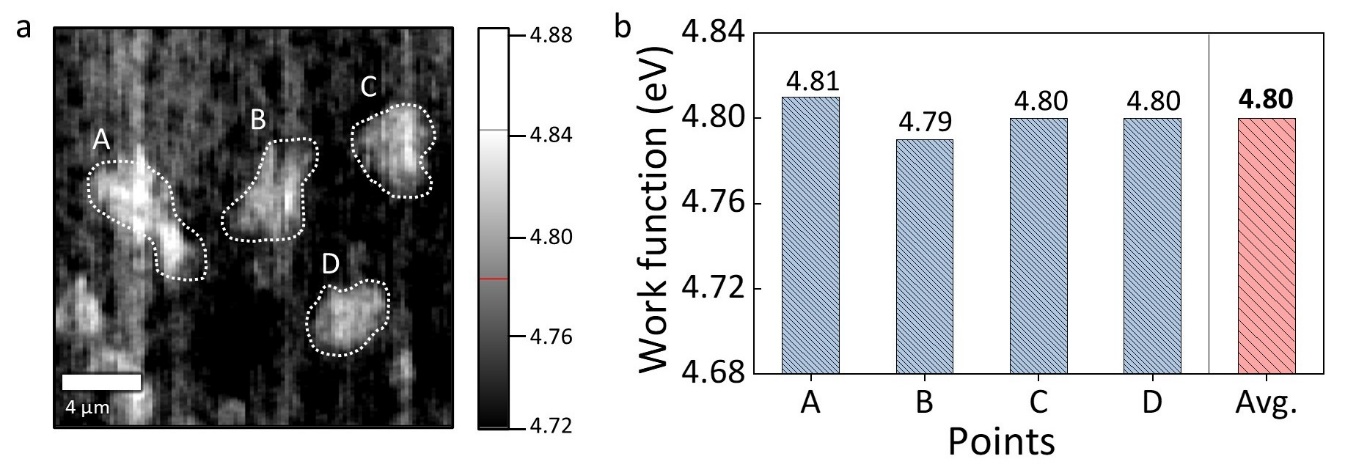


**Figure S6.** Surface potential analysis of CVO NDs using KPFM. (a) KPFM surface potential map of the CVO NDs. (b) Extracted work function values and average potential measured at four distinct regions (Points A–D).


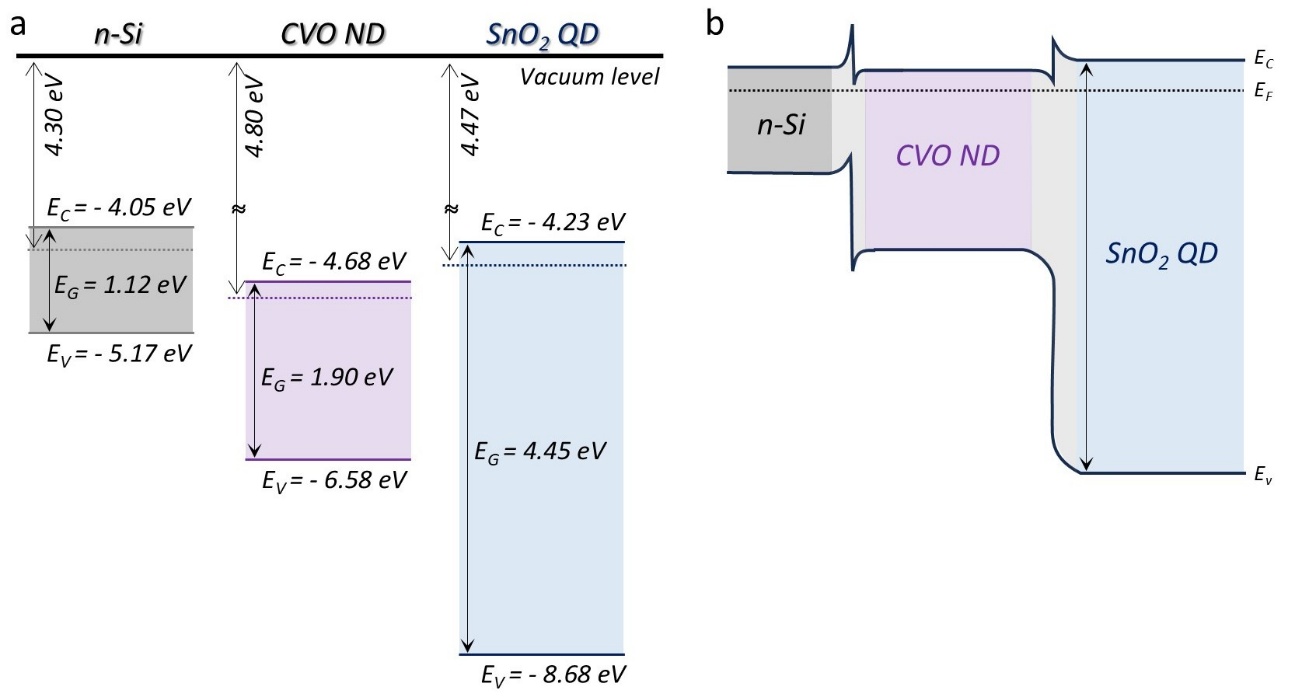


**Figure S7.** Energy Band diagram of PS-TRNG device. (a) Energy levels of the constituent materials: *n*-Si, CVO ND, and SnO_2_ QD. (b) Energy band alignment and bending under equilibrium conditions.


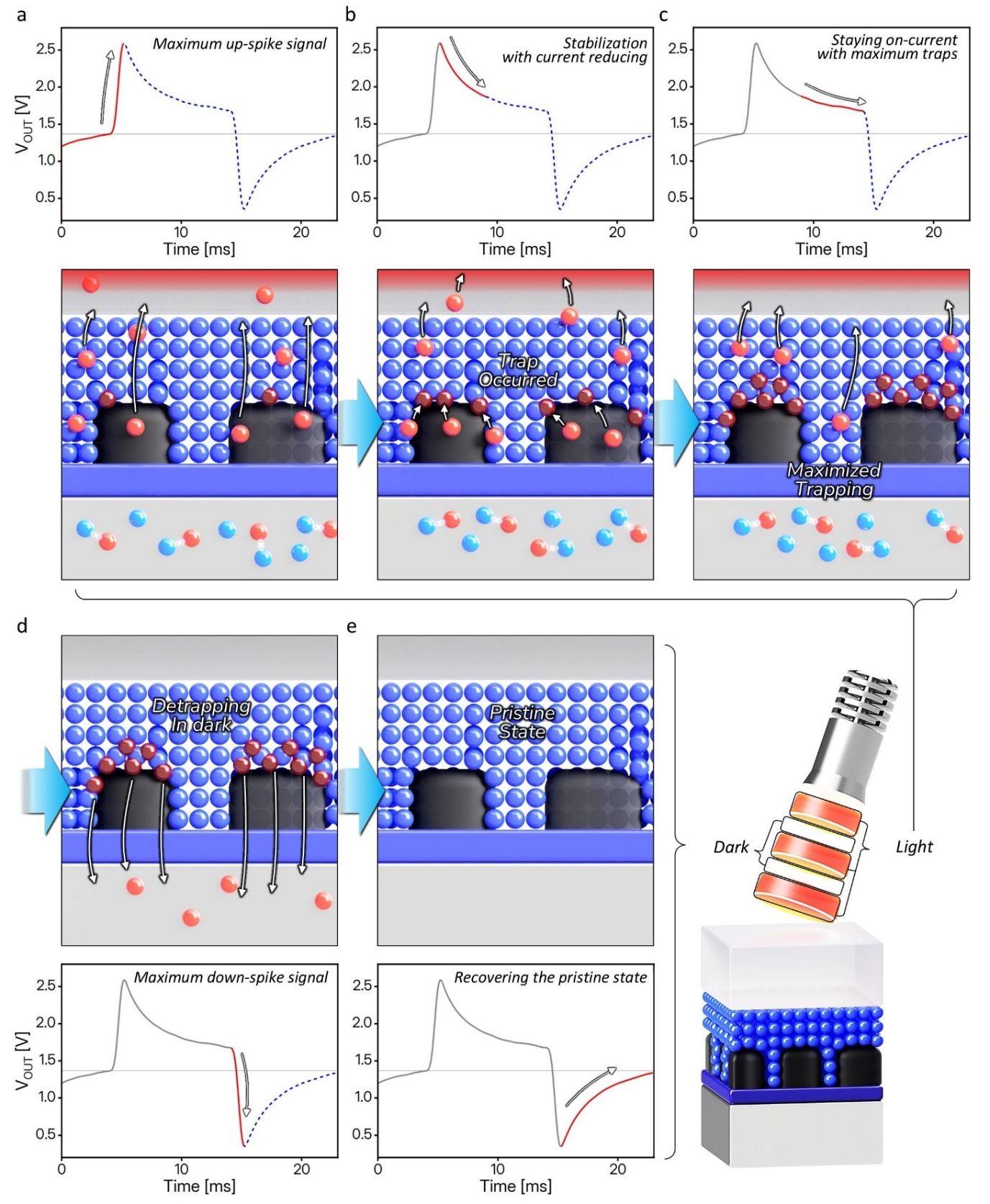


**Figure S8.** Schematic illustration of the photospike sequence in the PS-TRNG device. Time-resolved representation of carrier movement and corresponding voltage output during light switching events. (a–c) Carrier dynamics and voltage response upon light irradiation. (d, e) Behavior upon light turn-off, highlighting the relaxation process and resulting voltage decay.


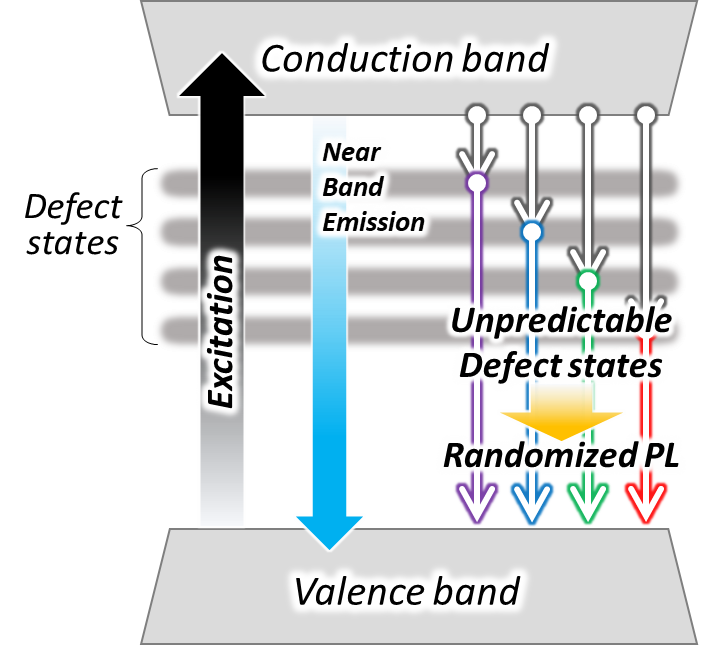


**Figure S9.** Schematic illustration of irregular photoluminescence peaks induced by defect states.


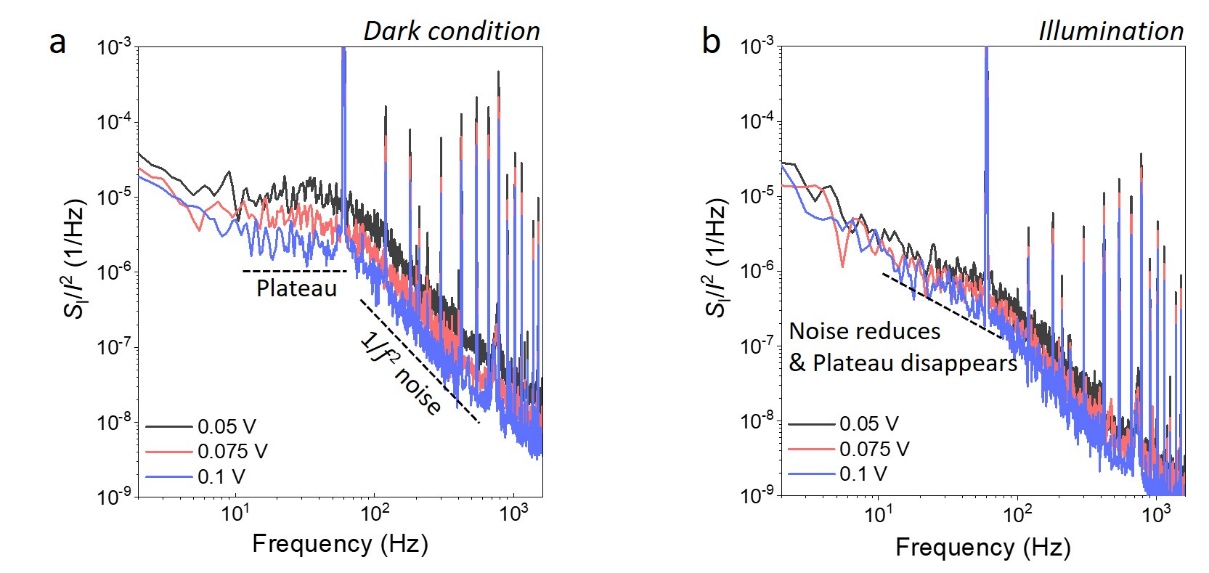


**Figure S10.** *S*_I_/*I*^2^ versus frequency of the two-terminal device under (a) dark and (b) illuminated (660 nm, 0.53 mW∙cm^-2^) conditions at applied voltages condition of 0.05, 0.075, and 0.1 V.


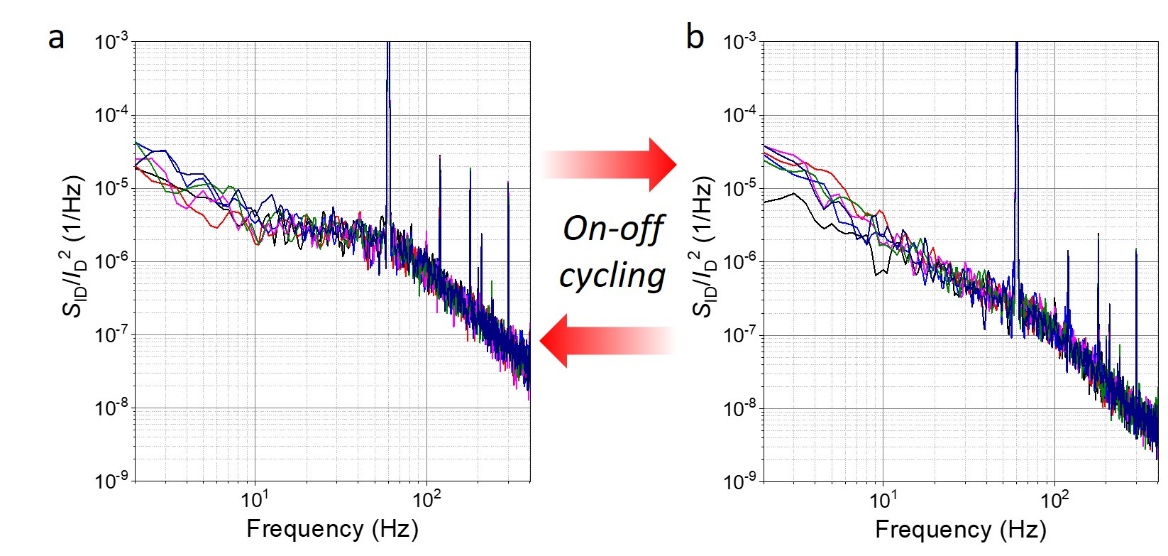


**Figure S11.** *S*_I_/*I*^2^ versus frequency of repeated on/off illumination measurements of the under a small DC bias (off: left, on: right).


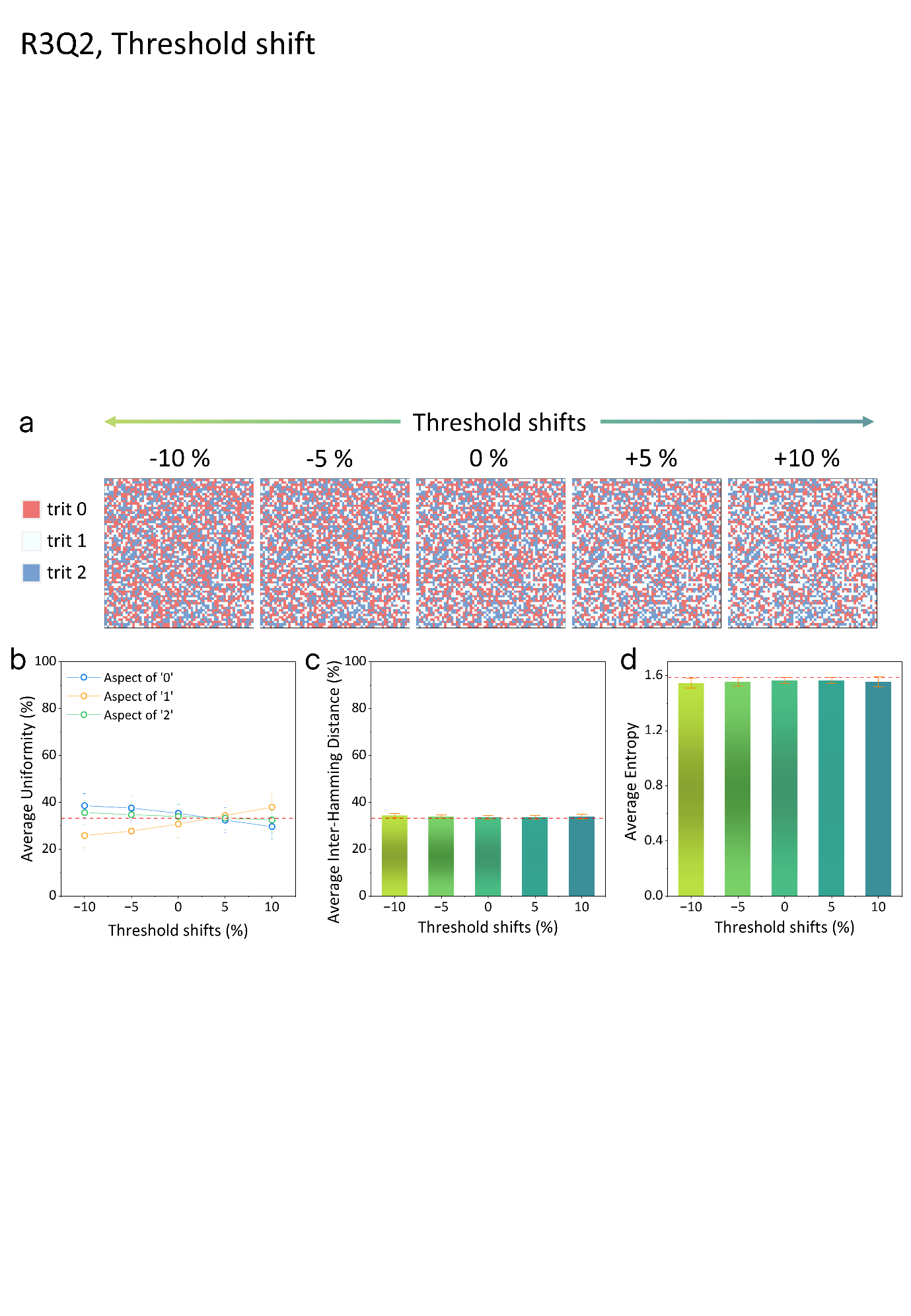


**Figure S12.** (a) Representative 64 × 64 ternary maps generated under threshold shifts of −10%, −5%, 0%, +5%, and +10%. (b) Average uniformity values for trit ‘0’, trit ‘1’, and trit ‘2’ as a function of the applied threshold shifts. The dashed red line indicates the statistical ideal value. (c) Average inter-HD across the threshold variations, and (d) average entropy calculated for each threshold condition. Error bars represent the standard deviation across rows for each metric (‘0’, ‘1’, and ‘2’).


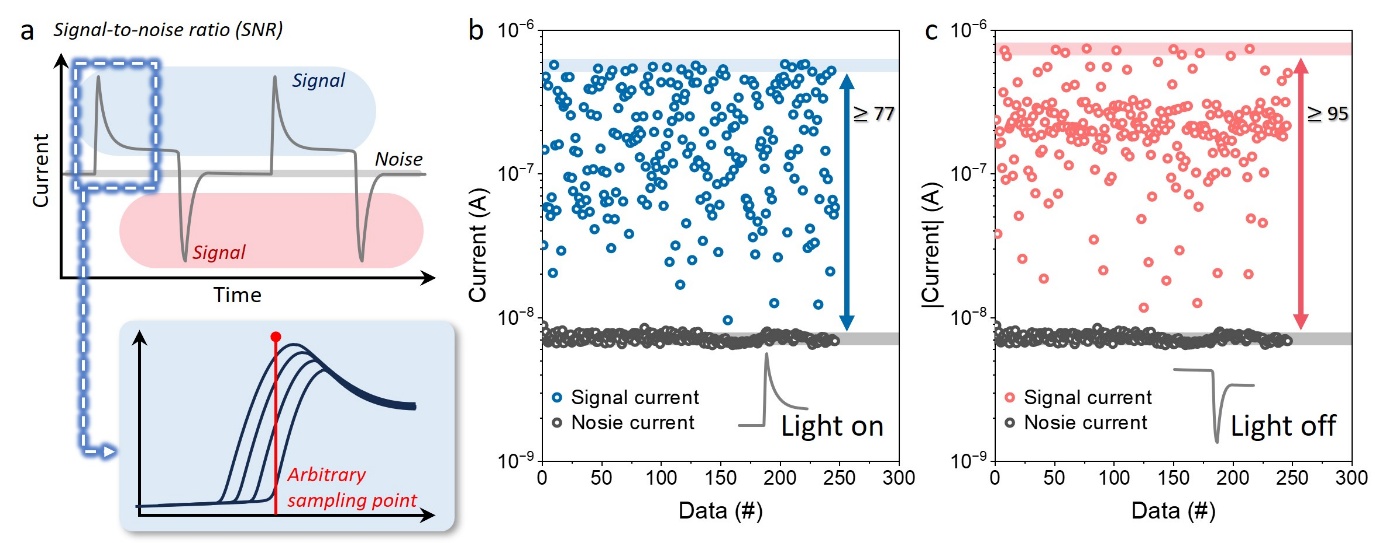


**Figure S13.** (a) Schematic illustration of the SNR measurement, showing spiking peaks and the corresponding sampling points used for random numbers. Measured signal and noise currents under (b) light-on and (c) light-off conditions (under 660 nm illumination with an optical power density of 0.53 mW·cm^-2^).


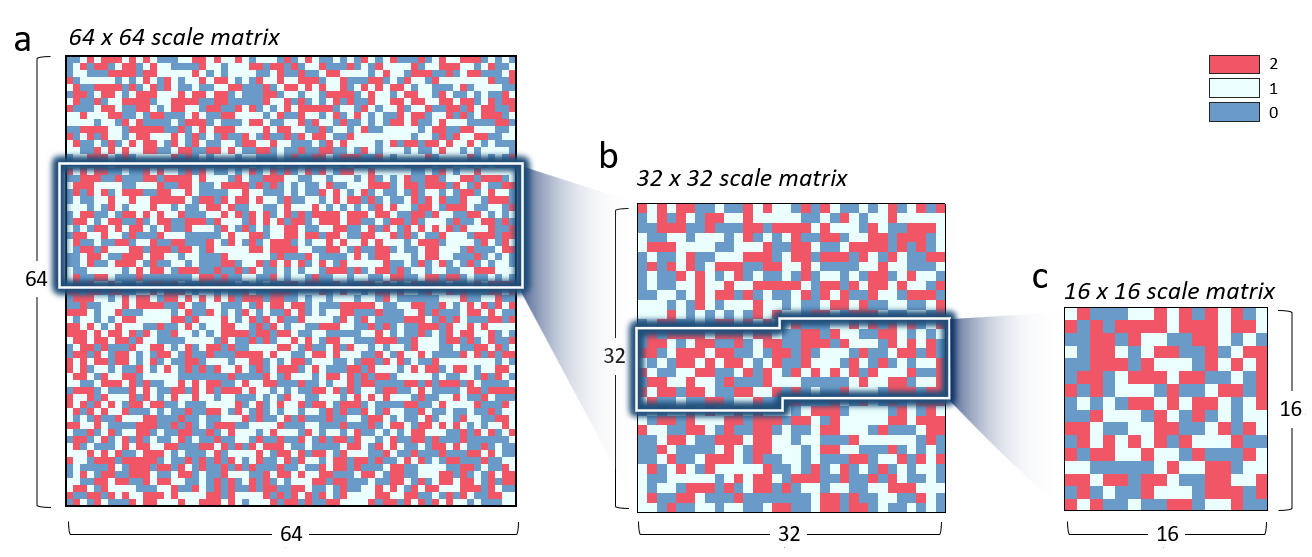


**Figure S14.** Schematic illustration of the iterative downscaling process showing (a) 64 × 64 matrix, (b) 32 × 32 matrix, and (c) 16 × 16 matrix.


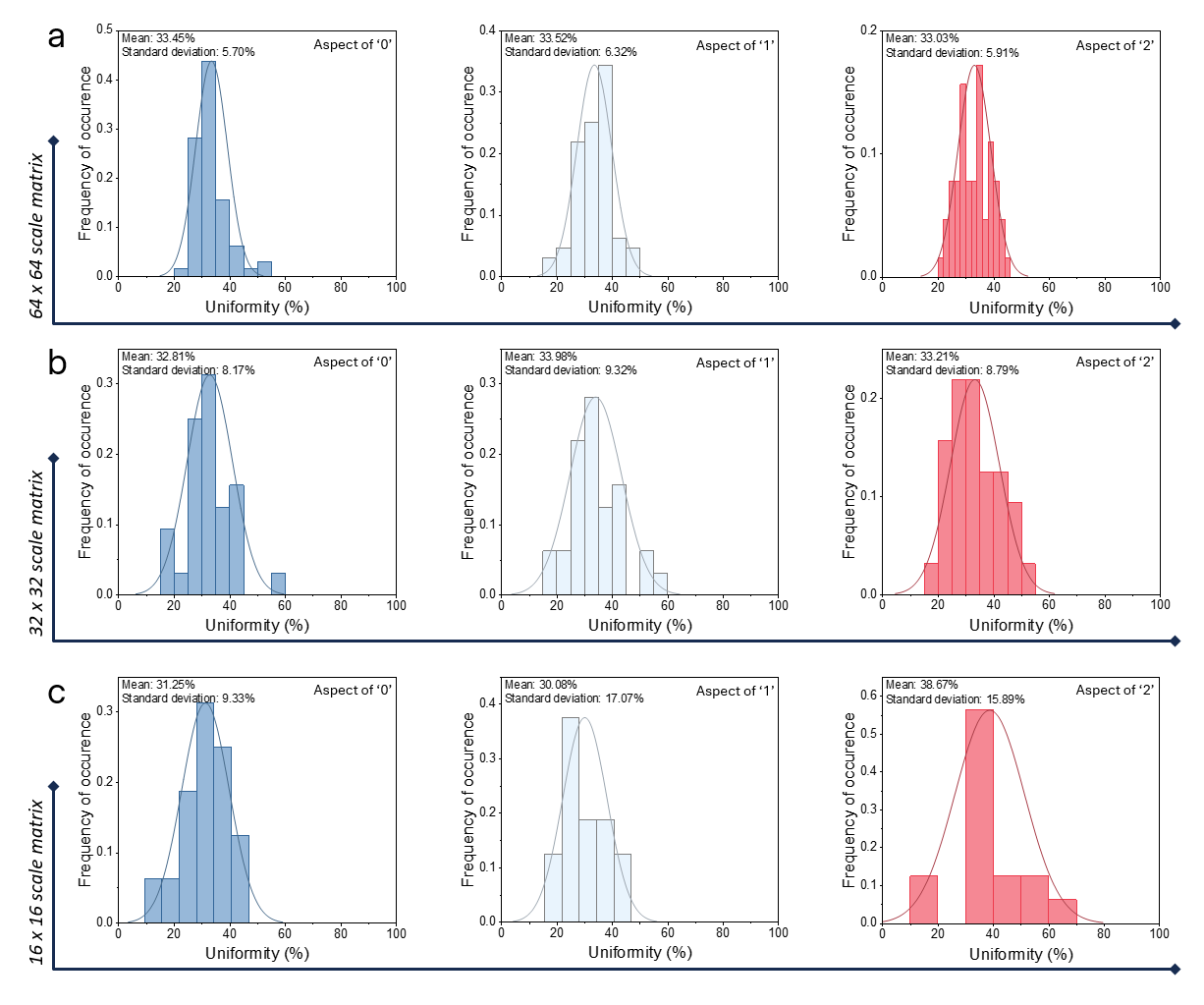


**Figure S15.** Uniformity analyses for the (a) 64 × 64, (b) 32 × 32, and (c) 16 × 16 matrices.

**
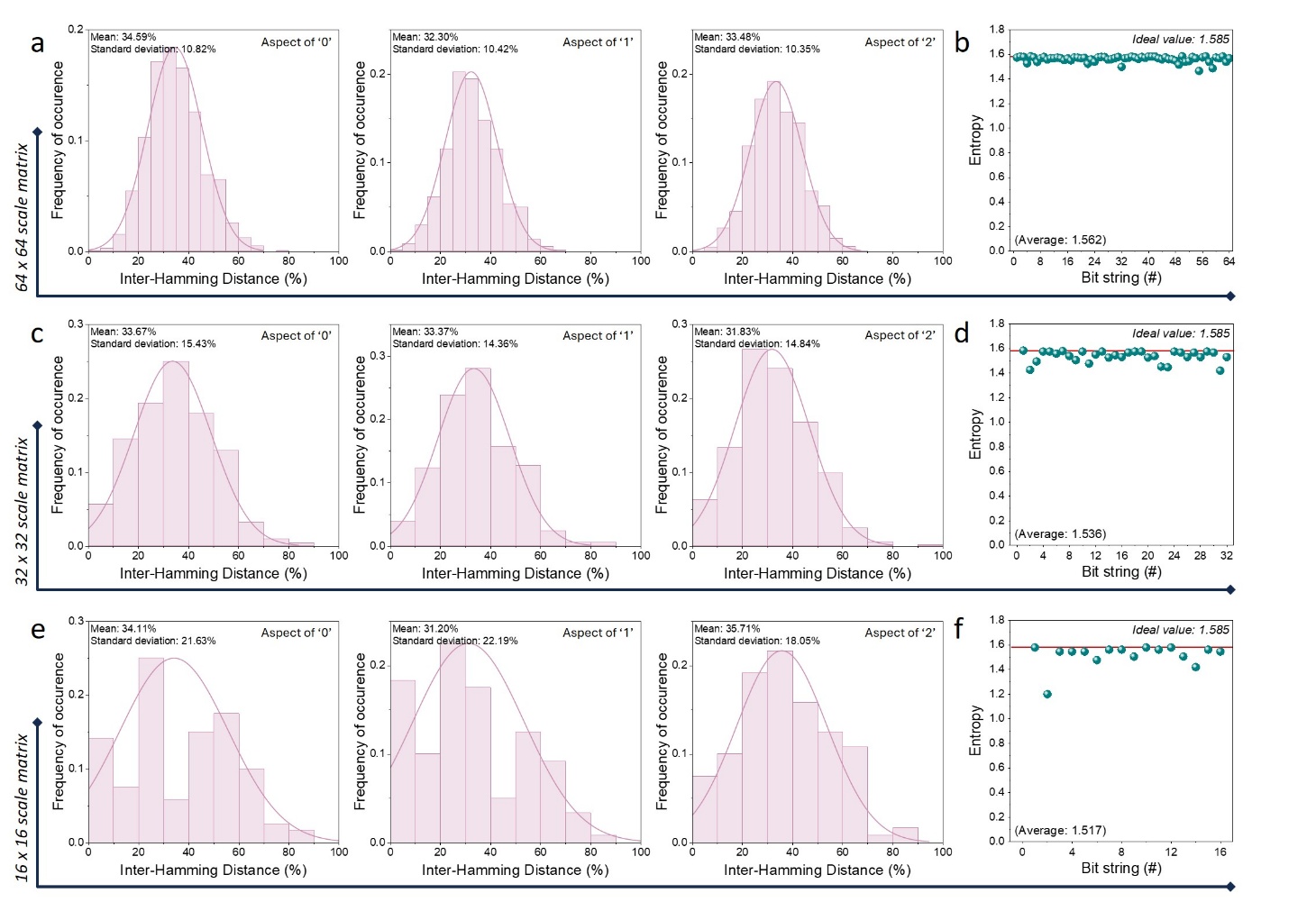
**

**Figure S16.** Inter-HD and entropy analyses of the PS-TRNG at different matrix scales. Inter-HD distributions of ternary outputs for the (a) 64 × 64, (b) 32 × 32, and (c) 16 × 16 matrices. (d–f) Corresponding entropy evaluations for each matrix scale.


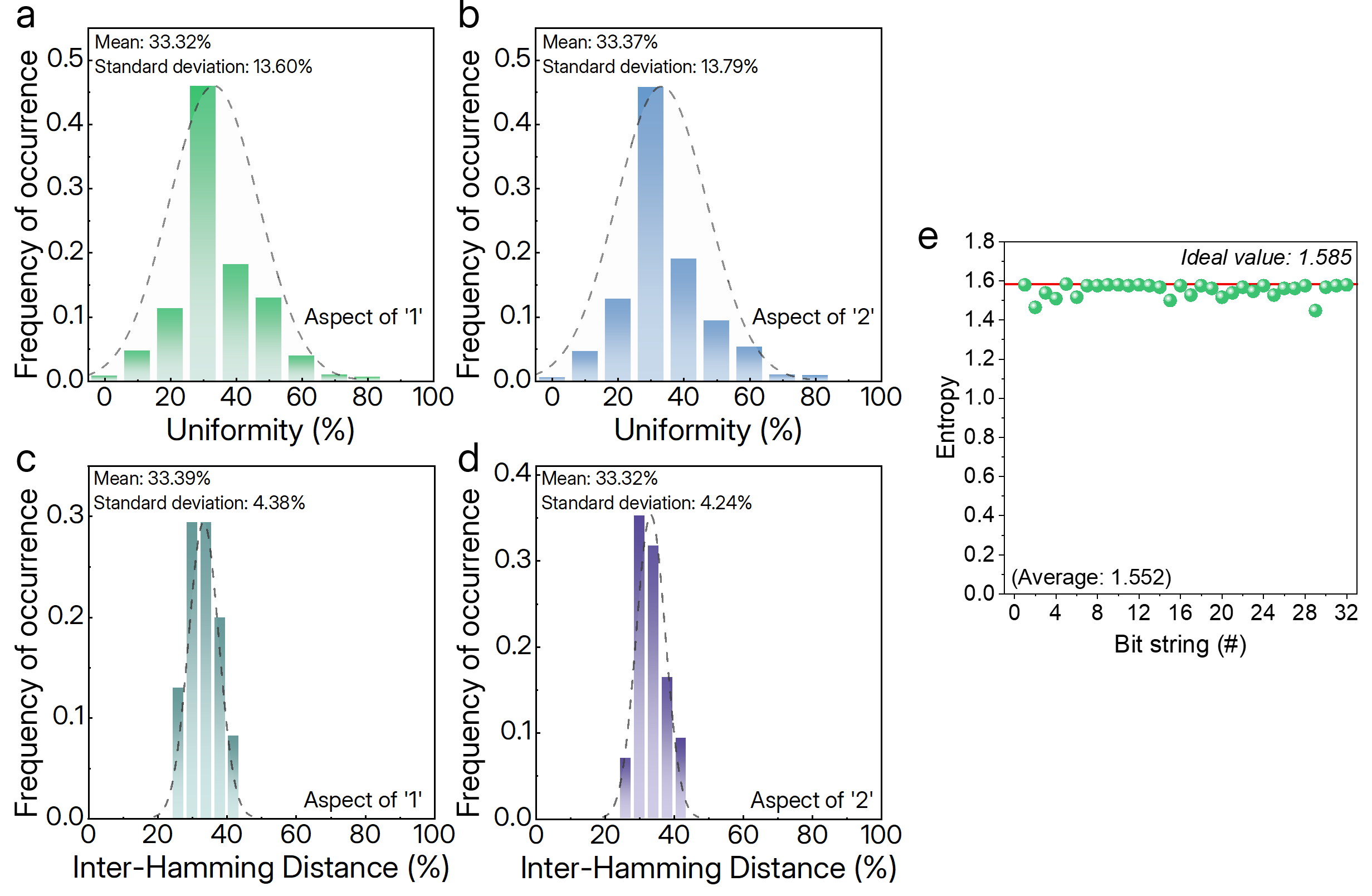


**Figure S17.** Detailed evaluation of the randomness characteristics of the generated trits. (a, b) Uniformity evaluation illustrating the distribution balance among trit values. (c, d) Inter-HD analysis performed separately for the ‘1’ and ‘2’ trits, highlighting the statistical independence across successive outputs. (e) Extracted ternary entropy of the generated trit sequence.


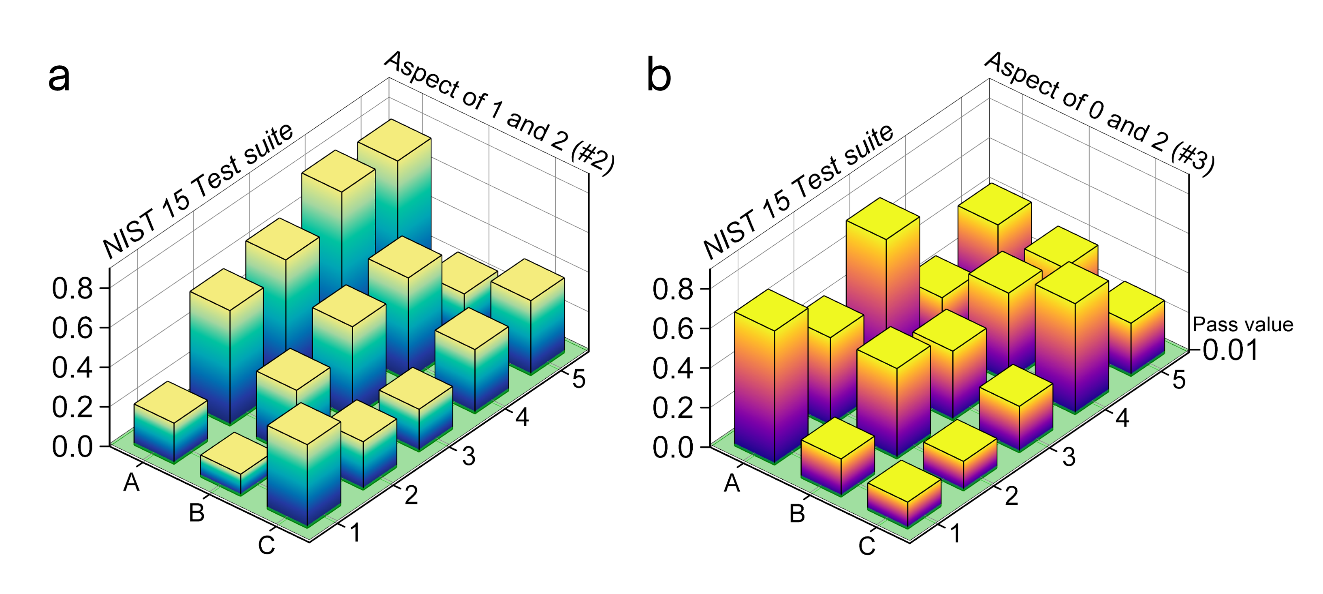


**Figure S18.** NIST statistical test results for the proposed ternary random numbers. (a) Test results of the PS-TRNG evaluated with respect to aspects 1 and 2. (b) Corresponding results evaluated with respect to aspects 0 and 2.


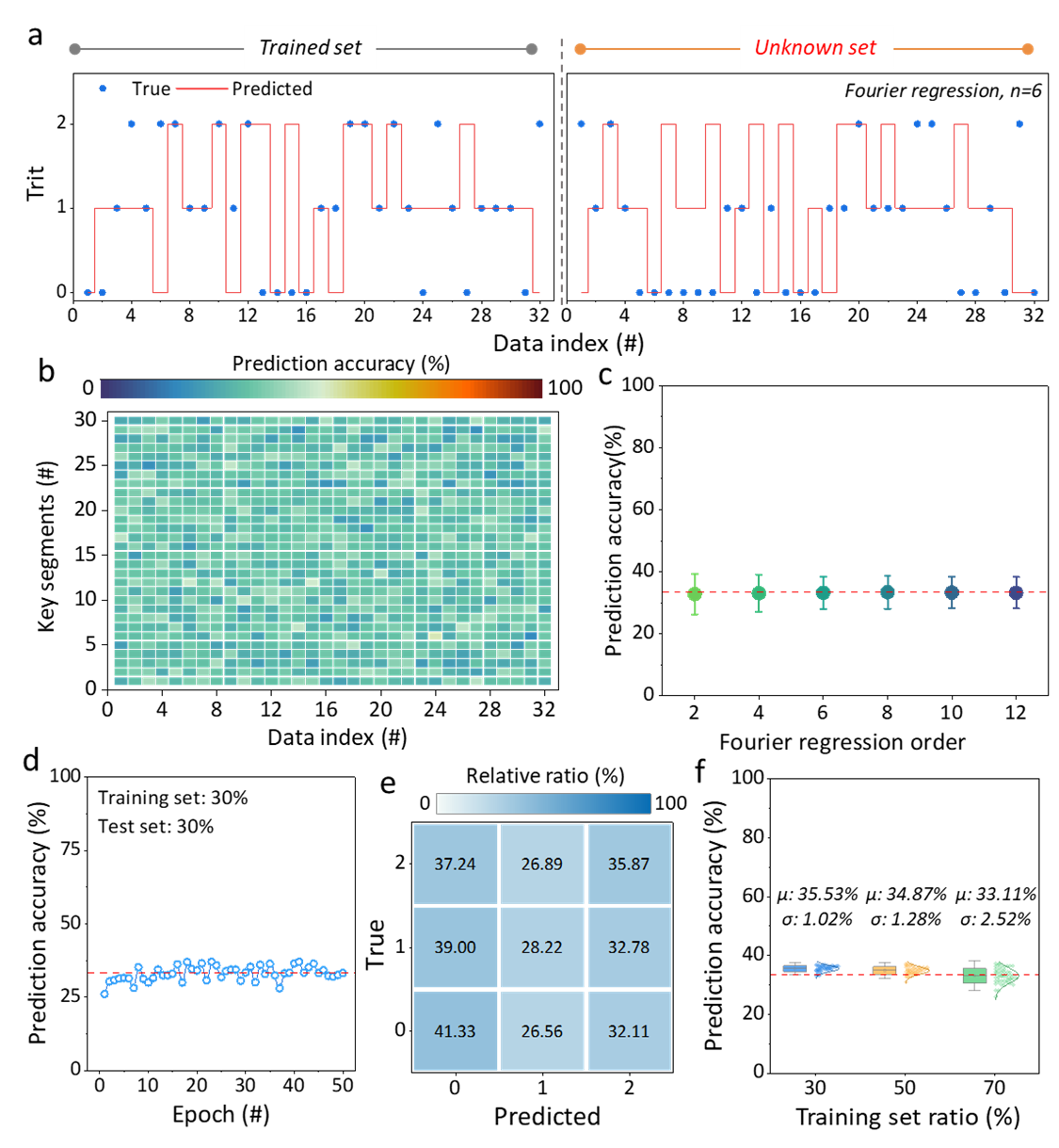


**Figure S19.** Machine learning based modeling attack analysis of the PS-TRNG output. (a) Fourier-basis regression analysis for a 32-trit key segment. The model partially fits the trained portion of the sequence (left) but fails to reproduce the unseen sequence (right), with prediction accuracy dropping to the random-guessing level for ternary outputs. (b) Prediction accuracy summarized over all key segments and trit indices. (c) Averaged prediction accuracy for Fourier regression orders ranging from n = 2 to n = 12, all remaining within the statistical fluctuation of random guessing. (d) Training curves of the LSTM-based recurrent neural network, demonstrating rapid convergence without meaningful improvement in predictability. (e) Confusion matrix of LSTM predictions, showing nearly uniform class occurrence for trits ‘0’, ‘1’, and ‘2’. (f) Test accuracy across different training ratios (30%, 50%, 70%), all remaining in the range of 33–36%, confirming the absence of temporal correlation and validating the TRNG’s robustness against neural-network–based modeling attacks.


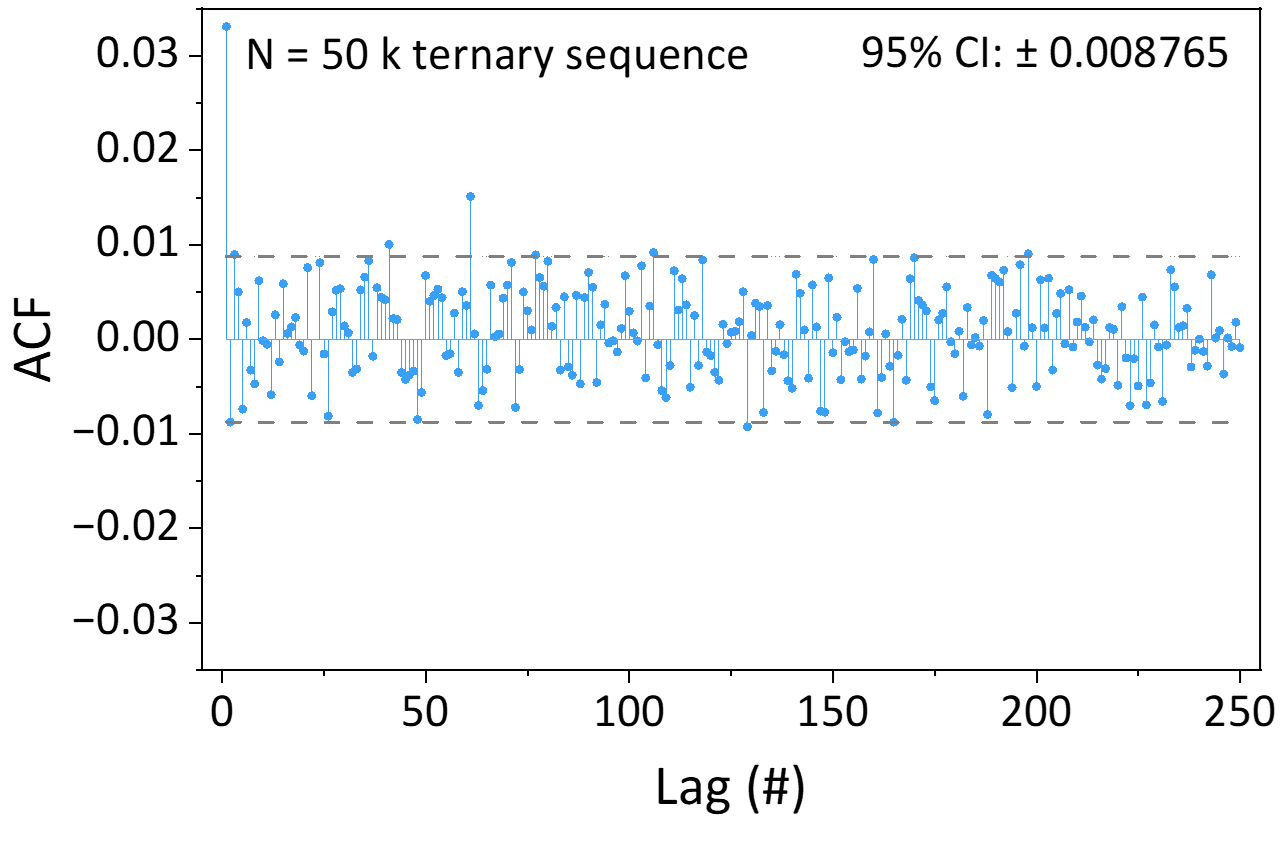


**Figure S20.** ACF of the generated ternary random sequence, showing symmetric fluctuations around zero without distinct periodicity, confirming the absence of temporal correlation.


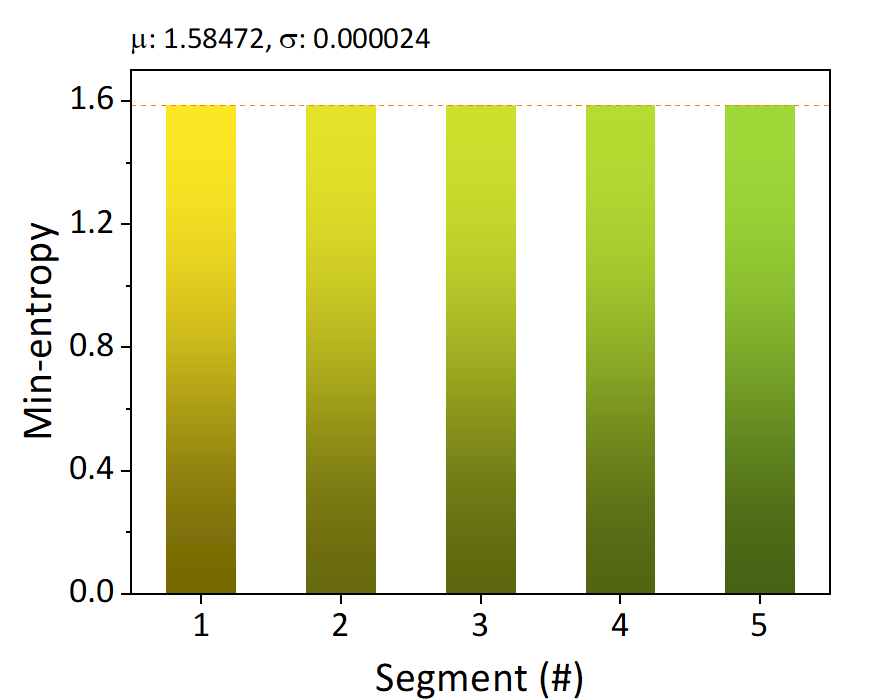


**Figure S21.** Minimum-entropy values of 5 non-overlapping 100-k-trit segments evaluated using the Most Common Value estimator defined in NIST SP 800-90B. Each bar represents the per-trit minimum entropy.


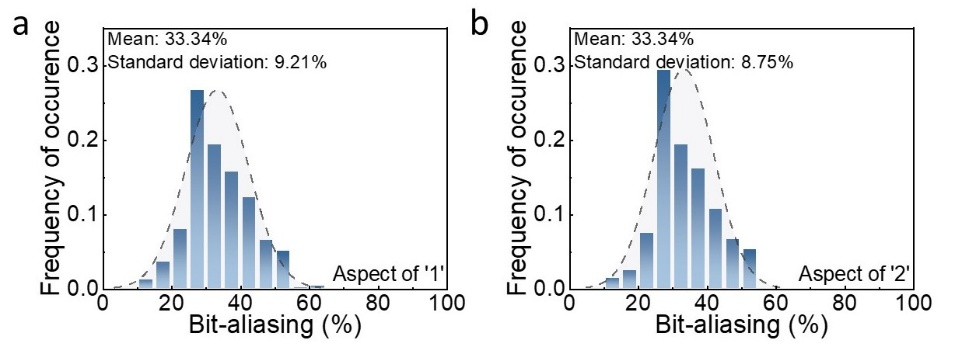


**Figure S22.** Validation of bit-aliasing between parallel output data sets. (a) Bit-aliasing analysis focused on the ‘1’ trits. (b) Bit-aliasing analysis for the ‘2’ trits, demonstrating consistency in aliasing behavior across distinct trit values.


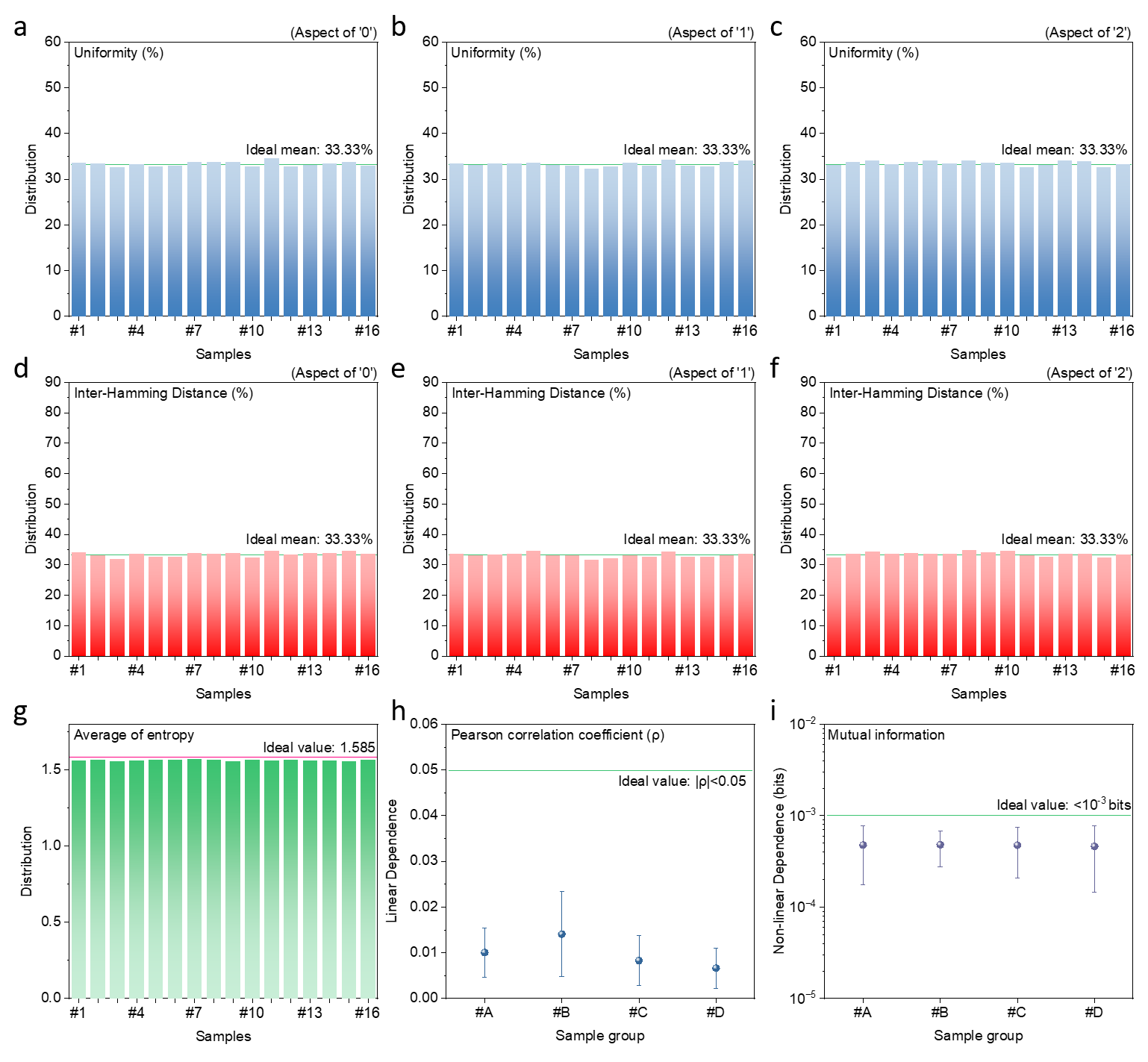


**Figure S23.** Statistical analysis of the random sequences generated from sixteen samples. Uniformity analysis of the generated ternary random numbers for trit values (a) ‘0’, (b) ‘1’, and (c) ‘2’. Inter-HD for trit values (d) ‘0’, (e) ‘1’, and (f) ‘2’. (g) Entropy of the generated ternary random numbers. (h) Pearson correlation coefficient and (i) mutual information calculated among the bitstreams within groups #A, #B, #C, and #D, confirming the absence of inter-sample dependency.


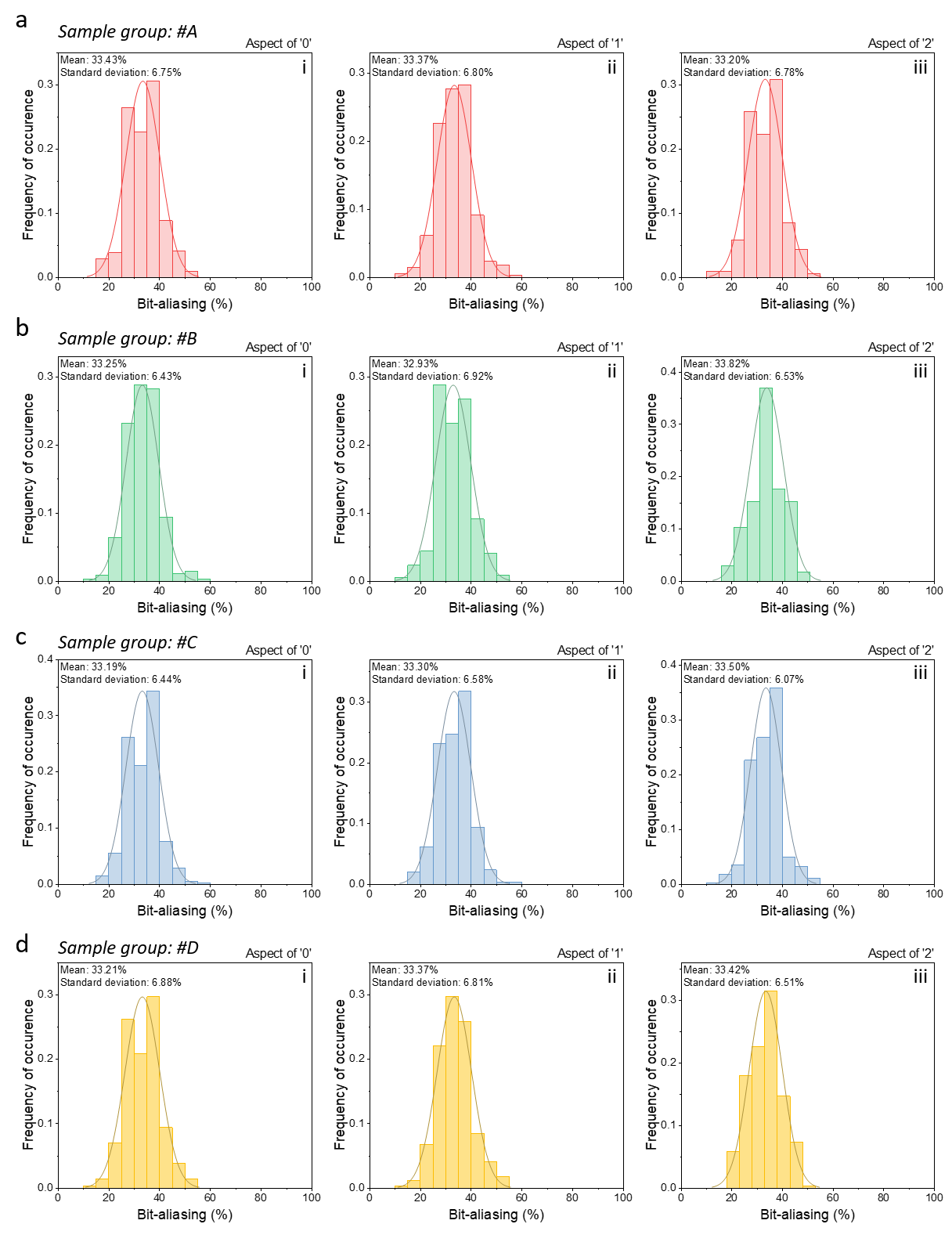


**Figure S24.** Bit-aliasing analysis performed within sample Groups (a) #A, (b) #B, (c) #C, and (d) #D, each consisting of four samples. The comparison is based on 4,096 data points per sample and shows the statistical relationship among the random bitstreams generated under identical illumination conditions.


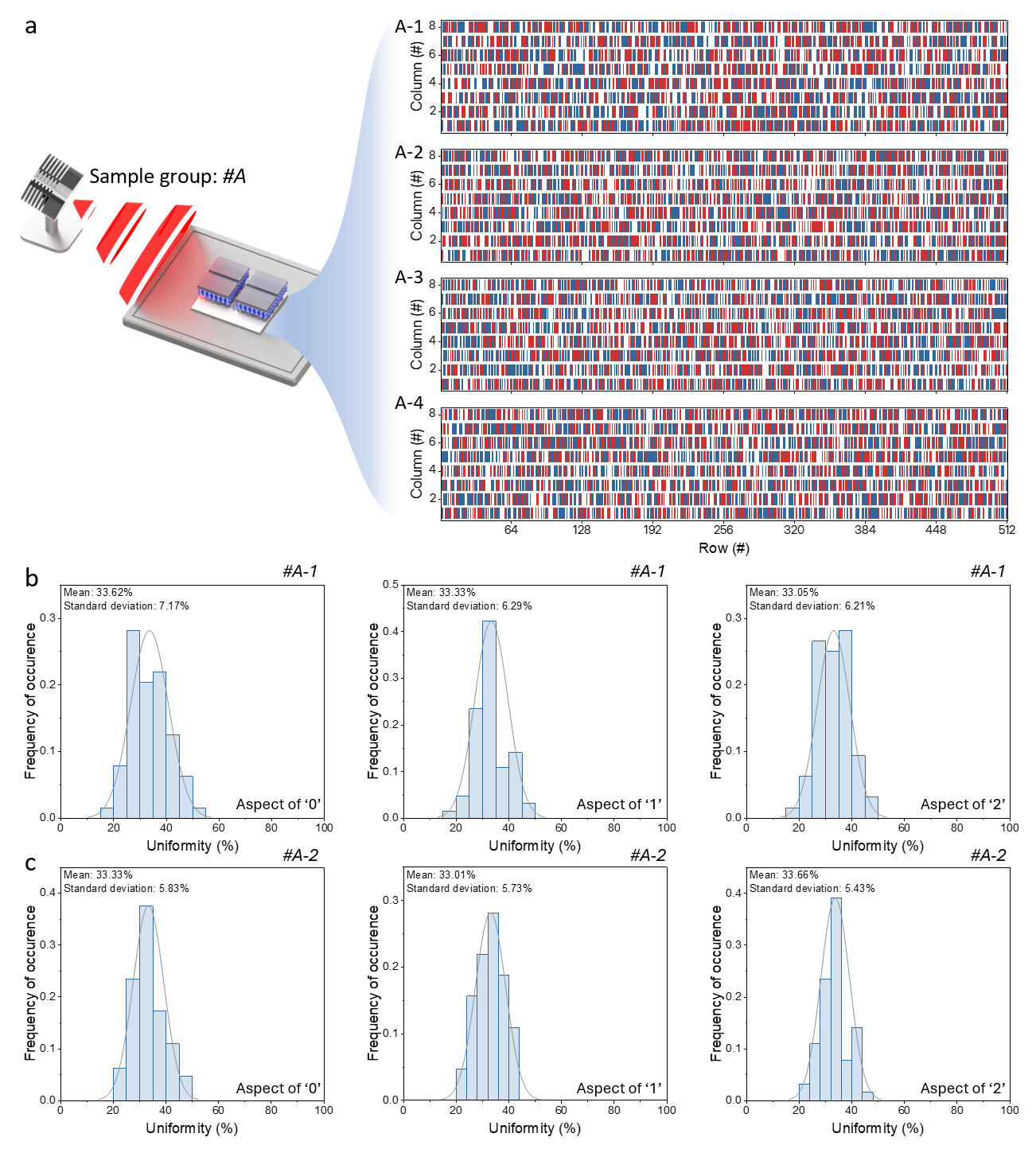


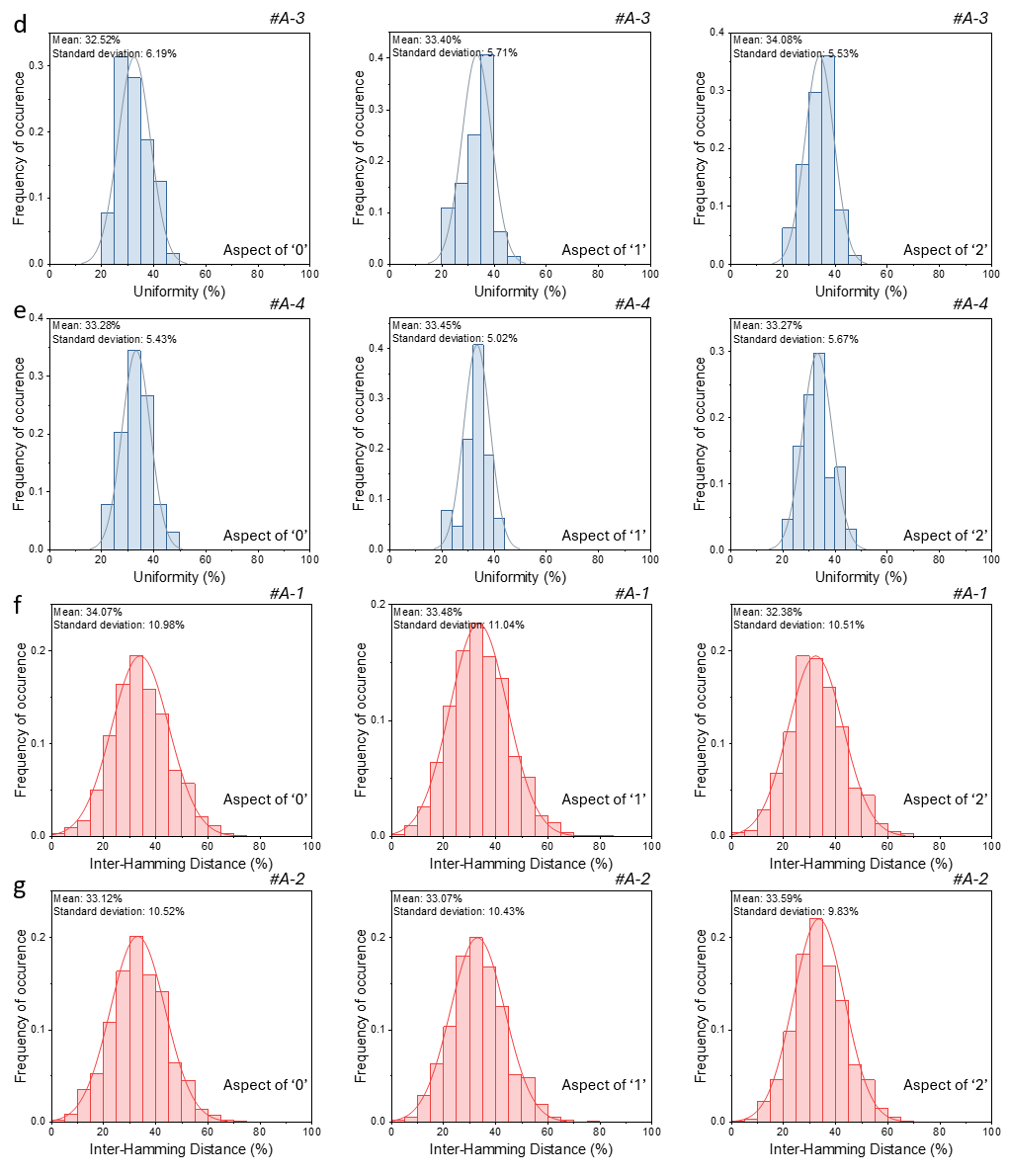


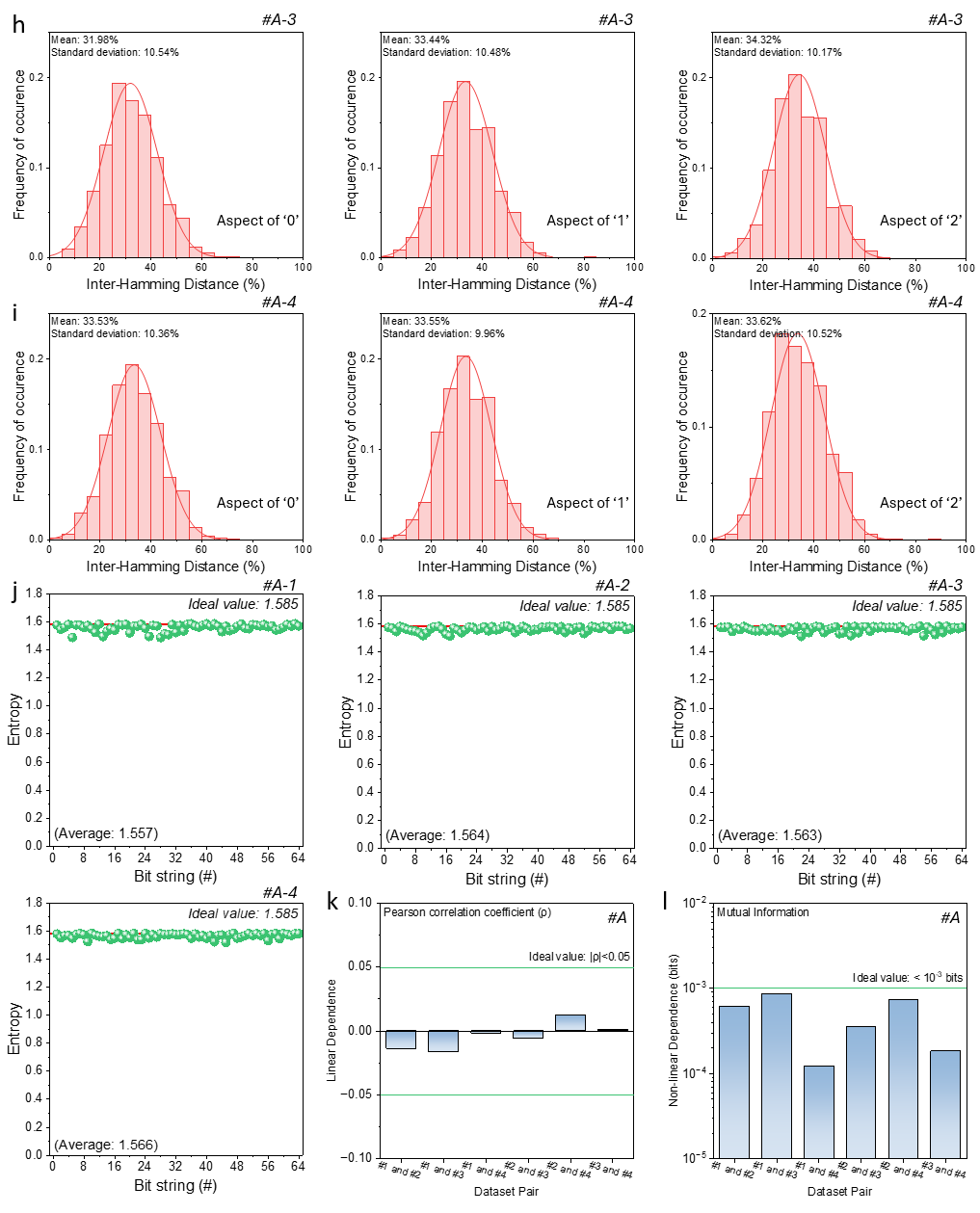


**Figure S25.** Comparison of the bitstreams generated simultaneously from the four samples within group #A. (a) Schematic of the measurement configuration for group #A and the corresponding 8 × 512 mapping. (b–e) Uniformity results for bitstreams A-1 to A-4. (f–i) Inter-HD analysis for bitstreams A-1 to A-4. (j) Entropy values extracted from bitstreams A-1 to A-4. (k) Pearson correlation coefficient and (l) mutual information results assessing the statistical dependence among the four bitstreams in group #A.


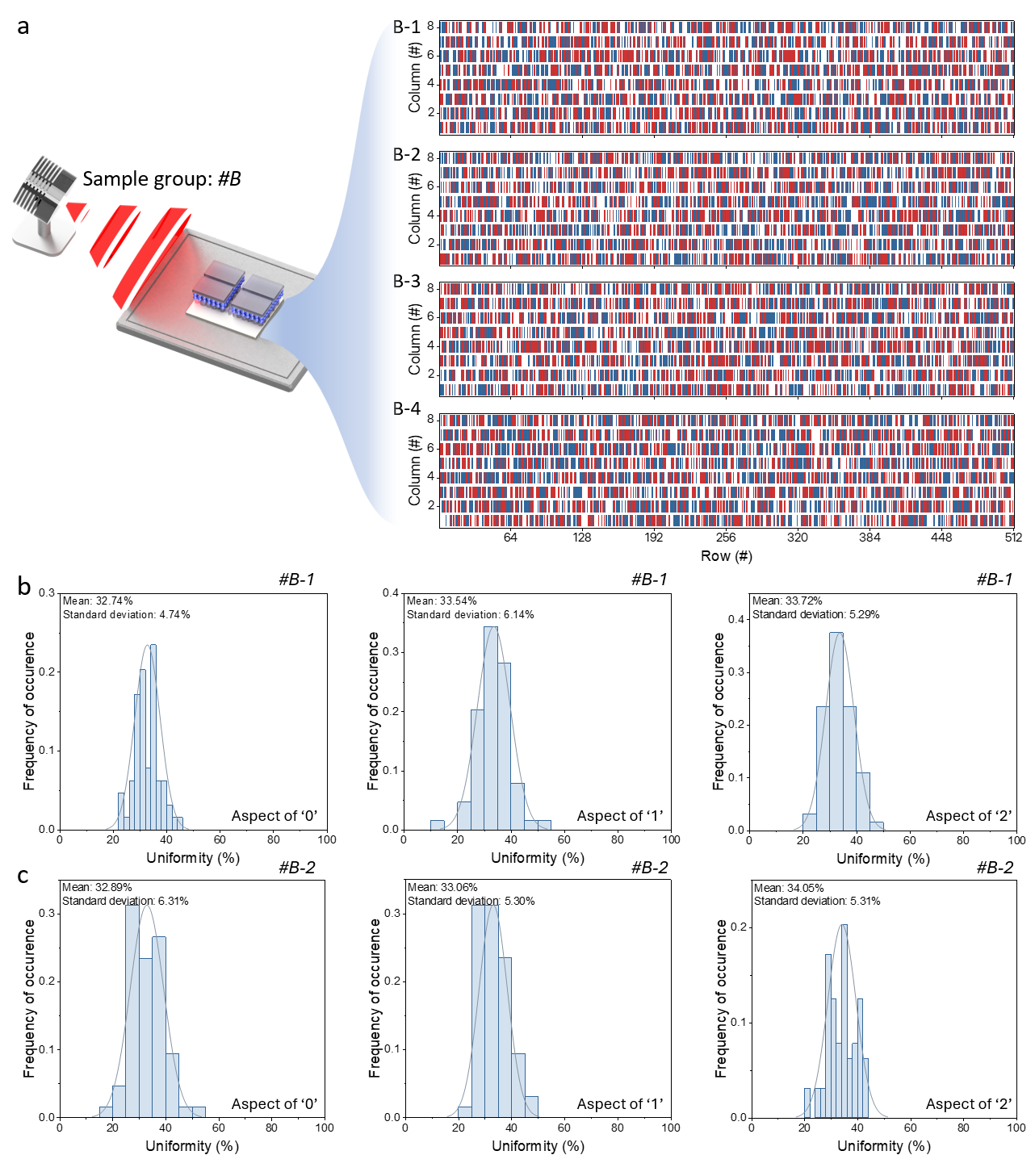


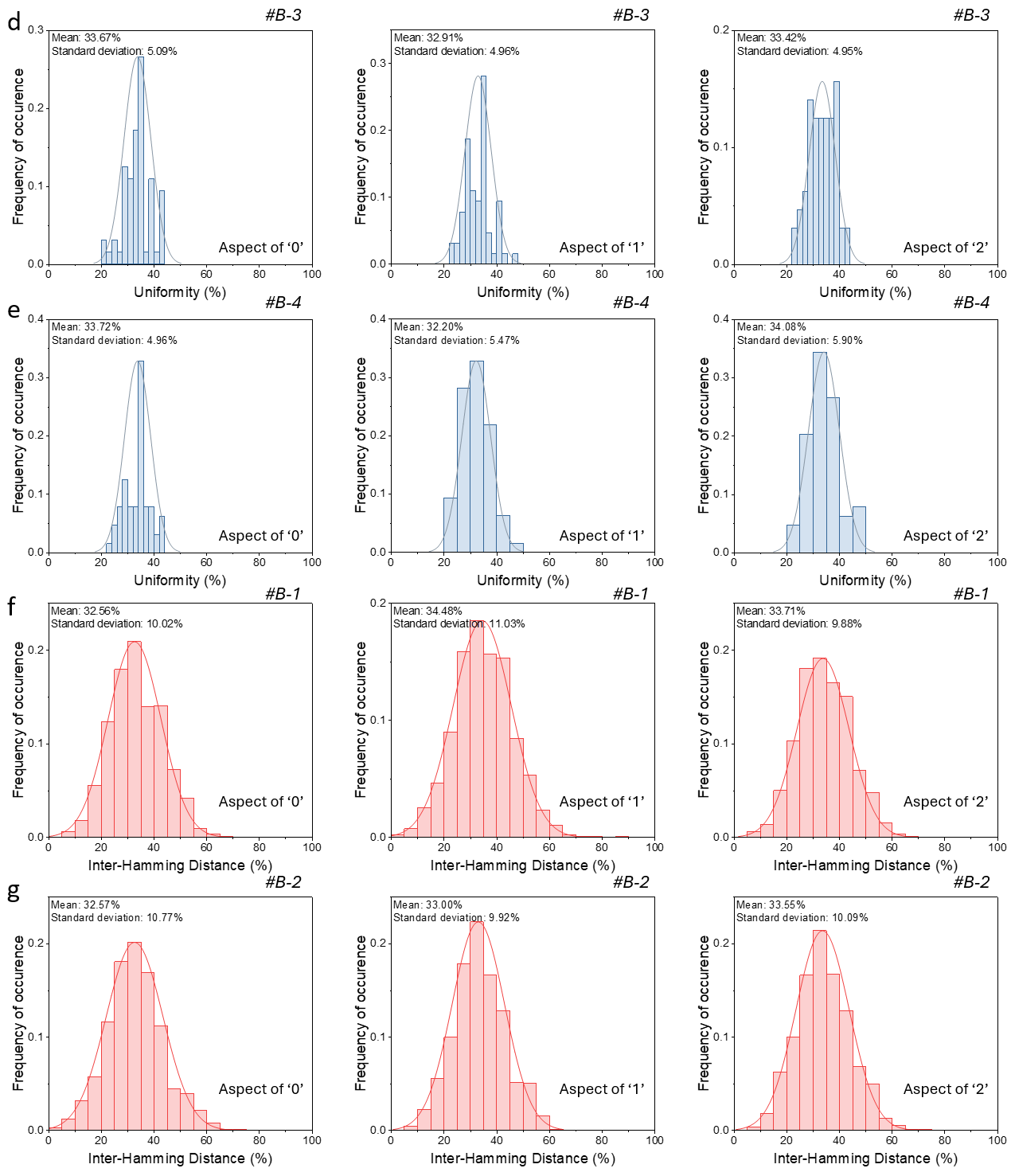


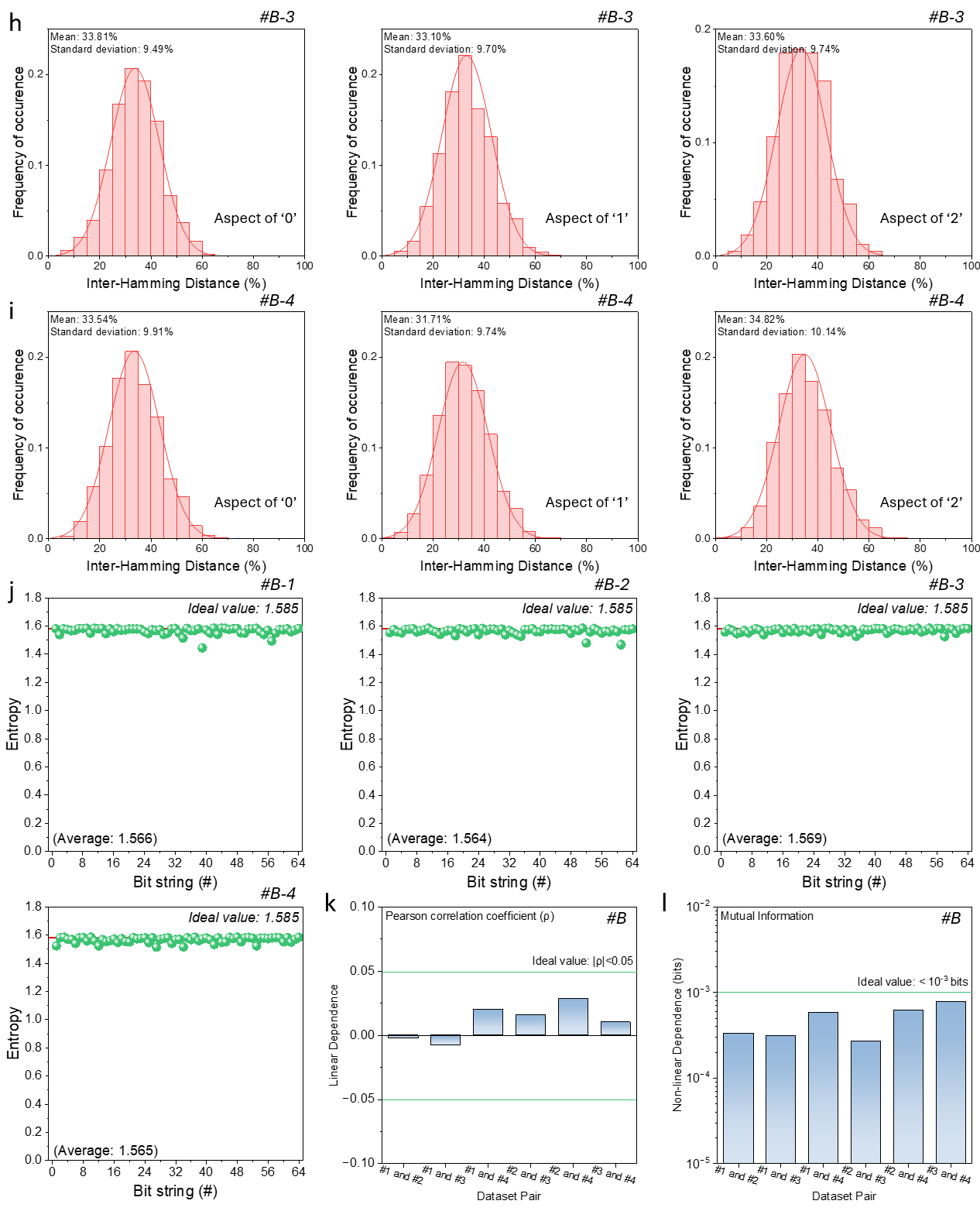


**Figure S26.** Comparison of the concurrently generated bitstreams from the four samples in group #B. (a) Measurement setup used for group #B along with the corresponding 8 × 512 ternary mapping. (b–e) Uniformity assessment for bitstreams B-1 to B-4. (f–i) Inter-HD profiles for bitstreams B-1 to B-4. (j) Entropy values derived from each bitstream. (k) Pearson correlation coefficient and (l) mutual information results quantifying the statistical relationship among the four bitstreams in group #B.


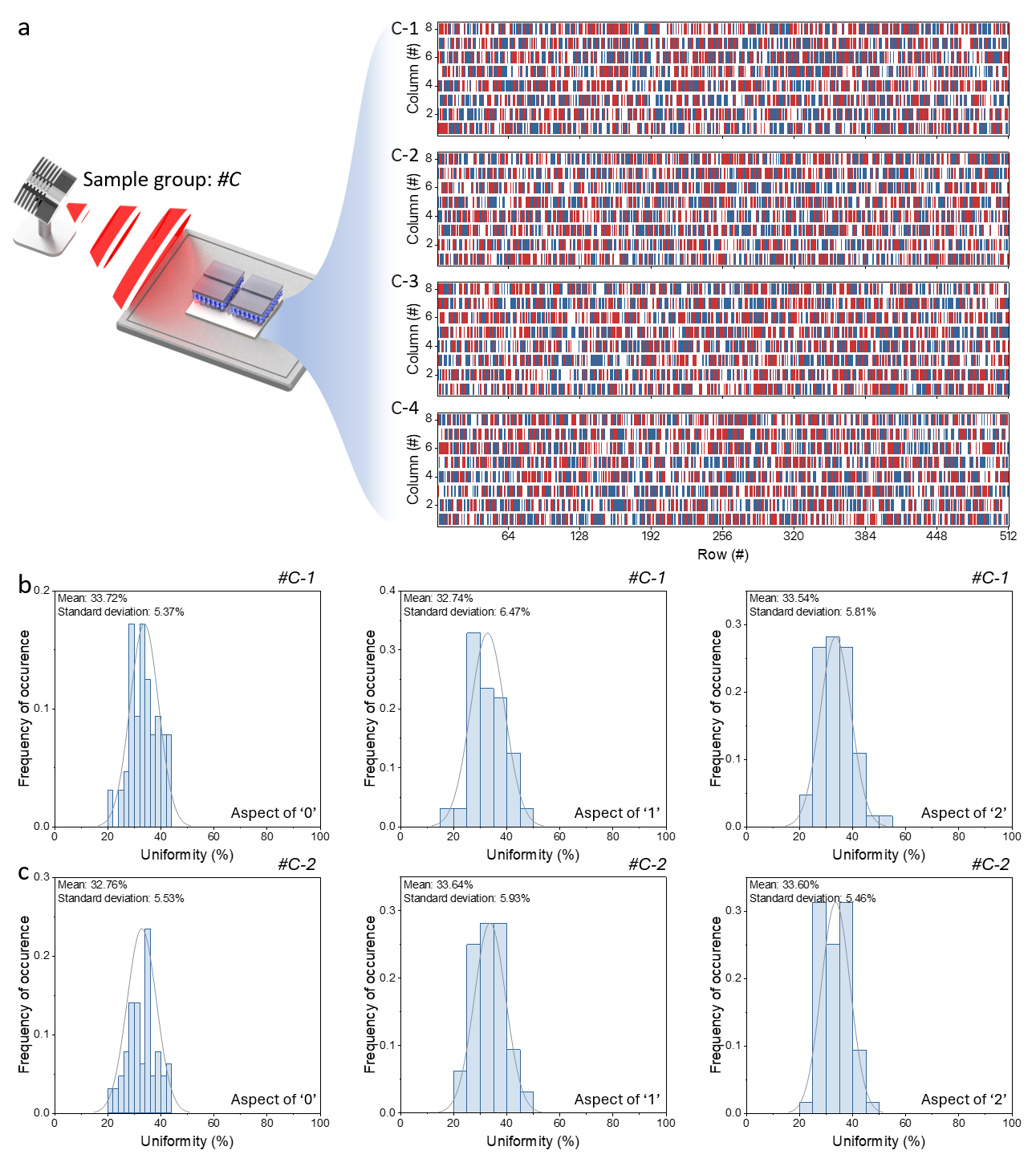


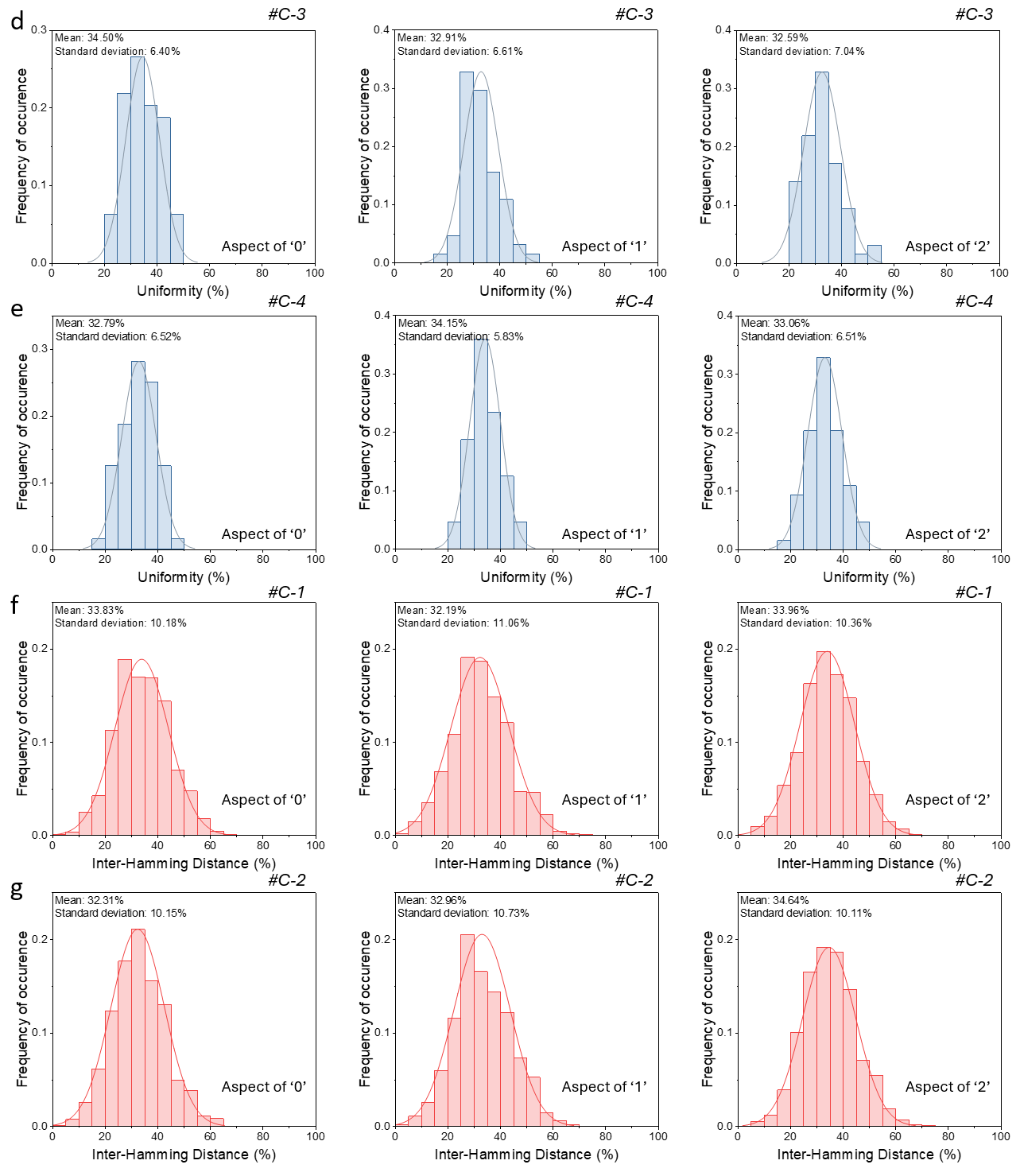


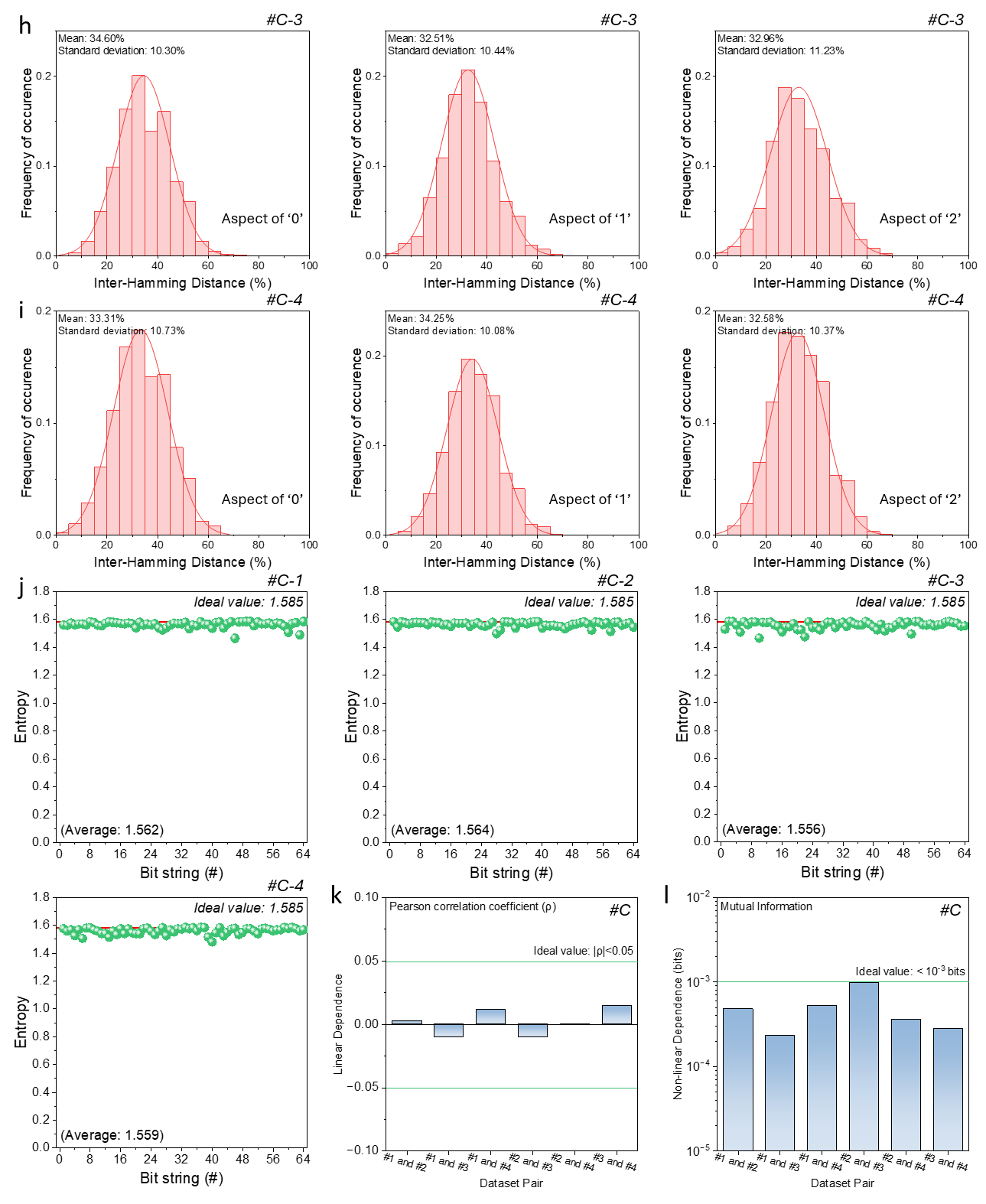


**Figure S27.** Comparison of the bitstreams generated simultaneously from the four samples within group #C. (a) Schematic of the measurement configuration for group #C and the corresponding 8 × 512 mapping. (b–e) Uniformity results for bitstreams C-1 to C-4. (f–i) Inter-HD analysis for bitstreams C-1 to C-4. (j) Entropy values extracted from bitstreams C-1 to C-4. (k) Pearson correlation coefficient and (l) mutual information results assessing the statistical dependence among the four bitstreams in group #C.


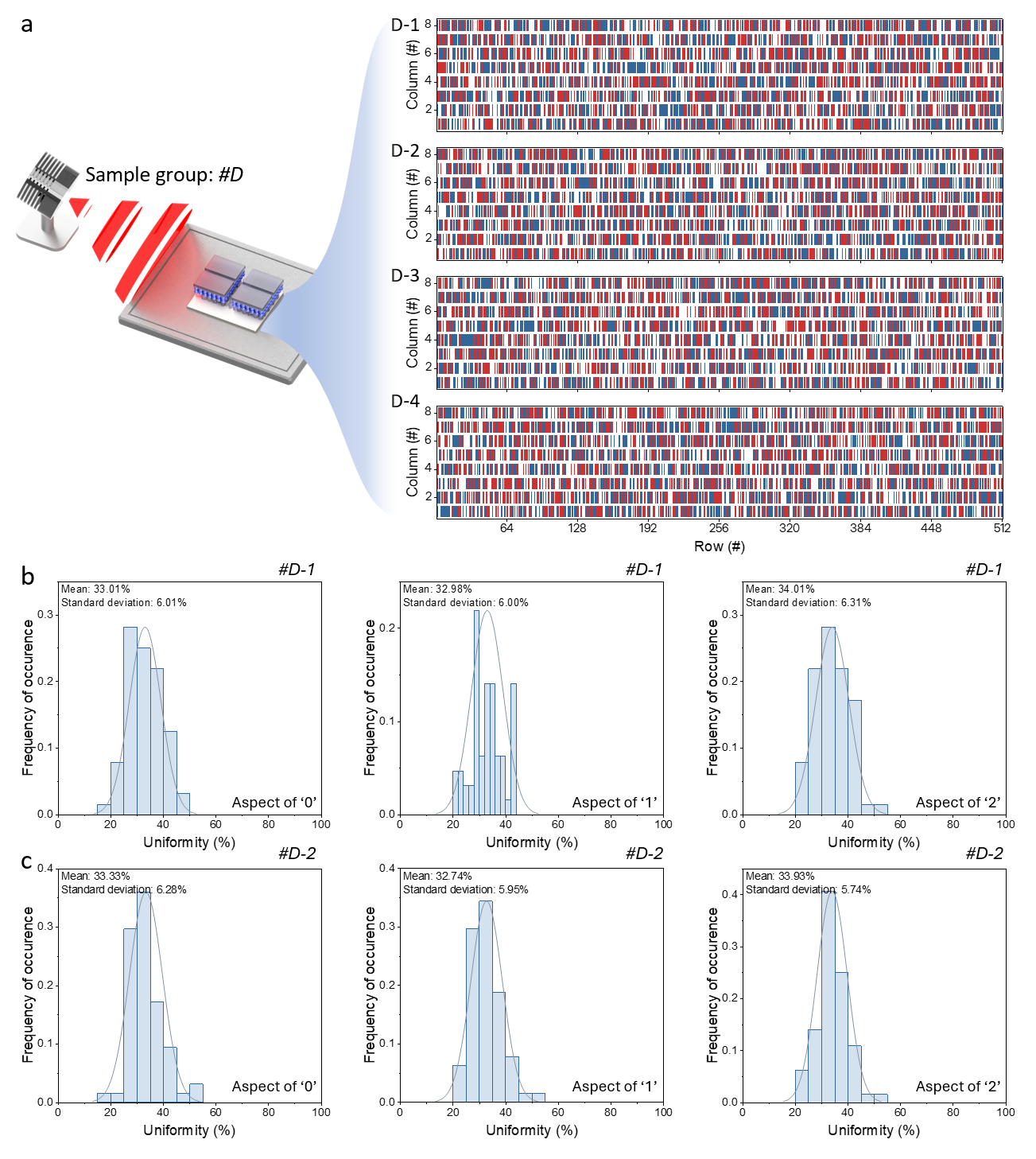


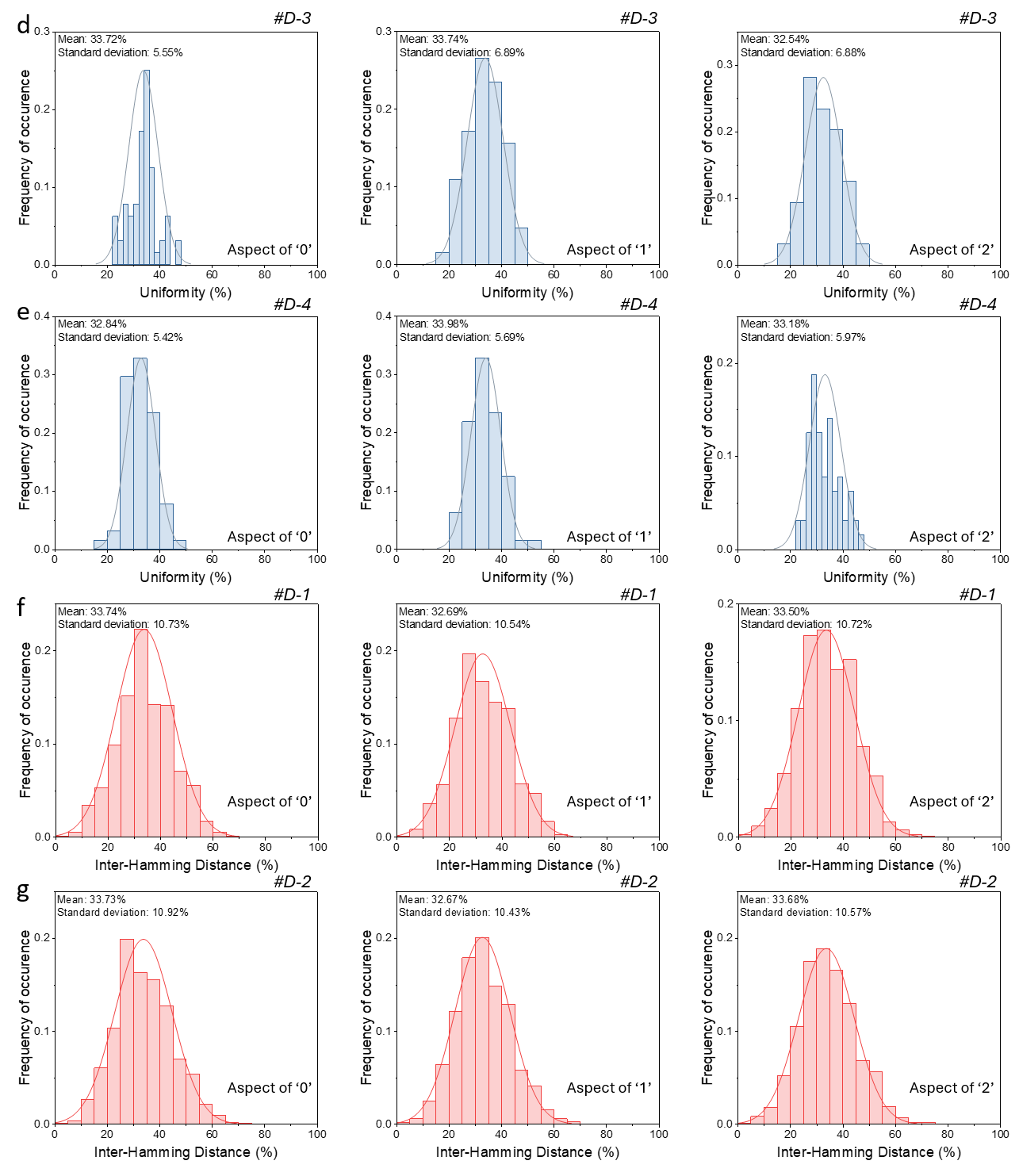


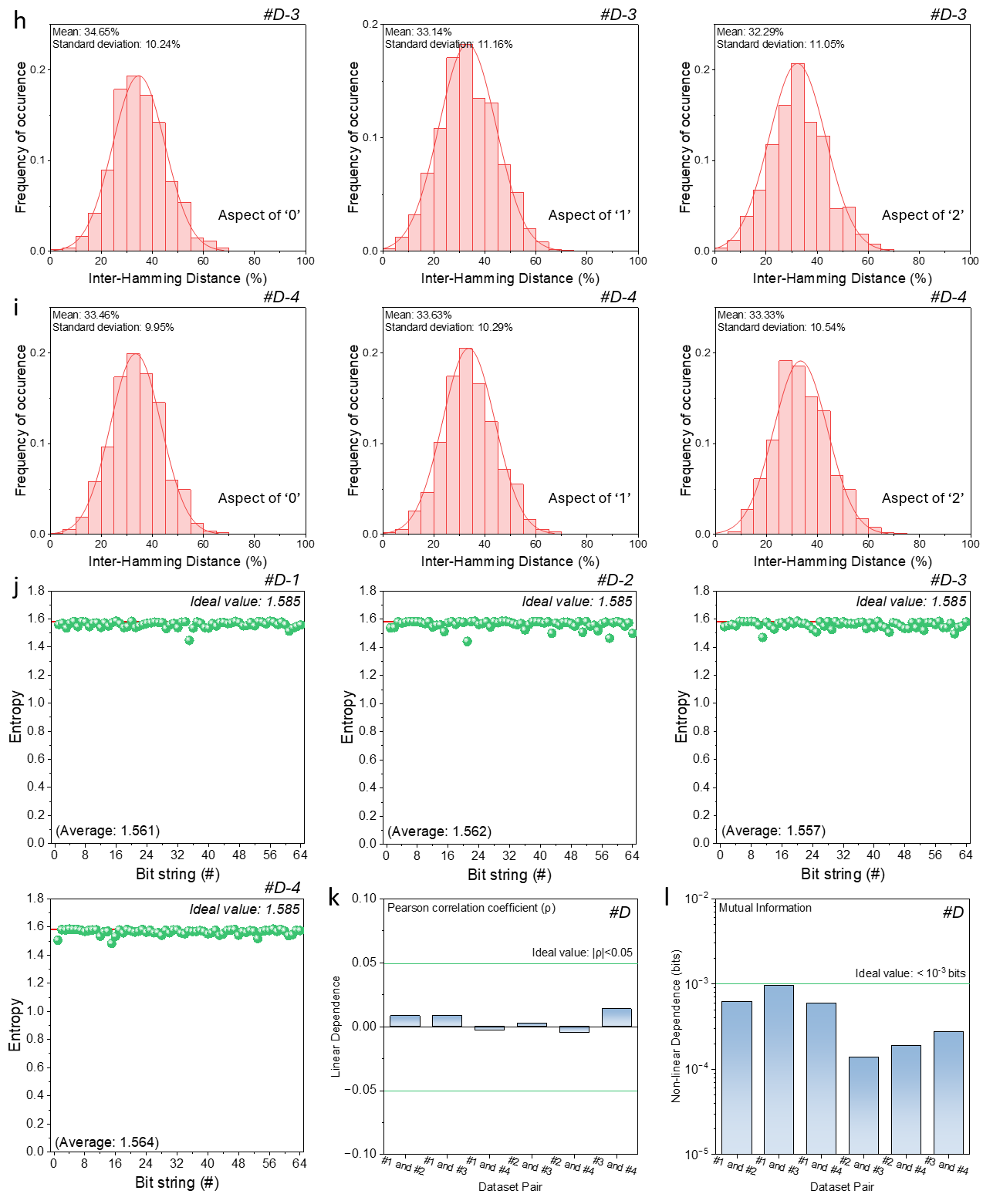


**Figure S28.** Comparison of the bitstreams generated simultaneously from the four samples within group #D. (a) Schematic of the measurement configuration for group #D and the corresponding 8 × 512 mapping. (b–e) Uniformity results for bitstreams D-1 to D-4. (f–i) Inter-HD analysis for bitstreams D-1 to D-4. (j) Entropy values extracted from bitstreams D-1 to D-4. (k) Pearson correlation coefficient and (l) mutual information results assessing the statistical dependence among the four bitstreams in group #D.


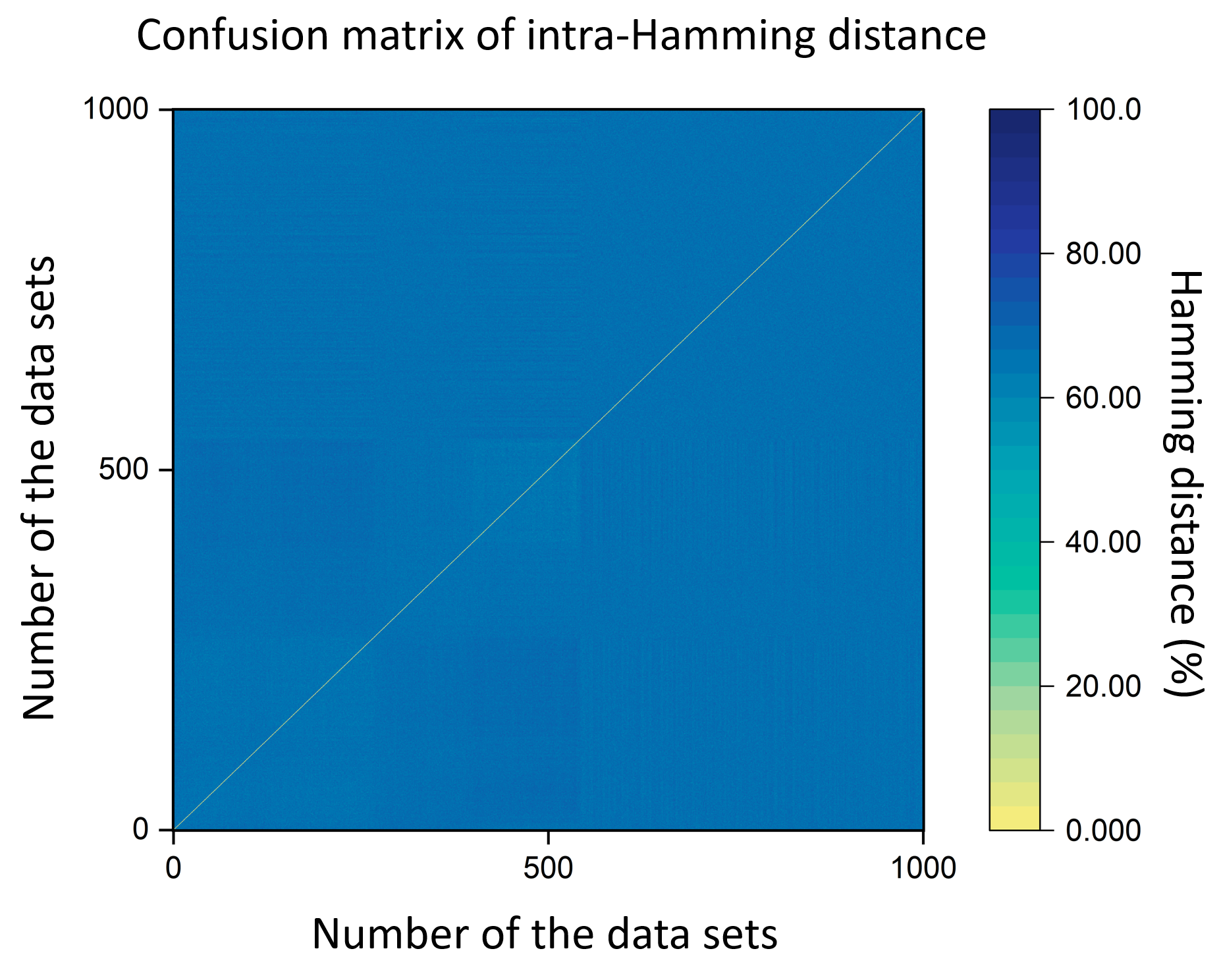


**Figure S29.** Intra-HD evaluation of the PS-TRNG output. Heatmap of pairwise Hamming distances computed across 1,000 ternary maps, each containing 1,024 trits. All off-diagonal comparisons converge to the ideal 66.66% Hamming distance, while only the diagonal entries exhibit zero distance, confirming that every mapping is statistically distinct and independent.


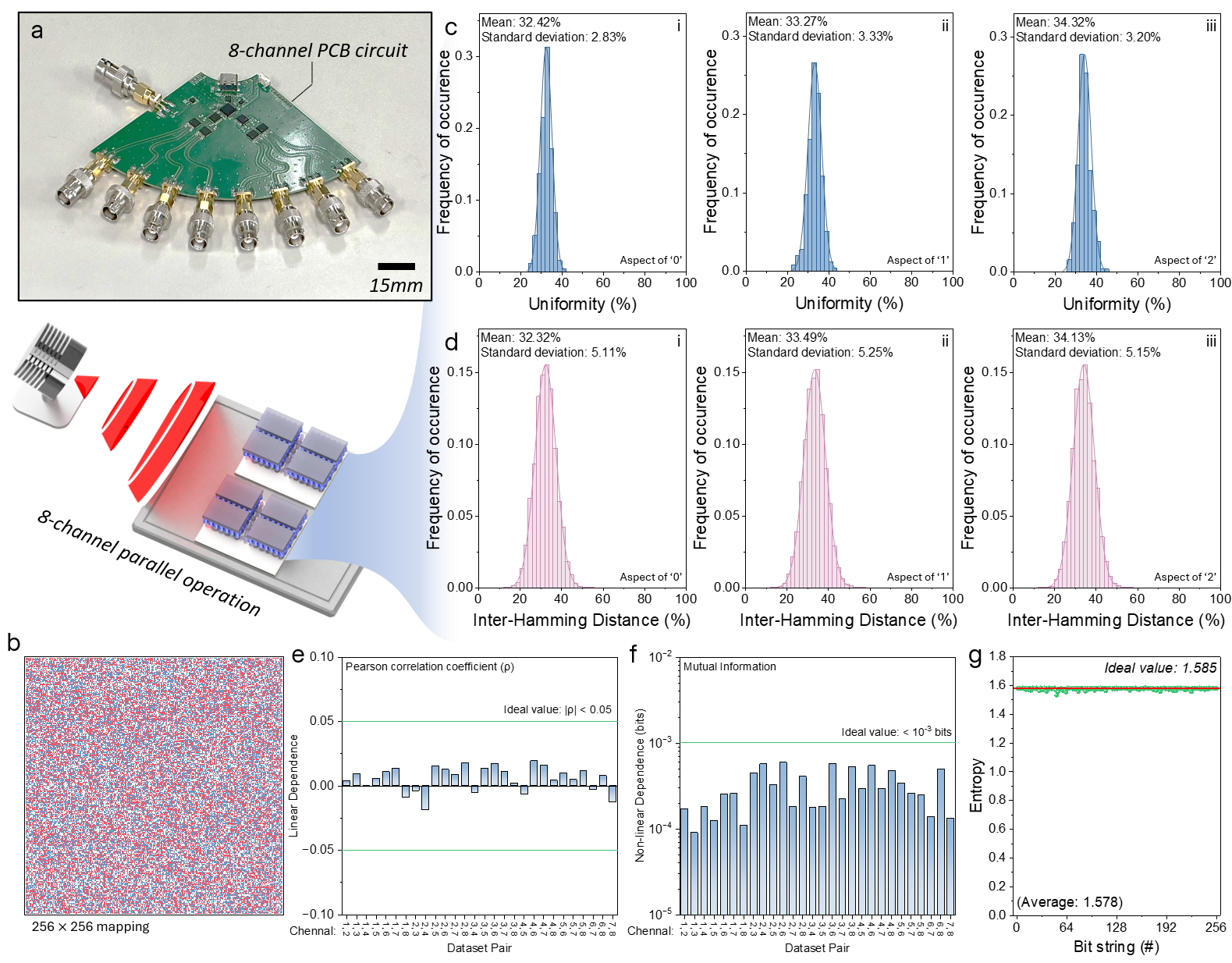


**Figure S30.** Parallel operation of the PS-TRNG using eight samples under identical optical input. (a) Image of the eight-channel measurement circuit and parallel operation setup. (b) Combined 256 × 256 ternary mapping constructed from 8,192 data points per channel. (c) Uniformity distributions for each ternary state: (i) ‘0’, (ii) ‘1’, and (iii) ‘2’. (d) inter-HD distributions evaluated for the three subsets: (i) ‘0’, (ii) ‘1’, and (iii) ‘2’. (e) Pearson correlation coefficient and (f) mutual information analyses for all 28 inter-channel combinations, confirming negligible linear and nonlinear dependencies. (g) Entropy analysis of the generated outputs, showing a value of 1.578 close to the theoretical ideal of 1.585.


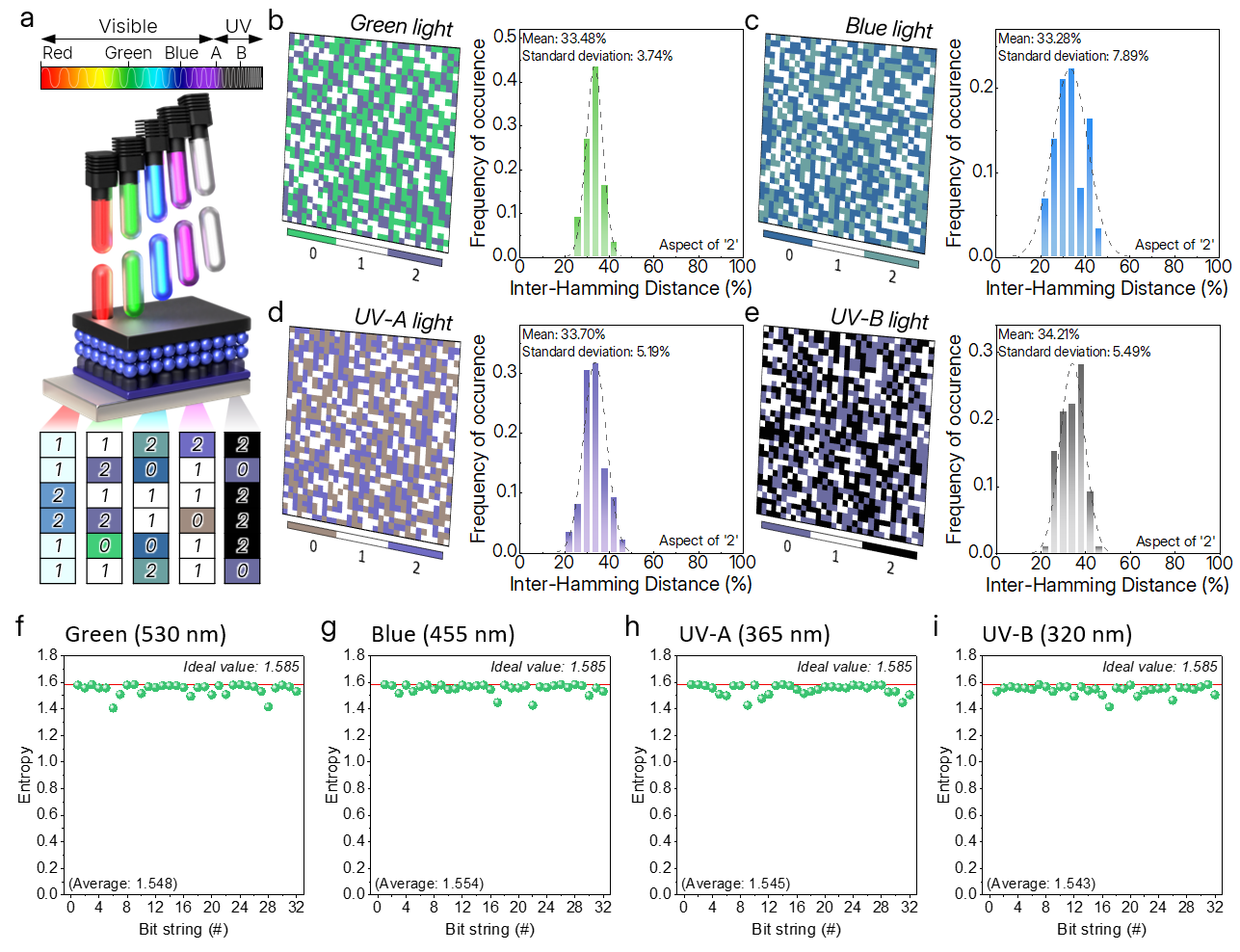


**Figure S31.** Analysis of PS-TRNG performance under varying illumination conditions. (a) Measurement setup illustrating the PS-TRNG device operating under different light conditions. (b–e) Ternary-based security codes and corresponding inter-HD values obtained under green (530 nm), blue (455 nm), UV-A (365 nm), and UV-B (310 nm) illumination, respectively, demonstrating the versatile generation of secure codes across a broad range of wavelengths. (f-i) Entropy analysis of the generated ternary outputs under each illumination condition, confirming wavelength-independent randomness.


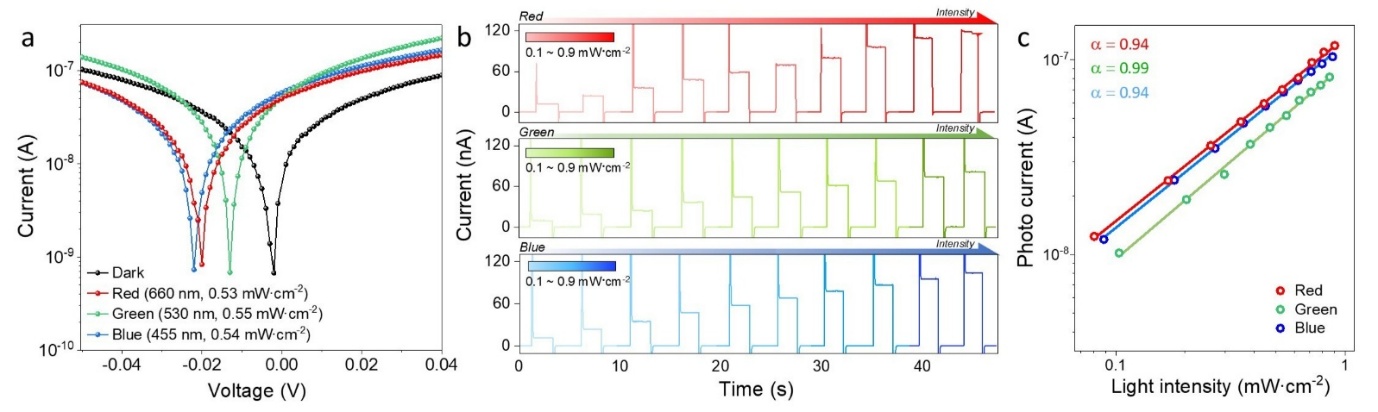


**Figure S32.** Visible-light-induced photoresponses of the PS-TRNG device. (a) Current–voltage characteristics measured under visible light illumination. (b) Photocurrent responses to pulsed red (660 nm), green (530 nm), and blue (455 nm) light sources. (c) Log–log plot of photocurrent versus light intensity, showing power-law exponents (α) of 0.94 (red), 0.99 (green), and 0.94 (blue).


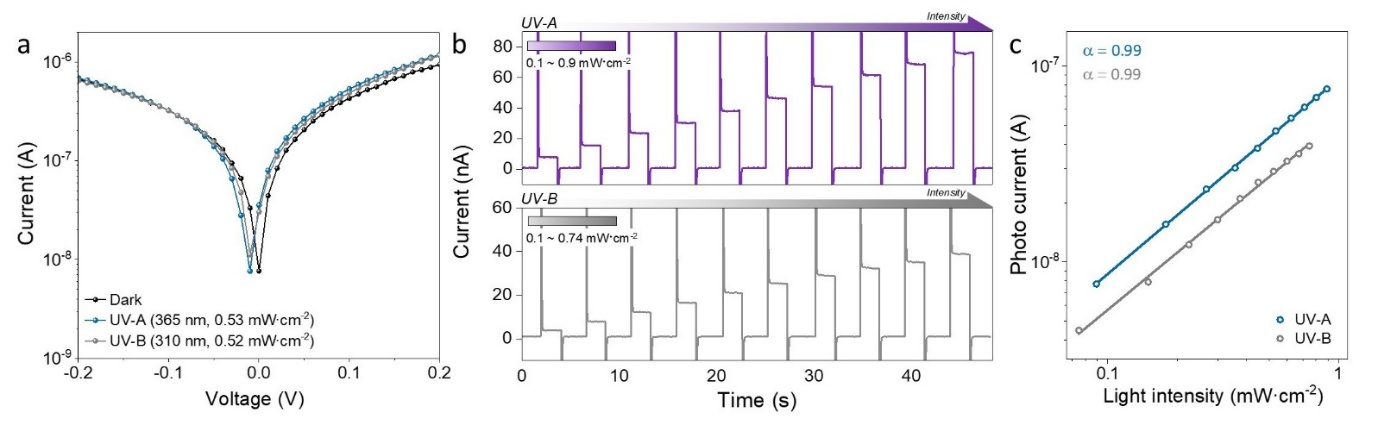


**Figure S33.** Ultraviolet light photoresponses of PS-TRNG. (a) Current–voltage curve and (b) light pulse under UV-A and UV-B light sources of PS-TRNG device. (c) Log–log plot of photocurrent versus light intensity, showing power-law exponents (α) of 0.99 for both UV-A and UV-B.


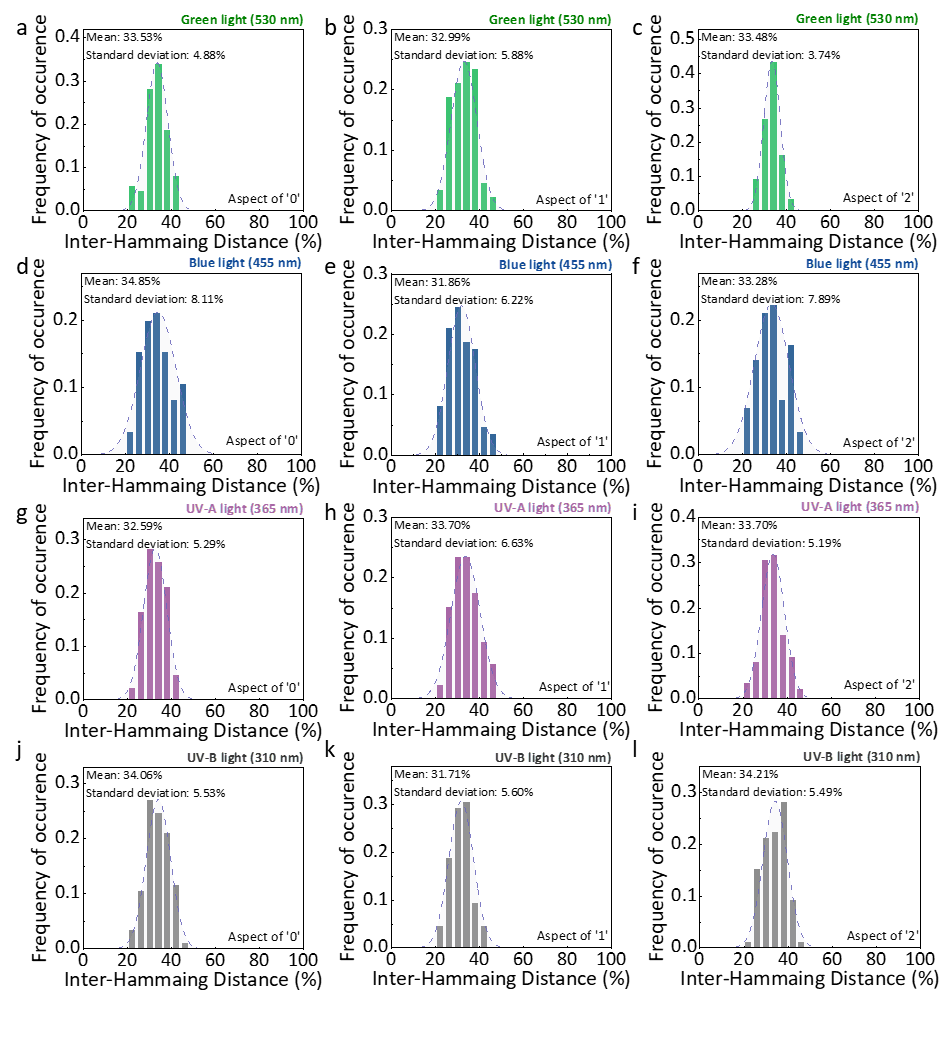


**Figure S34.** Inter-HD of random numbers obtained from various light sources of PS-TRNG. (a–c) Inter-HD of the ‘0’, ‘1’, and ‘2’ trits under green light. (d–f) Inter-HD of the ‘0’, ‘1’, and ‘2’ trits under blue light. (g–i) Inter-HD of the ‘0’, ‘1’, and ‘2’ trits under UV-A light. (j–l) Inter-HD of the ‘0’, ‘1’, and ‘2’ trits under UV-B light.


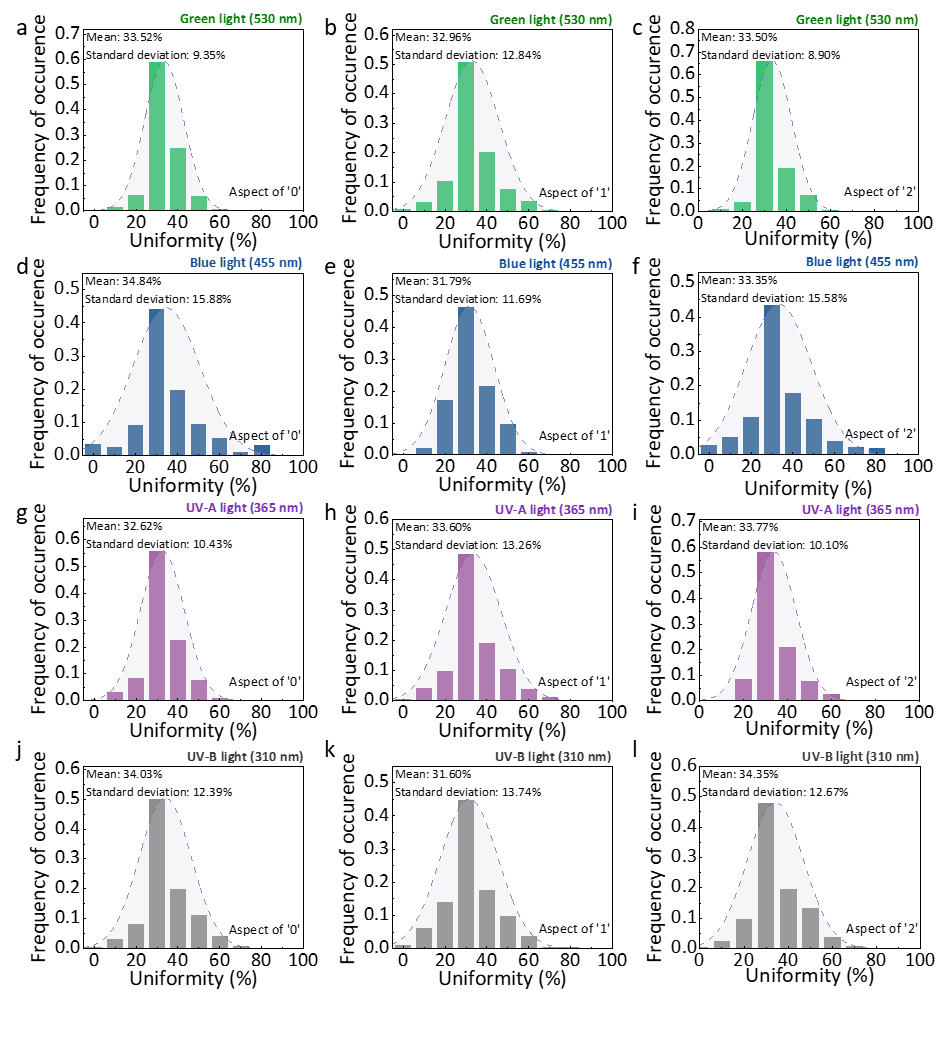


**Figure S35.** Uniformity of random numbers obtained from various light sources of PS-TRNG. (a–c) Uniformity of the ‘0’, ‘1’, and ‘2’ trits under green light. (d–f) Uniformity of the ‘0’, ‘1’, and ‘2’ trits under blue light. (g–i) Uniformity of the ‘0’, ‘1’, and ‘2’ trits under UV-A light. (j–l) Uniformity of the ‘0’, ‘1’, and ‘2’ trits under UV-B light.


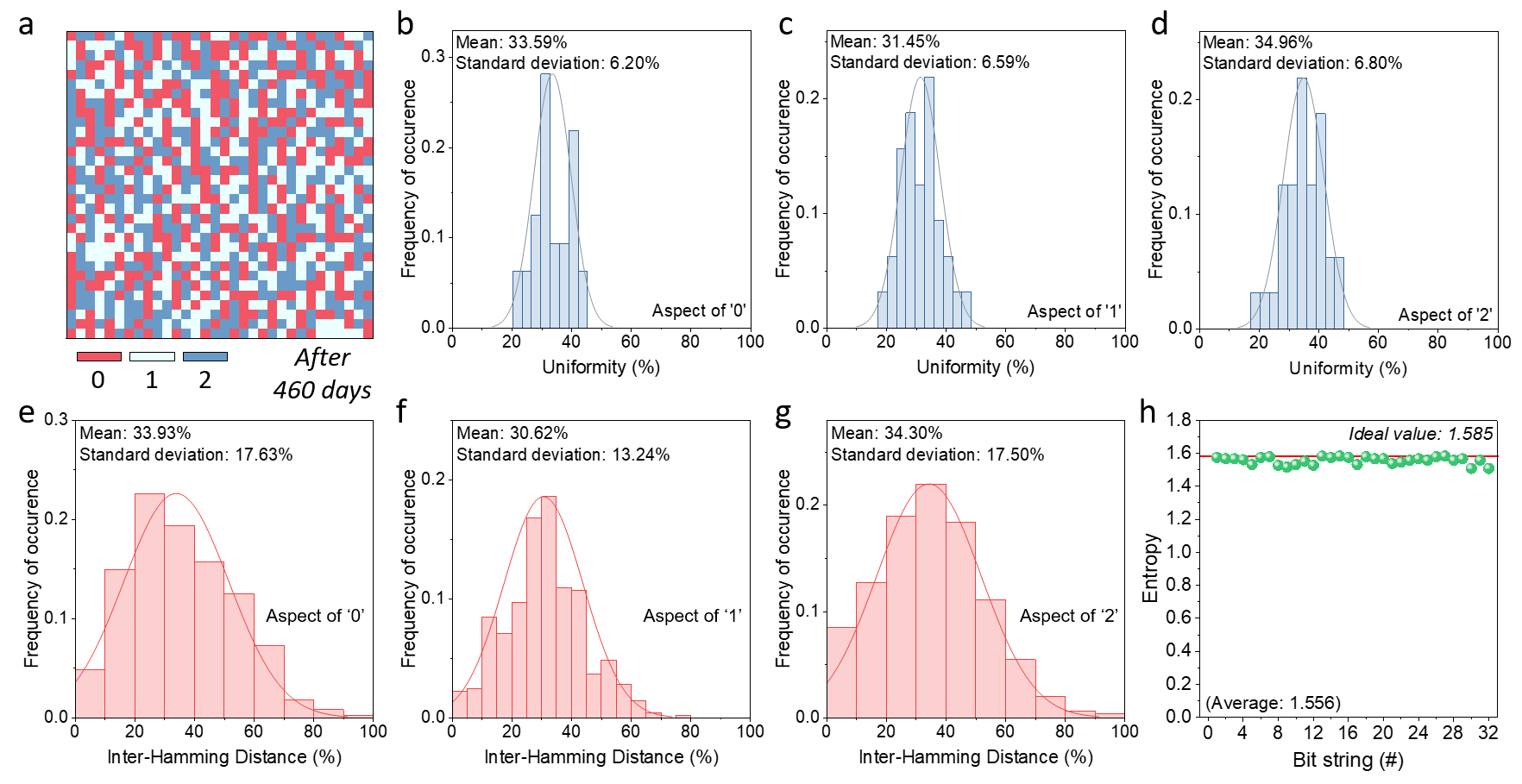


**Figure S36.** Long-term stability and randomness analysis of the PS-TRNG measured after 460 days. (a) Trit map obtained from the PS-TRNG after 460 days. (b–d) Uniformity analysis of the ‘0’, ‘1’, and ‘2’ trits, respectively. (e–g) Inter-HD analysis for the ‘0’, ‘1’, and ‘2’ trits. (h) Entropy analysis results obtained from the 460-day long-term stability test.


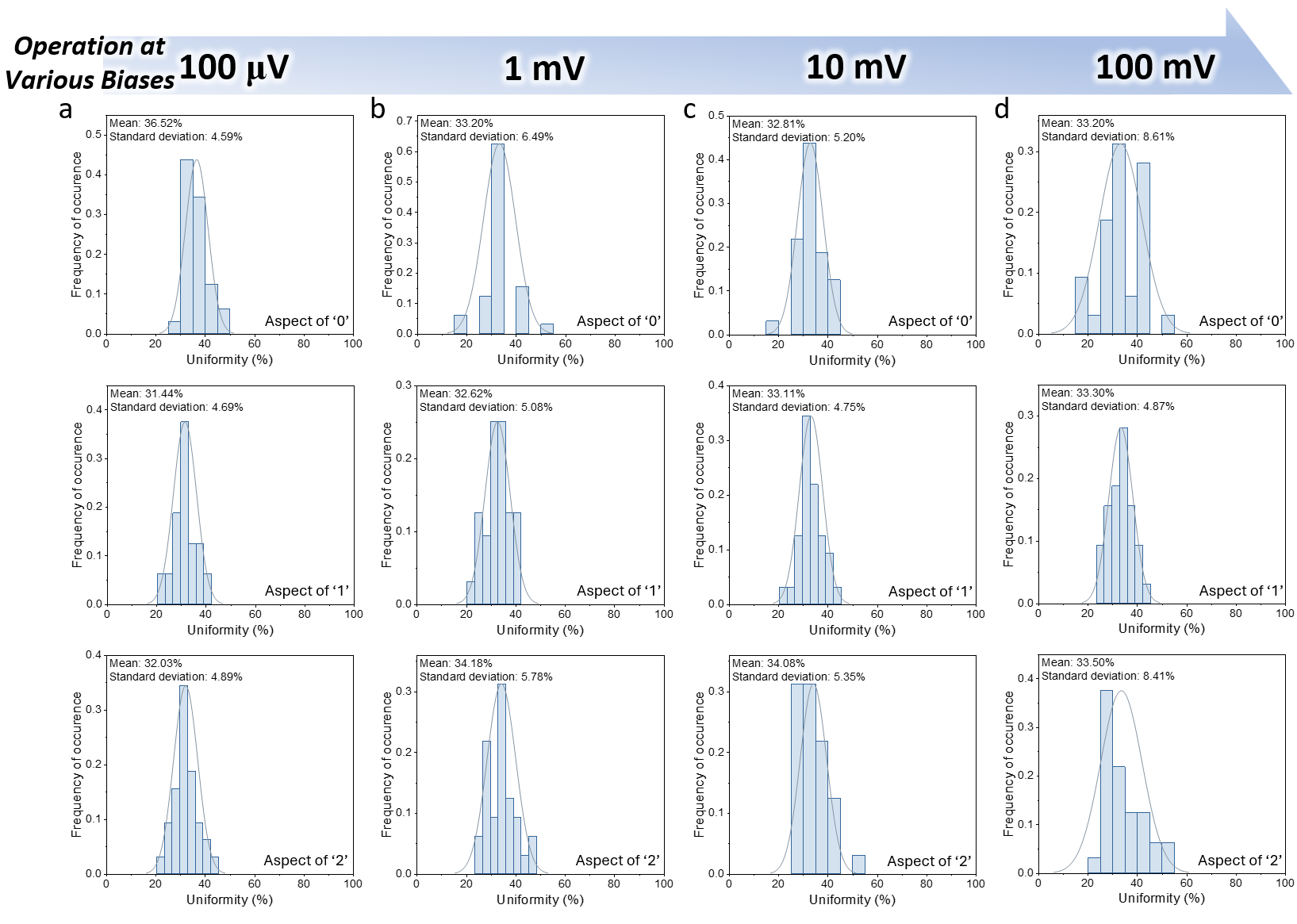


**Figure S37.** Uniformity analysis of the PS-TRNG under various bias operation modes. (a) 100 μV, (b) 1 mV, (c) 10 mV, and (d) 100 mV, showing that the ternary output maintains a balanced distribution among the three logic states (‘0’, ‘1’, and ‘2’) across all bias levels.


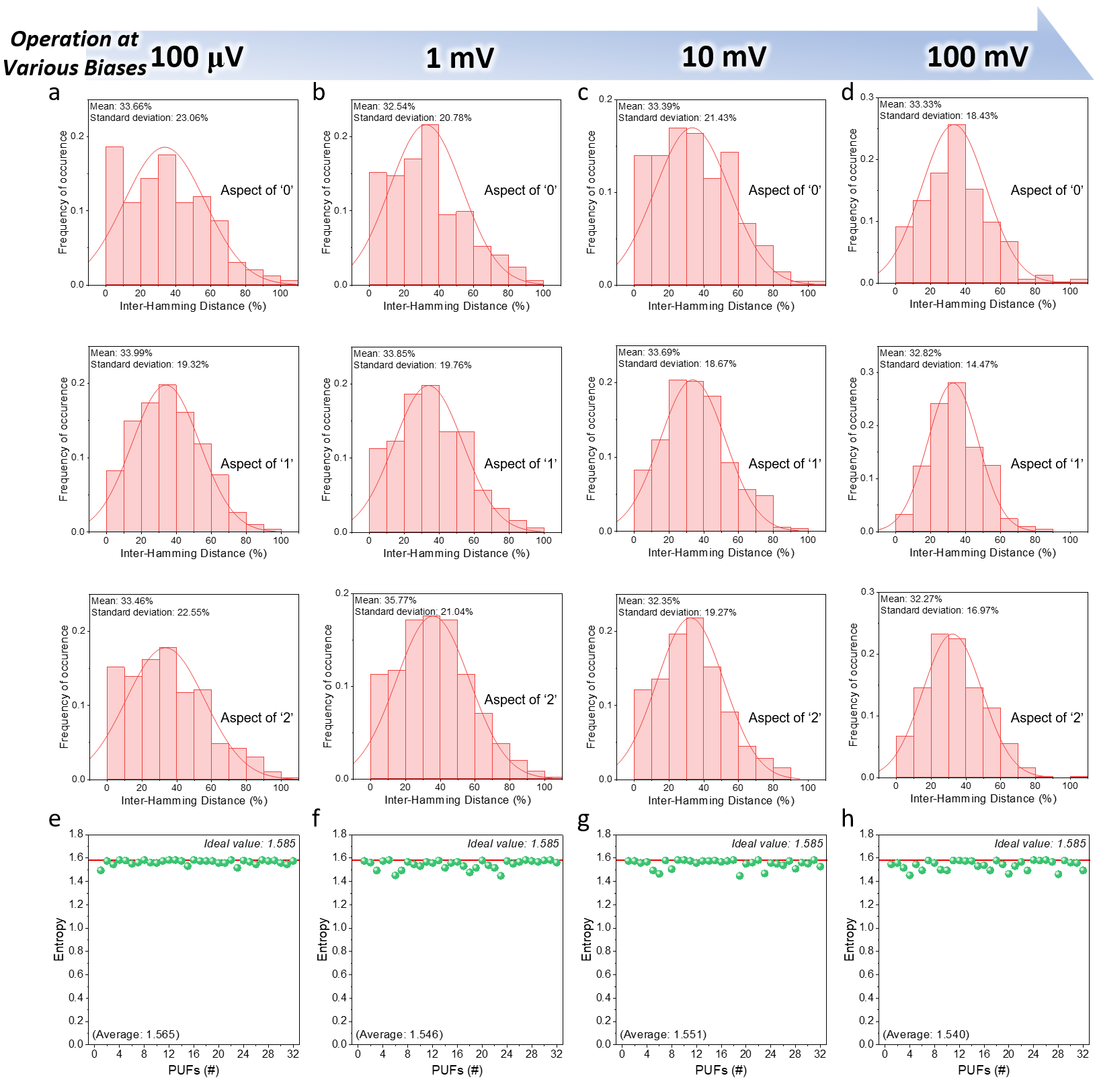


**Figure S38.** Inter-HD and entropy characteristics of the PS-TRNG under different bias operation modes. (a–d) Inter-HD results and (e–h) corresponding entropy analysis at 100 μV, 1 mV, 10 mV, and 100 mV, respectively.


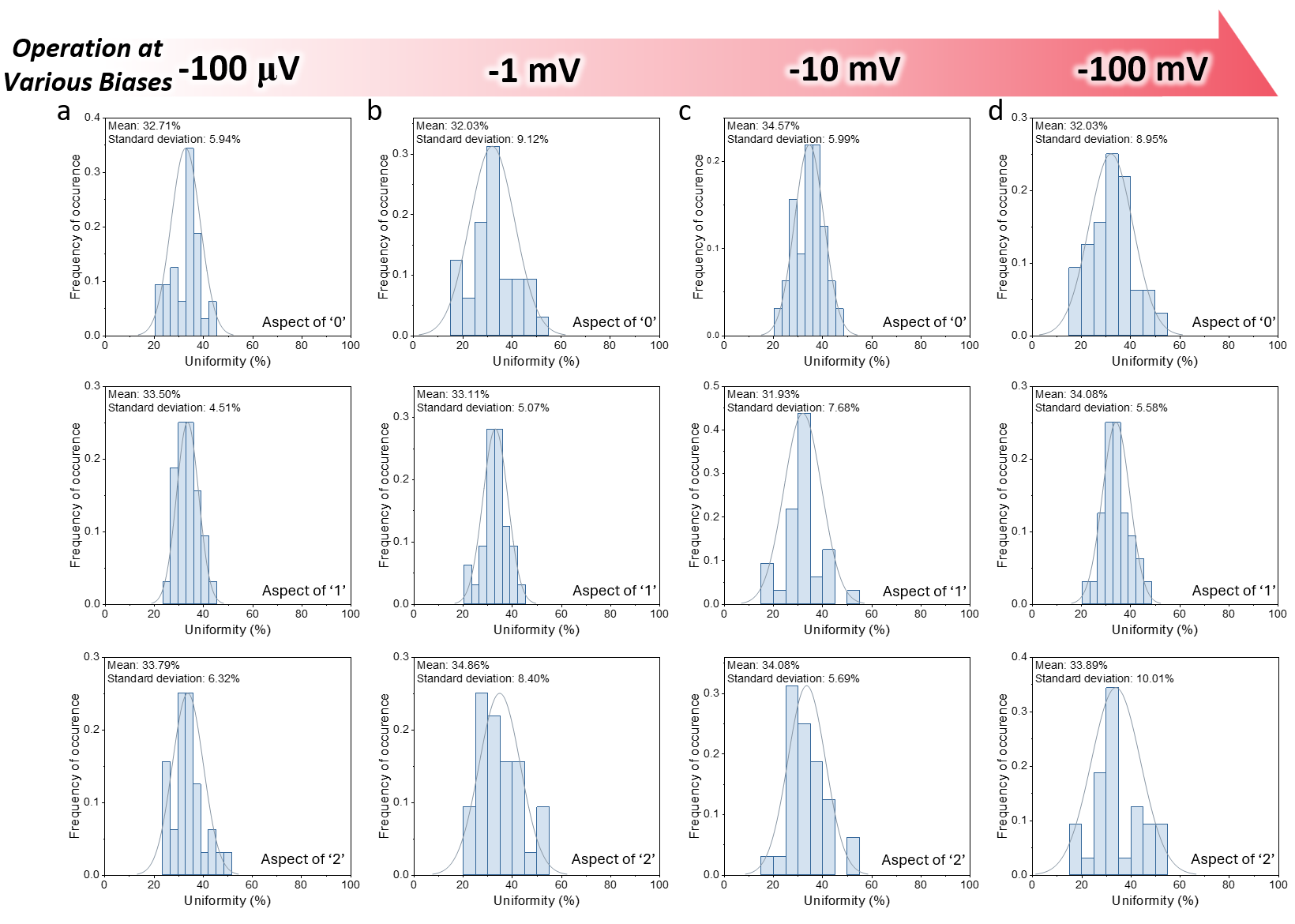


**Figure S39.** Statistical uniformity of the PS-TRNG under negative bias modes. (a) −100 μV, (b) −1 mV, (c) −10 mV, and (d) −100 mV. The ternary output retained a well-balanced distribution among the three logic states (‘0’, ‘1’, and ‘2’) across all negative bias levels.


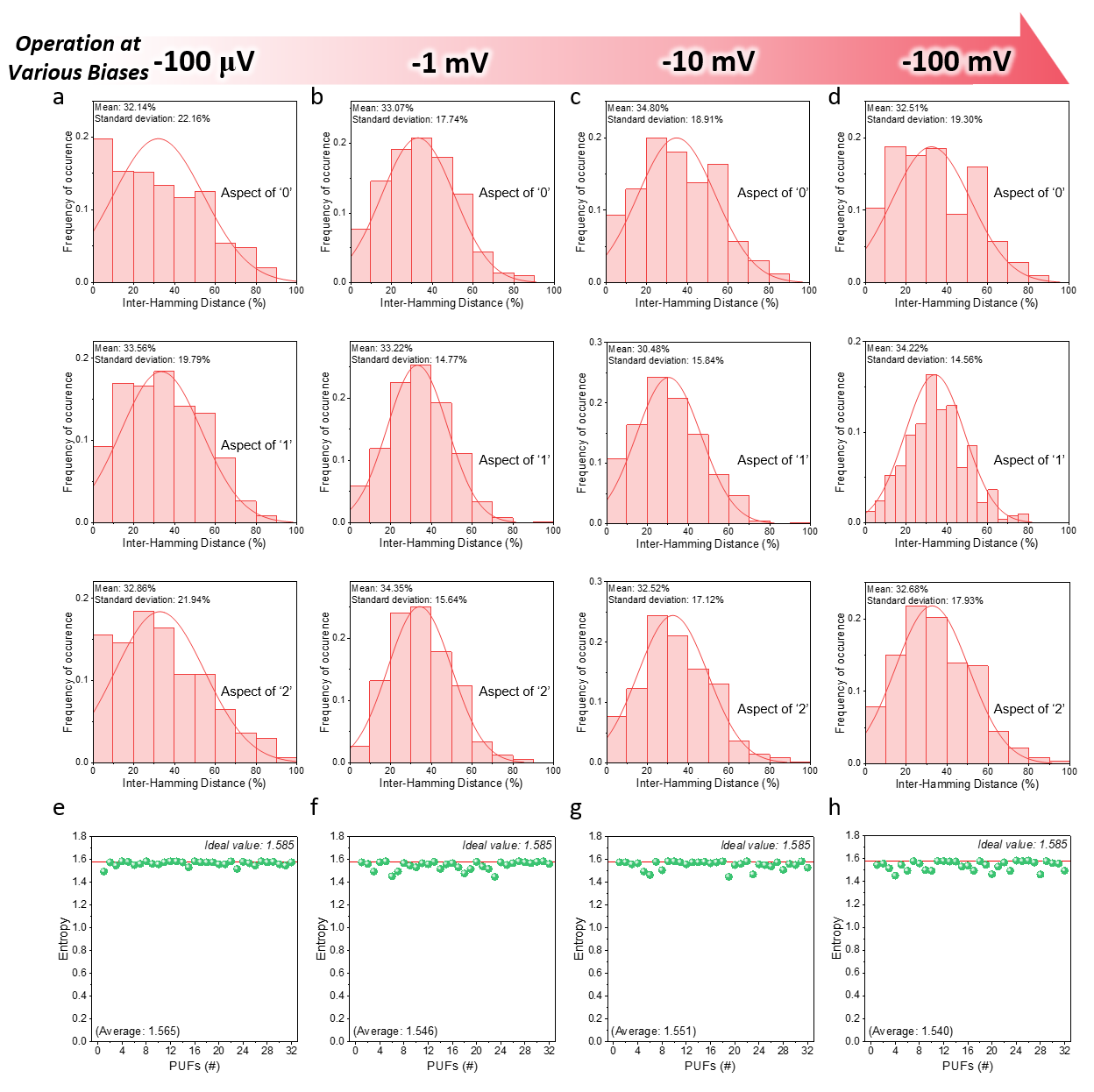


**Figure S40.** Inter-HD entropy evaluation of the PS-TRNG under negative bias operation. (a–d) Inter-HD analysis and (e–h) entropy at −100 μV, −1 mV, −10 mV, and −100 mV, respectively, demonstrating sustained randomness and consistent entropy behavior under negative bias conditions.


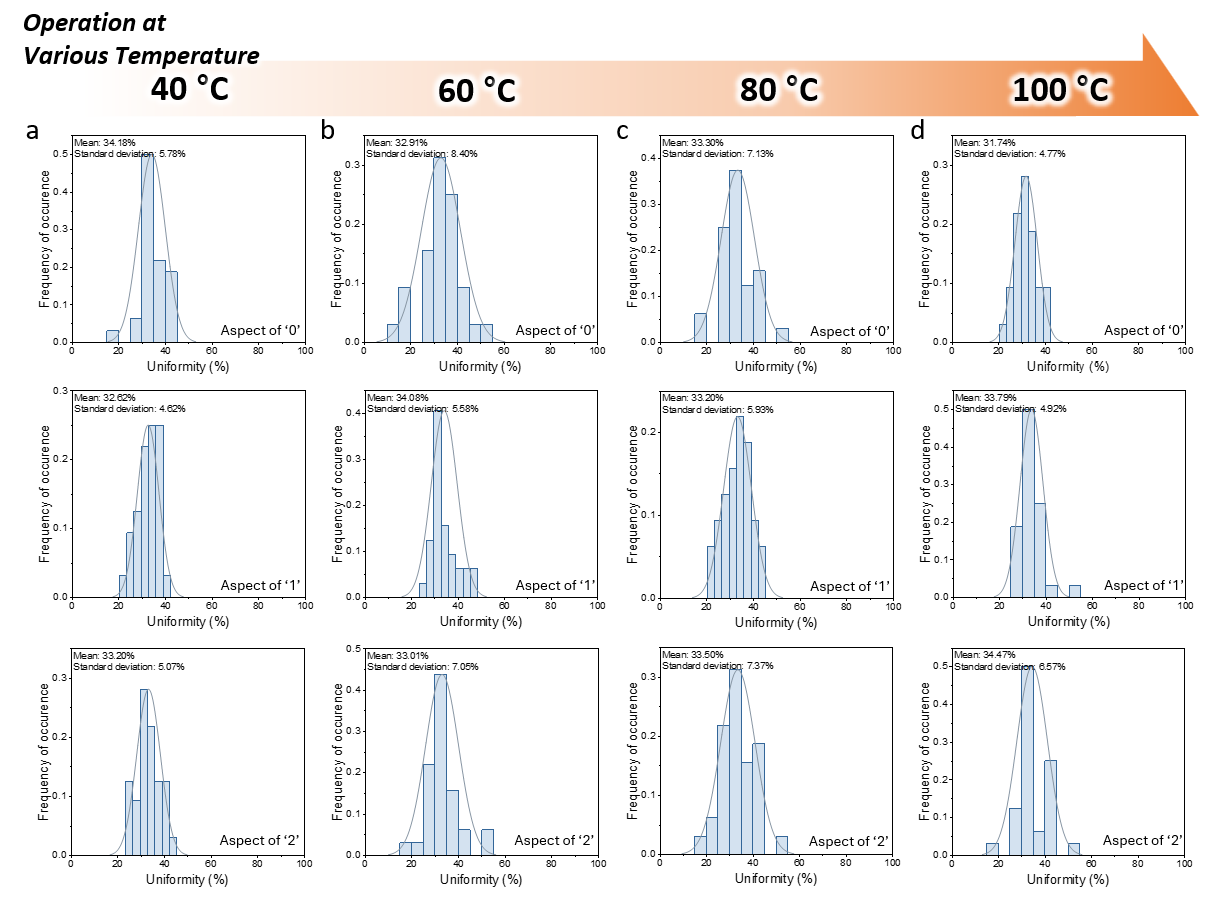


**Figure S41.** Plots of uniformity of the generated ternary random numbers measured under different temperature conditions, showing stable statistical behavior at (a) 40 ℃, (b) 60 ℃, (c) 80 ℃, and (d) 100 ℃.


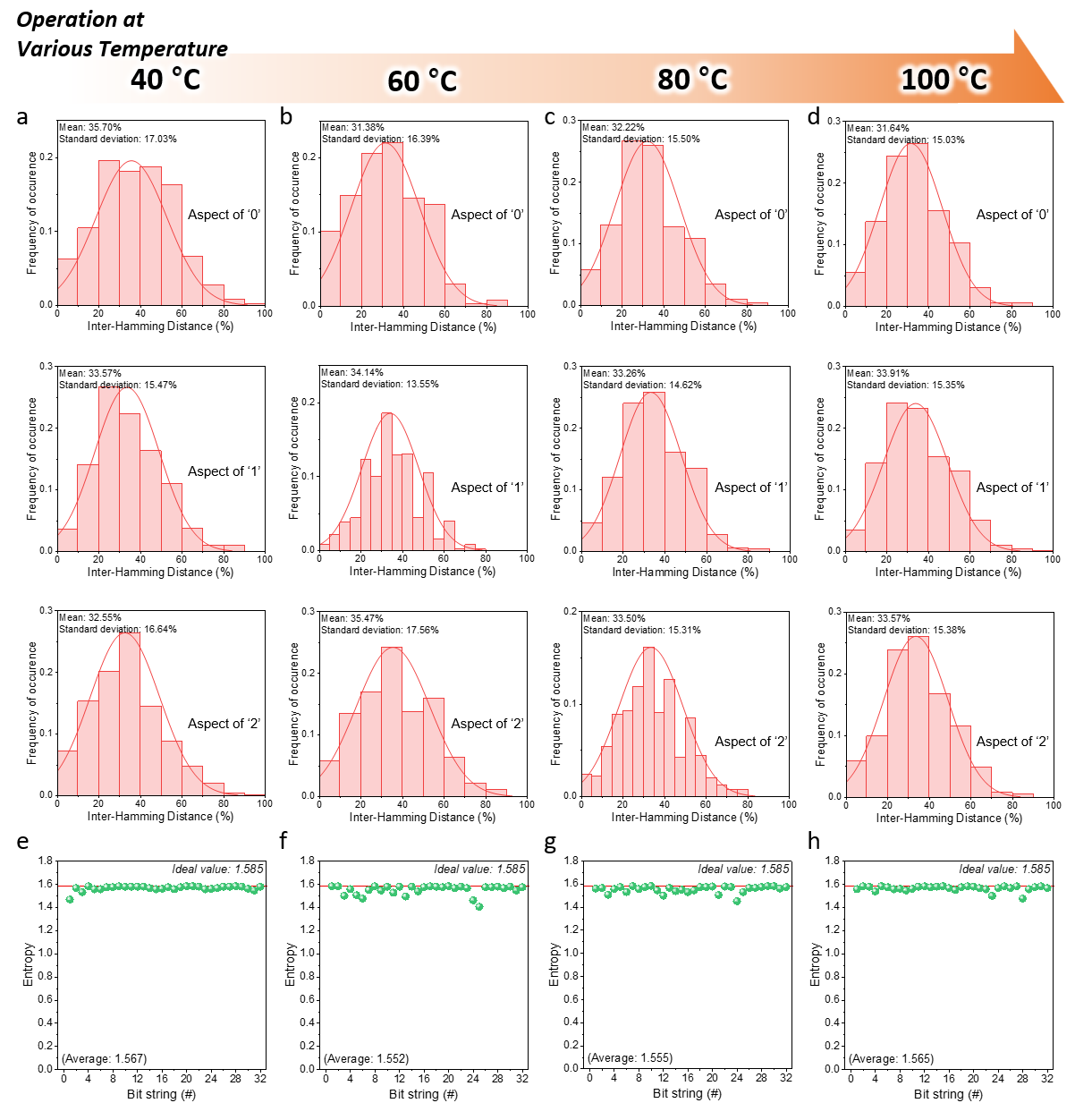


**Figure S42.** Plots of inter-HD and entropy of the generated ternary random numbers measured under different temperature conditions, confirming stable randomness characteristics at (a) 40 ℃, (b) 60 ℃, (c) 80 ℃, and (d) 100 ℃.


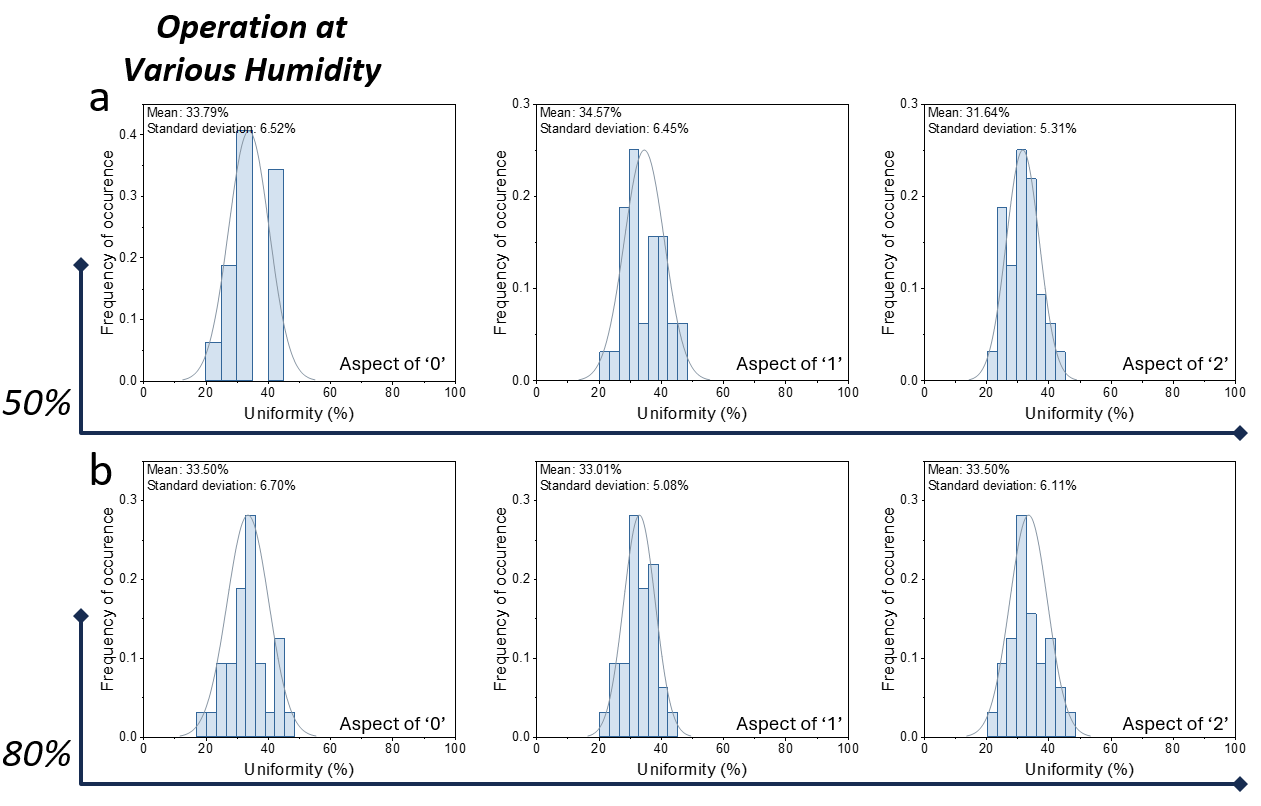


**Figure S43.** Plots of uniformity of the generated ternary random numbers measured under different humidity conditions, showing stable statistical behavior at (a) 50 % and (b) 80 % relative humidity.


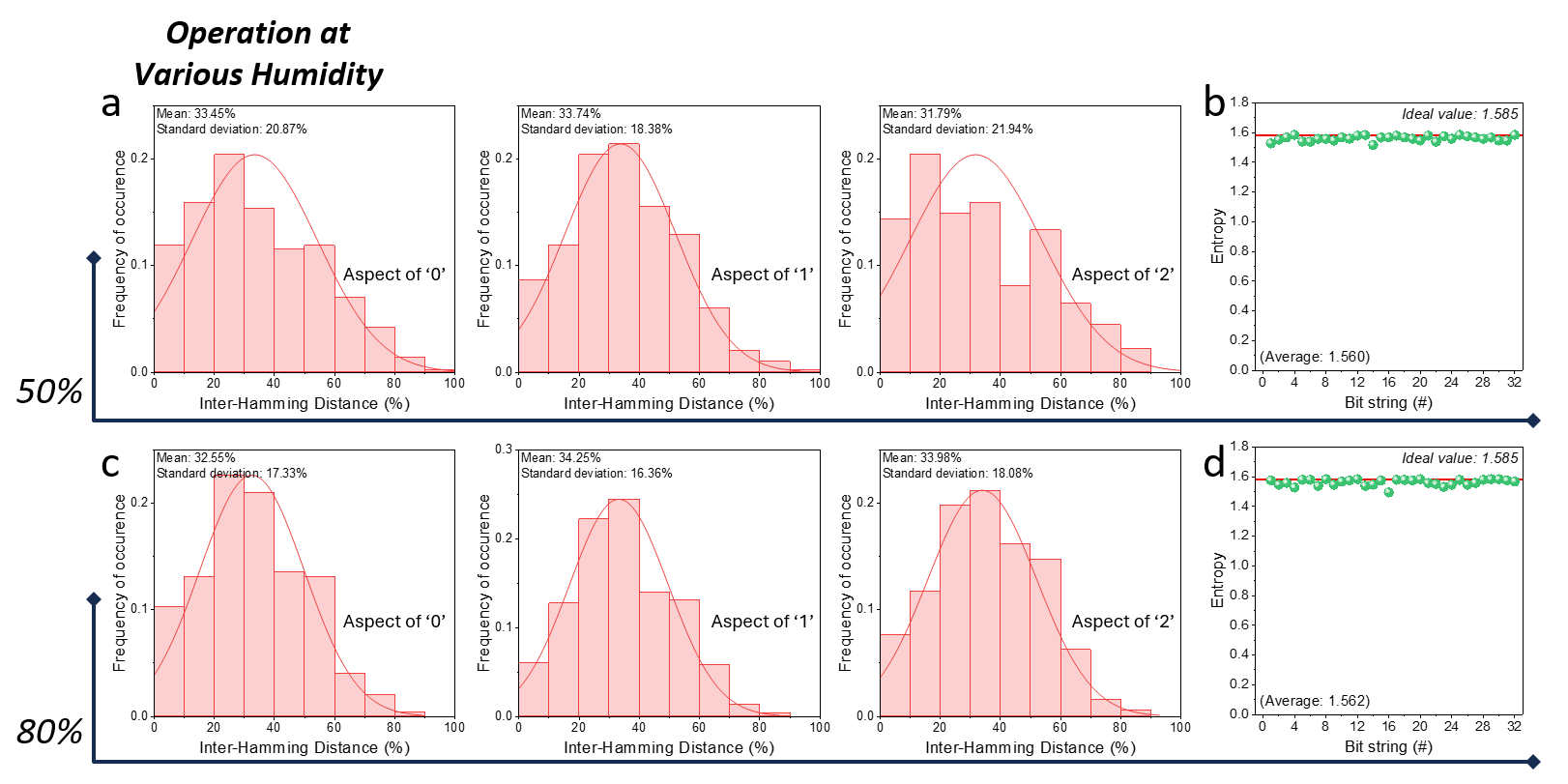


**Figure S44.** Plots of inter-HD and entropy of the generated ternary random numbers measured under different humidity conditions, confirming stable randomness at (a) 50 % and (b) 80 % relative humidity.


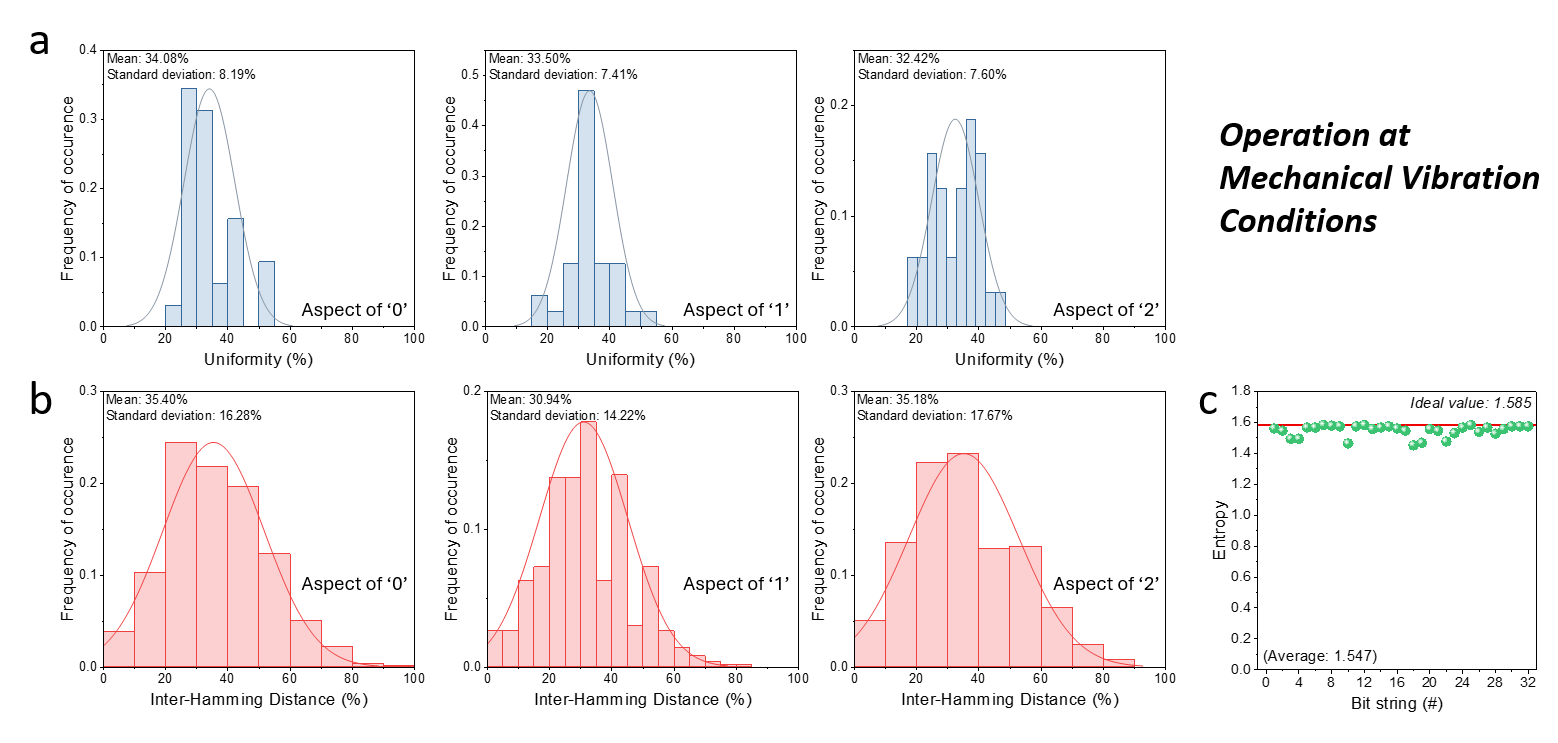


**Figure S45.** Plots of (a) uniformity, (b) inter-HD, and (c) entropy of the generated ternary random numbers measured under mechanical vibration, confirming stable and unbiased randomness when operated in front of a 132 mW vibration module.


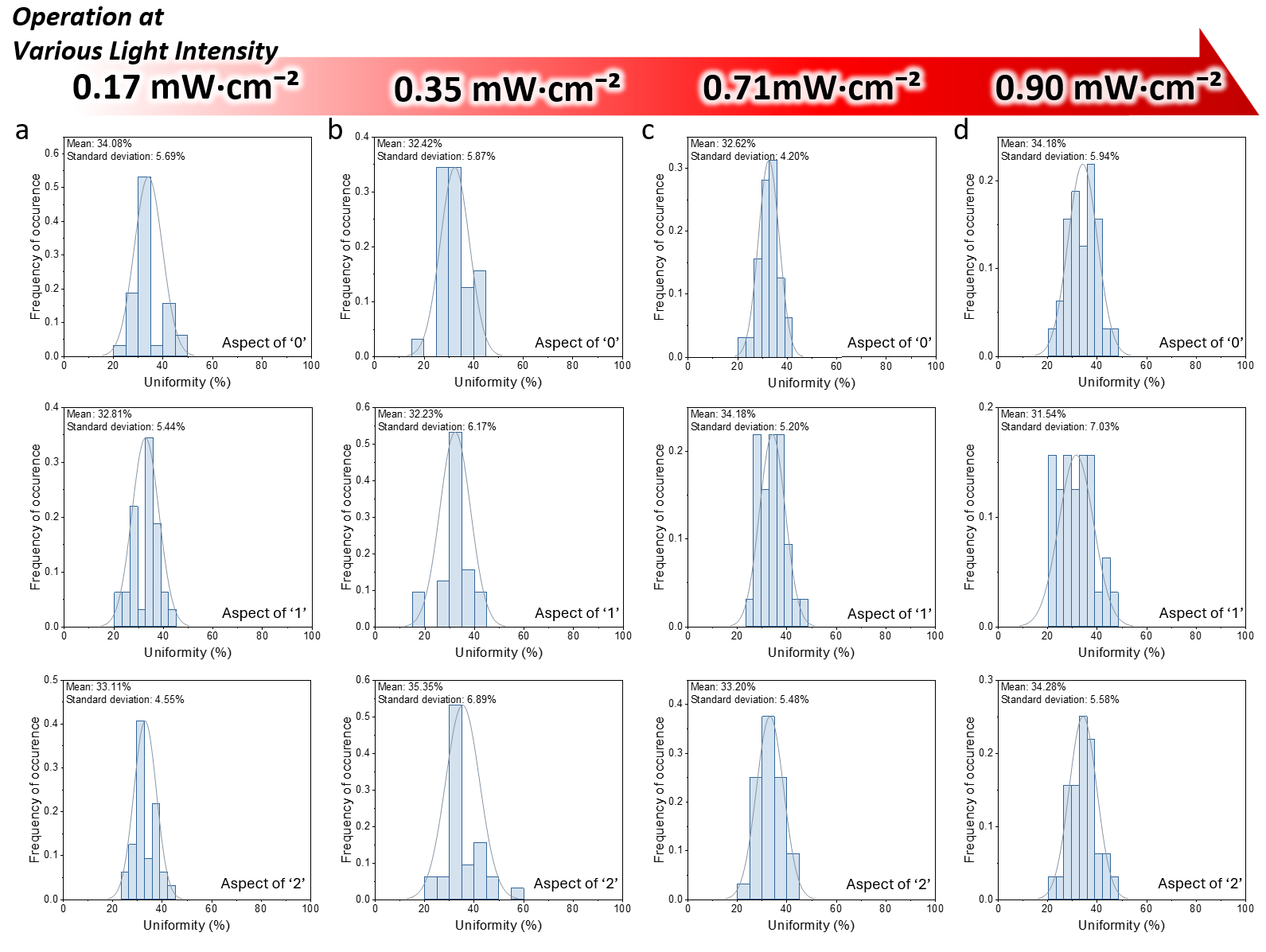


**Figure S46.** Uniformity analysis of the generated ternary random numbers under varying optical power densities: (a) 0.17, (b) 0.35, (c) 0.71, and (d) 0.90 mW·cm^-2^, showing the statistical distribution of occurrence ratios for trits ‘0’, ‘1’, and ‘2’.


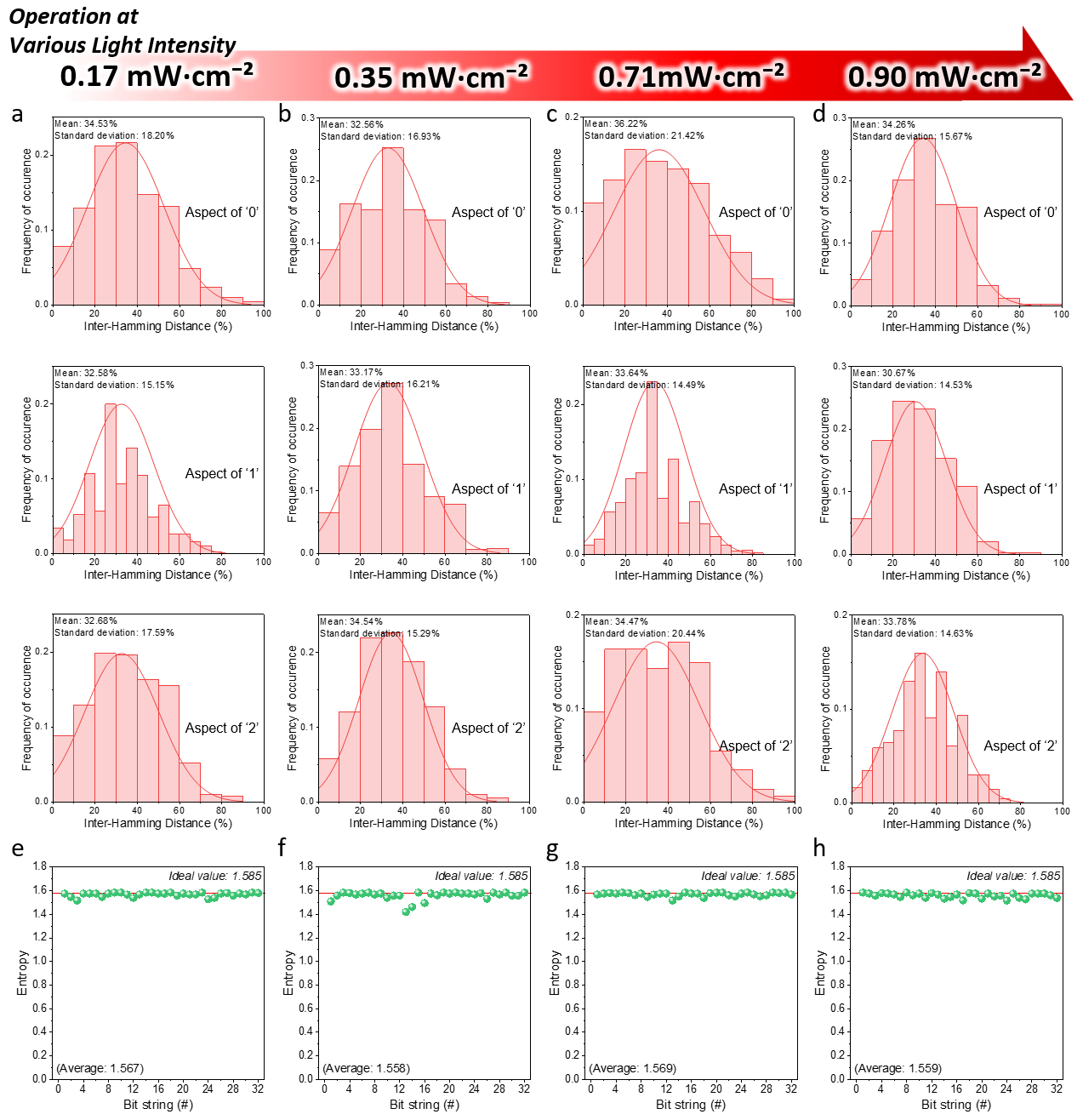


**Figure S47.** Inter-HD and entropy analysis of the generated ternary random numbers under varying optical power densities. (a–d) Inter-HD distributions obtained at optical power densities of 0.17, 0.35, 0.71, and 0.90 mW·cm^-2^. (e–h) Corresponding entropy values extracted under each illumination condition.


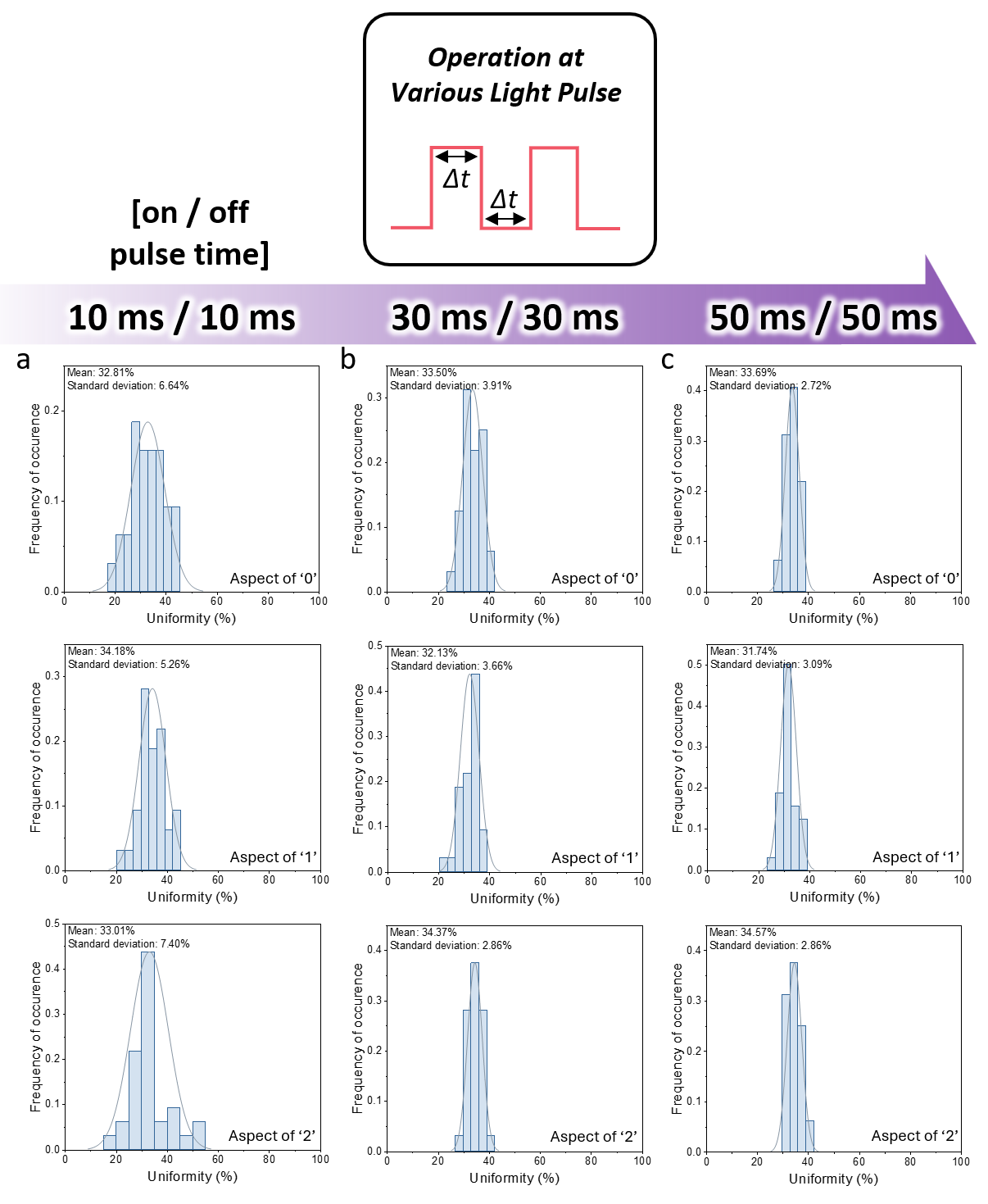


**Figure S48.** Uniformity analysis of the generated ternary random numbers measured under different optical pulse conditions. Panels (a) 10 ms/10 ms, (b) 30 ms/30 ms, and (c) 50 ms/50 ms present the uniformity histograms for trits ‘0’, ‘1’, and ‘2’.


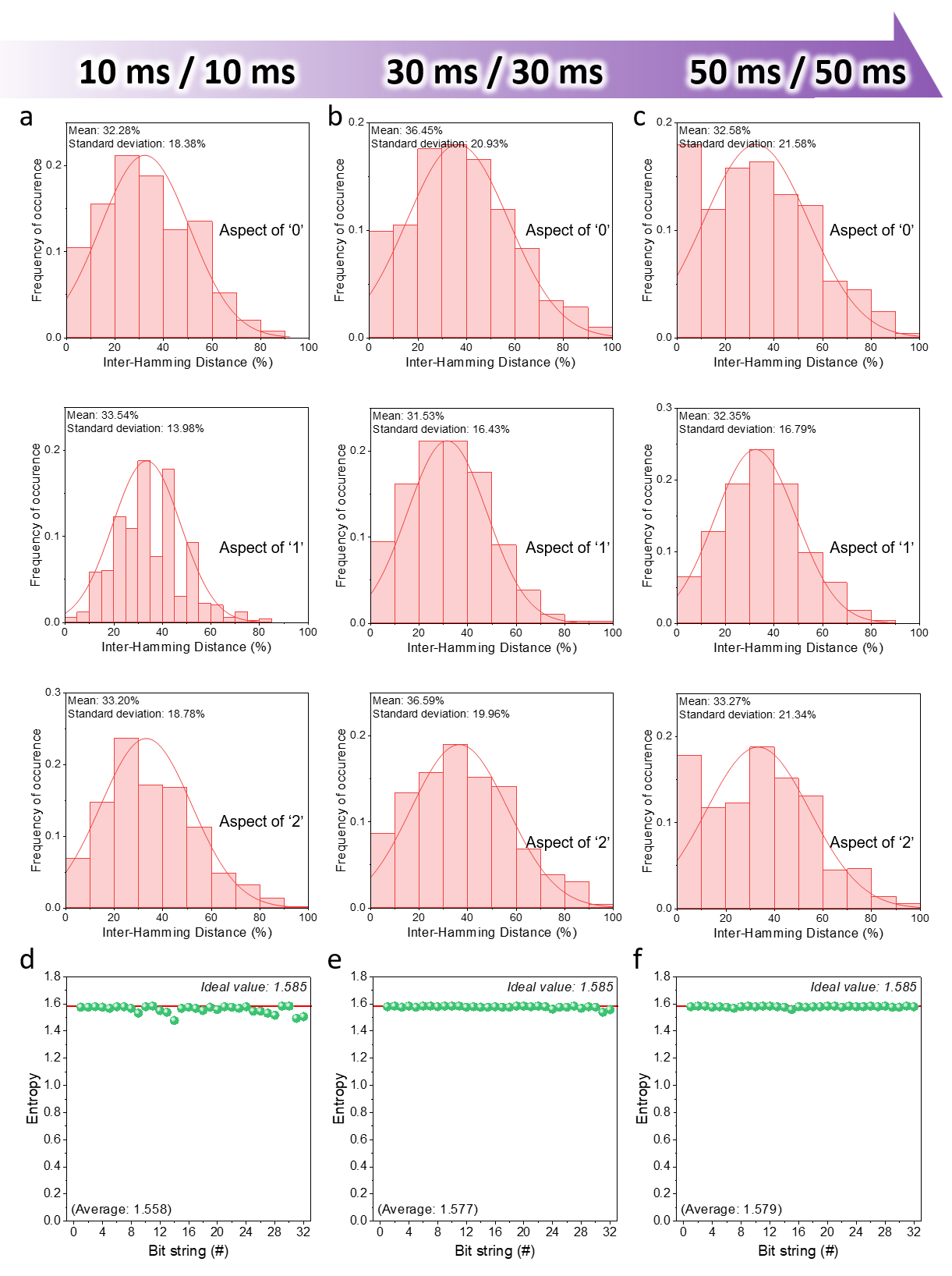


**Figure S49.** Inter-HD and entropy analysis of the generated ternary random numbers under varying optical pulse conditions. (a–c) Inter-HD distributions obtained at optical pulse conditions of 10 ms/10 ms, 30 ms/30 ms, and 50 ms/50 ms. (e–h) Corresponding entropy values extracted under each illumination condition.


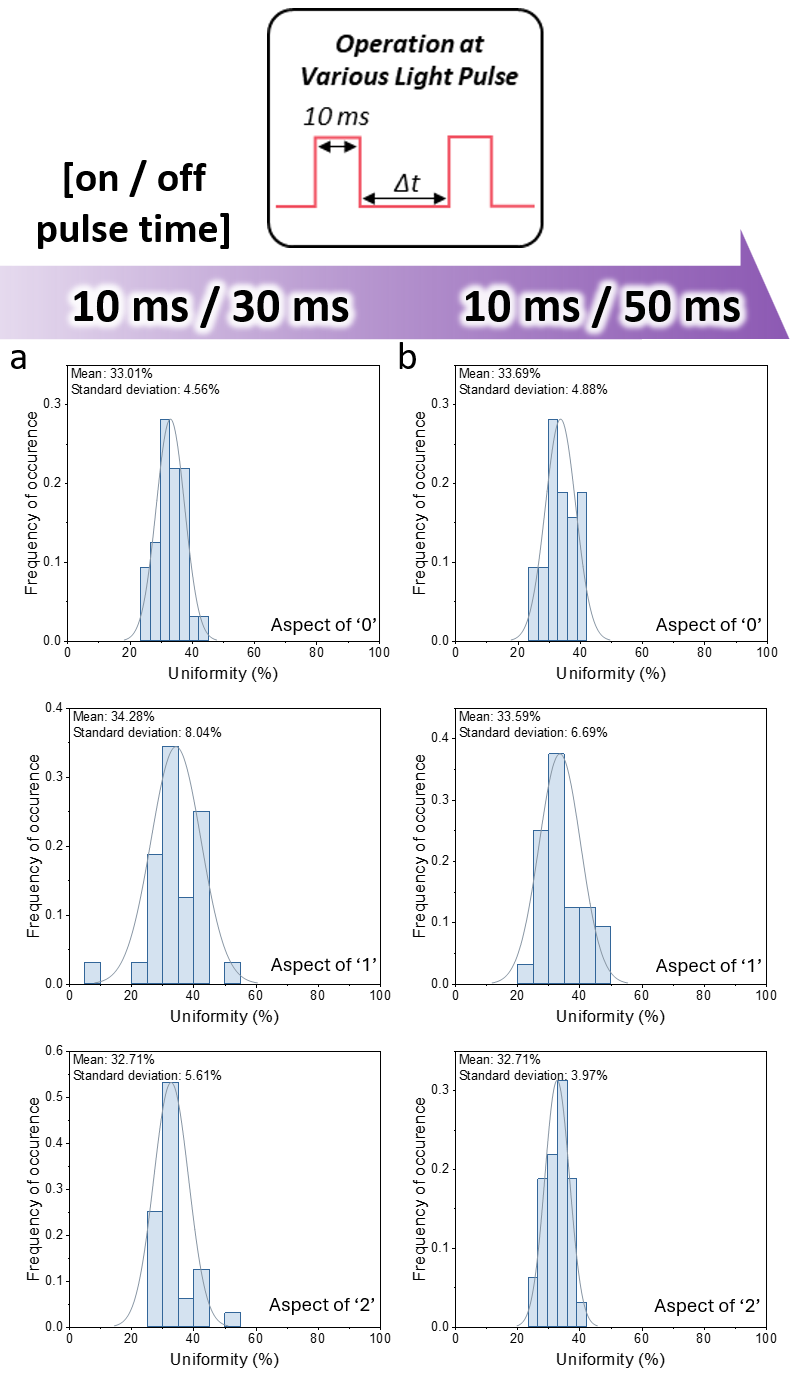


**Figure S50.** Uniformity analysis of the generated ternary random numbers measured under different optical pulse conditions. Panels (a) 10 ms/30 ms, and (b) 10 ms/50 ms present the uniformity histograms for trits ‘0’, ‘1’, and ‘2’.


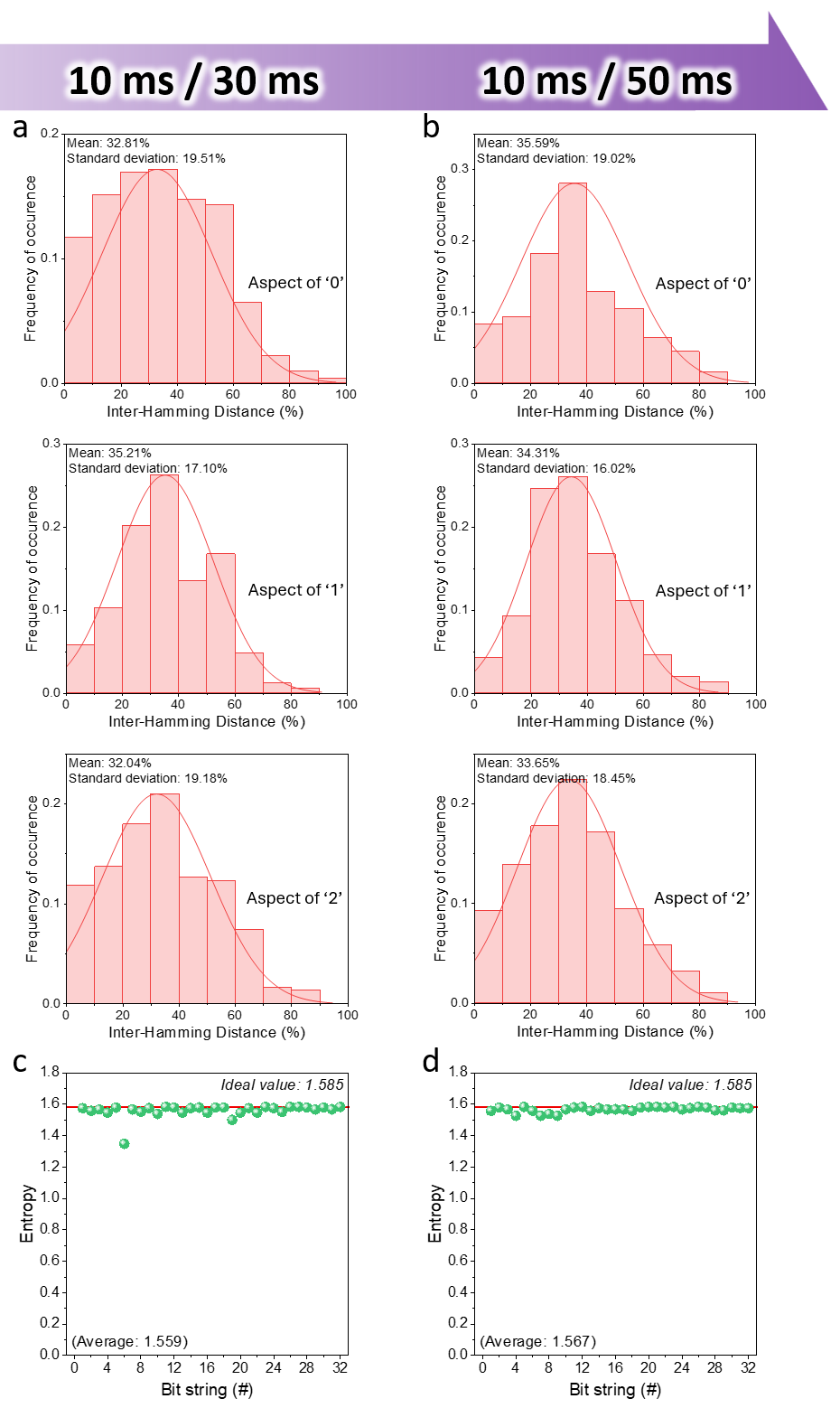


**Figure S51.** Inter-HD and entropy analysis of the generated ternary random numbers under varying optical pulse conditions. Inter-HD distributions obtained at optical pulse conditions of (a) 10 ms/30 ms, and (b) 10 ms/50 ms. (c-d) Corresponding entropy values extracted under each illumination condition.


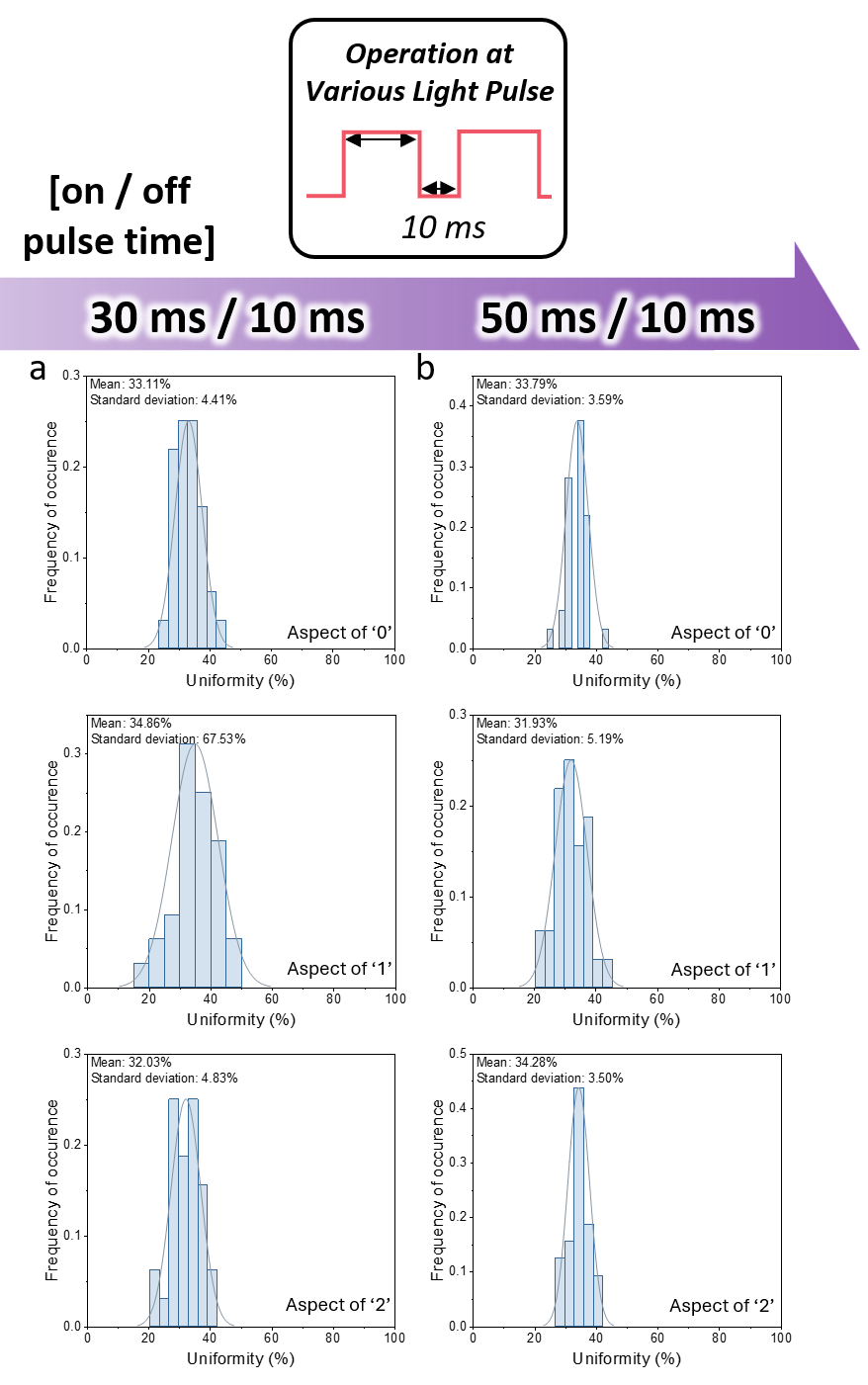


**Figure S52.** Uniformity analysis of the generated ternary random numbers measured under different optical pulse conditions. Panels (a) 30 ms/10 ms, and (b) 50 ms/10 ms present the uniformity histograms for trits ‘0’, ‘1’, and ‘2’.


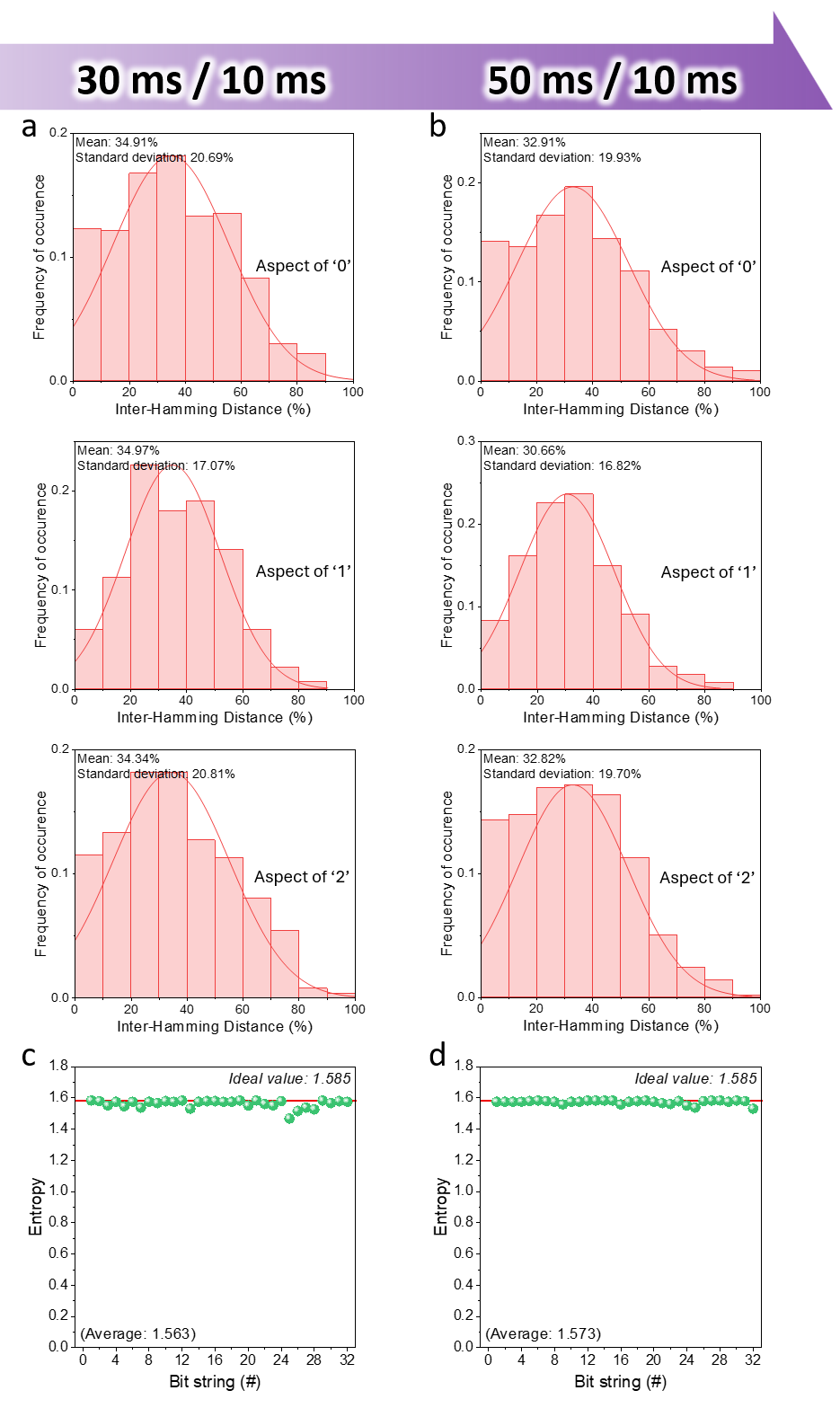


**Figure S53.** Inter-HD and entropy analysis of the generated ternary random numbers under varying optical pulse conditions. Inter-HD distributions obtained at optical pulse conditions of (a) 30 ms/10 ms, and (b) 50 ms/10 ms. (c-d) Corresponding entropy values extracted under each illumination condition.


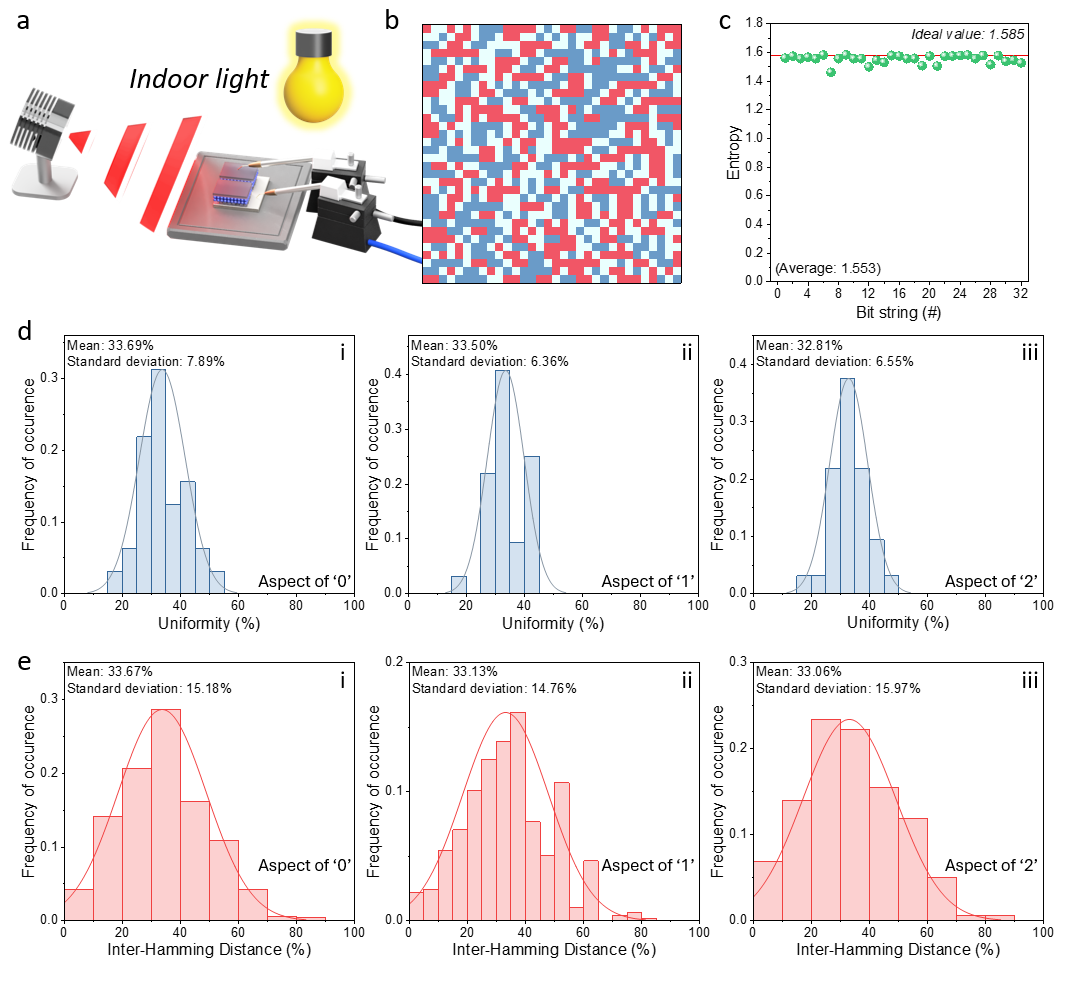


**Figure S54.** Evaluation of PS-TRNG operation under ambient indoor light. (a) Measurement-environment schematic. (b) 32 × 32 ternary mapping showing uniformly distributed random outputs obtained under ambient light. (c) Entropy analysis confirming a value close to the theoretical ideal. (d) Uniformity evaluation for values ‘0’, ‘1’, and ‘2’, each exhibiting occurrence ratios close to the theoretical ideal of 33.33 %. (e) Inter-HD near 33.33 %, confirming stable and unbiased randomness under indoor lighting.


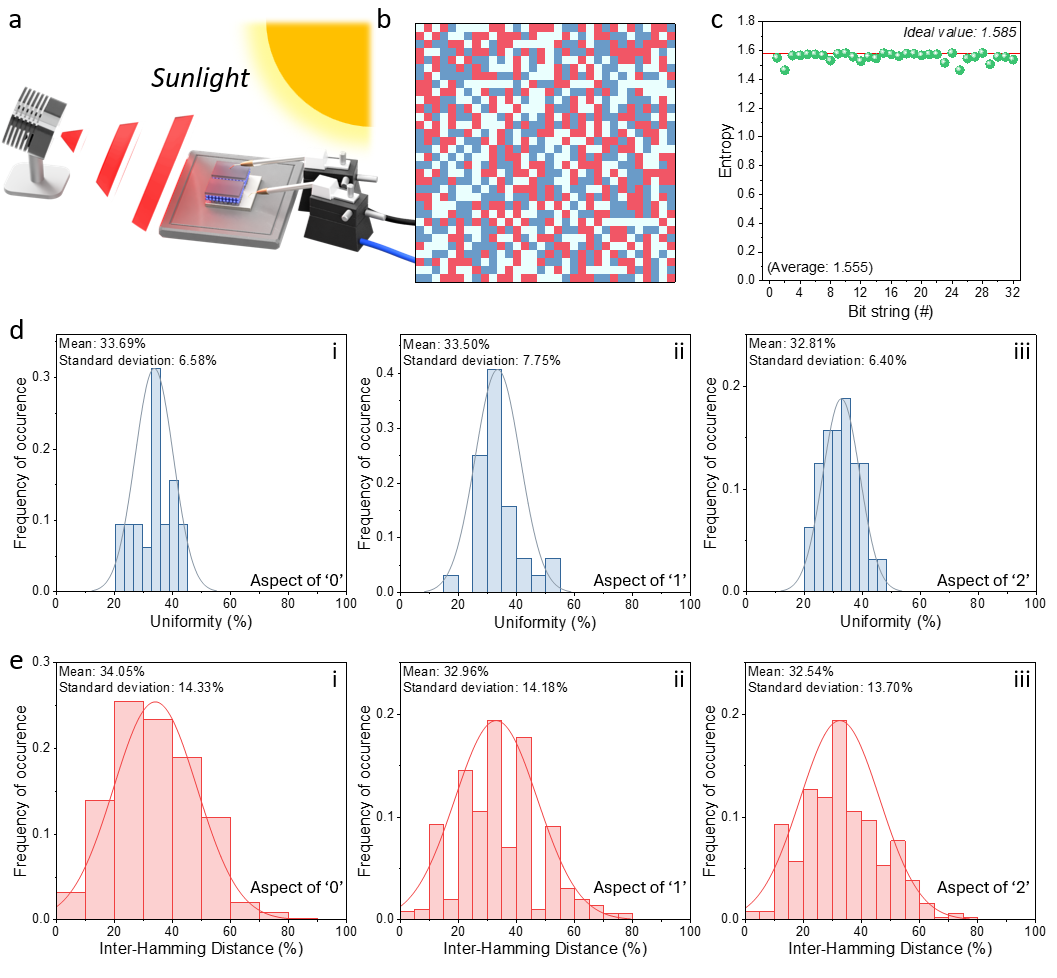


**Figure S55.** Evaluation of PS-TRNG operation under natural sunlight. (a) Measurement-environment schematic. (b) 32 × 32 ternary mapping showing uniformly distributed random outputs under solar illumination. (c) Entropy analysis close to the theoretical ideal. (d) Uniformity for ‘0’, ‘1’, and ‘2’, each near 33.33 %. (e) Inter-HD near 33.33 %, confirming stable and unbiased randomness under natural sunlight.


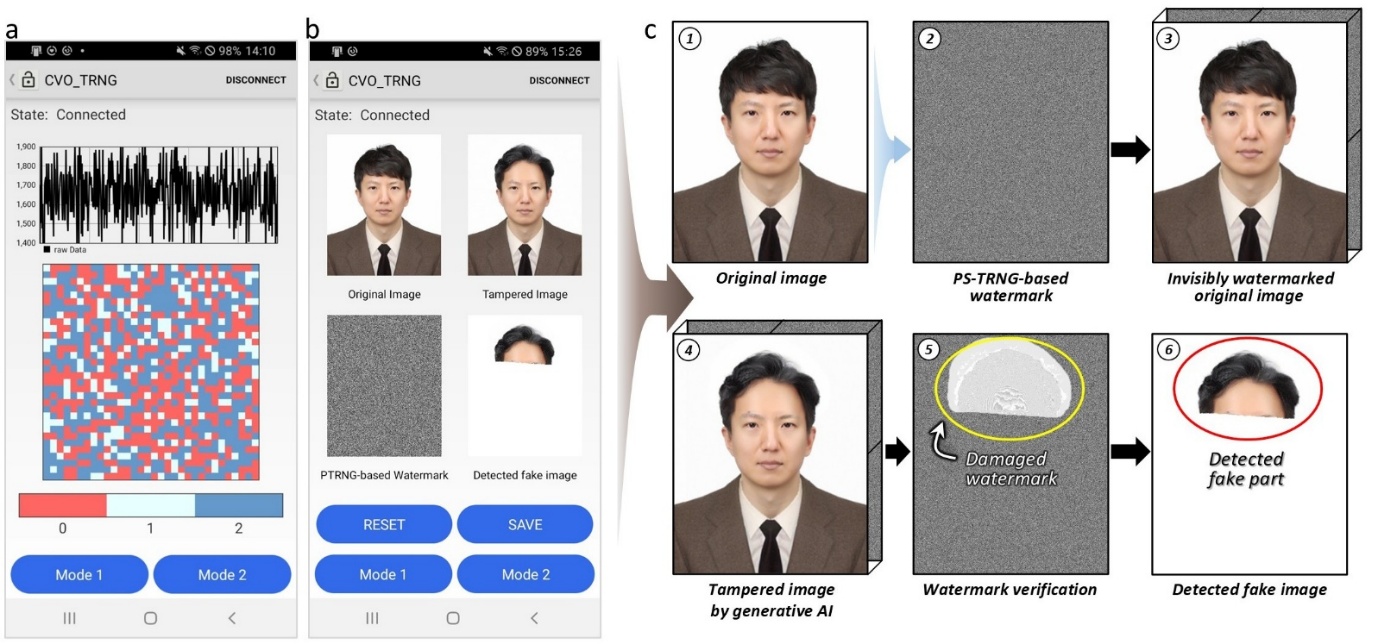


**Figure S56.** Pixel tampering diagnosis system of image encryption and verification using PS-TRNG. (a, b) Real-time fake image detection platform utilizing ternary random numbers generated by the PS-TRNG and interfaced with a smartphone. (c) Sequential workflow of the pixel-level tampering diagnosis process, shown with a portrait of the author, Young-Joon Kim. Images of individuals are used with consent.


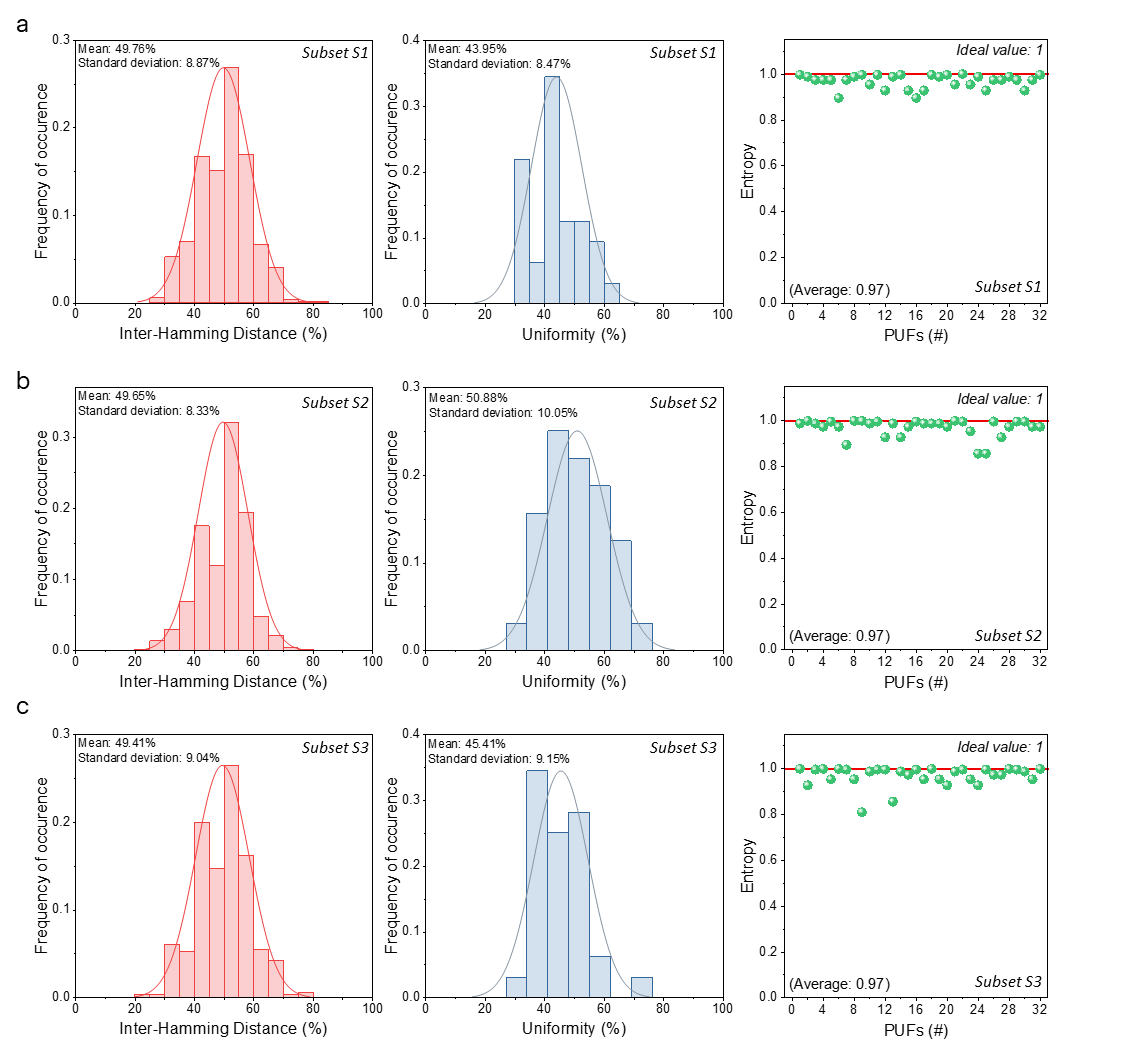


**Figure S57.** Plots of inter-HD, uniformity, and entropy for three binary subsets generated from different ternary pairs: (a) S1, (b) S2, and (c) S3.

**
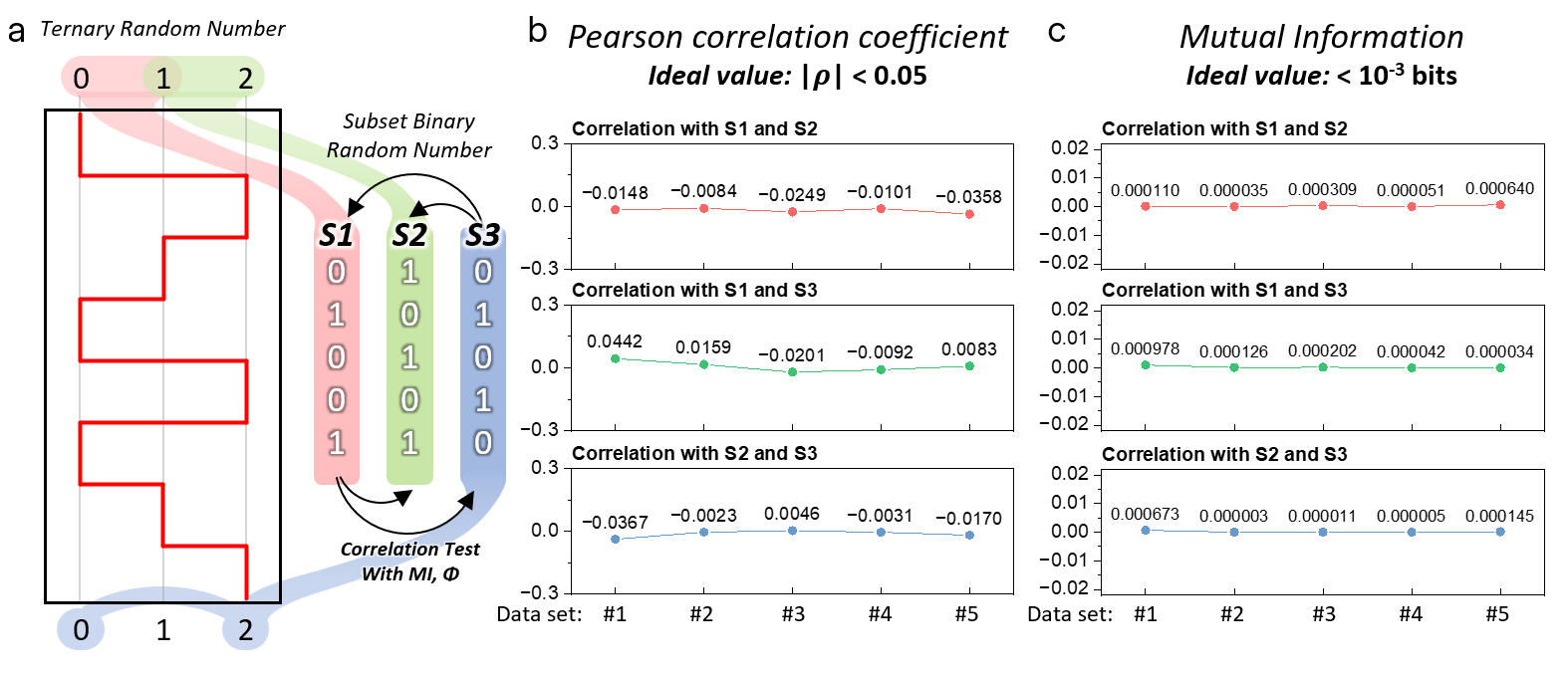
**

**Figure S58.** Correlation analysis of binary subsets derived from the ternary random sequence. (a) Schematic illustrating the extraction of three binary subsets from the ternary random numbers. (b) Pearson correlation coefficient values computed among the three subsets. (c) Mutual information results evaluating nonlinear dependence among the subsets.

**
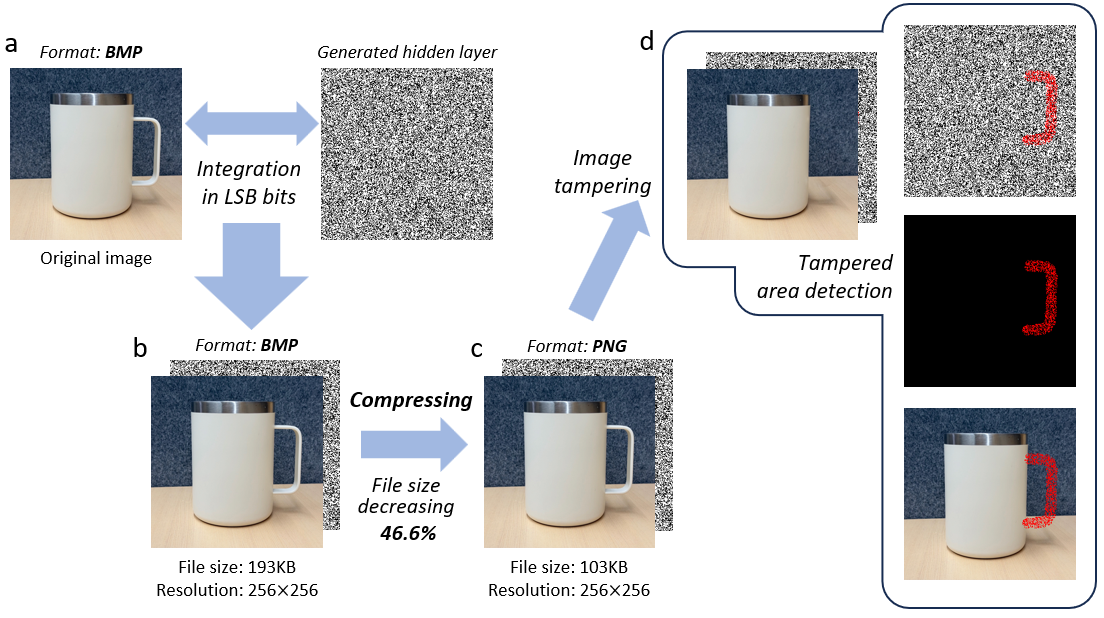
**

**Figure S59.** Robustness test of the PTD system under image format conversion and compression. (a) Original mug image and schematic of hidden layer embedding in the LSB domain. (b) Image with the embedded hidden layer stored in BMP format. (c) Image converted to PNG format with compression. (d) PTD system output showing successful detection of the modified mug handle after format change and compression.


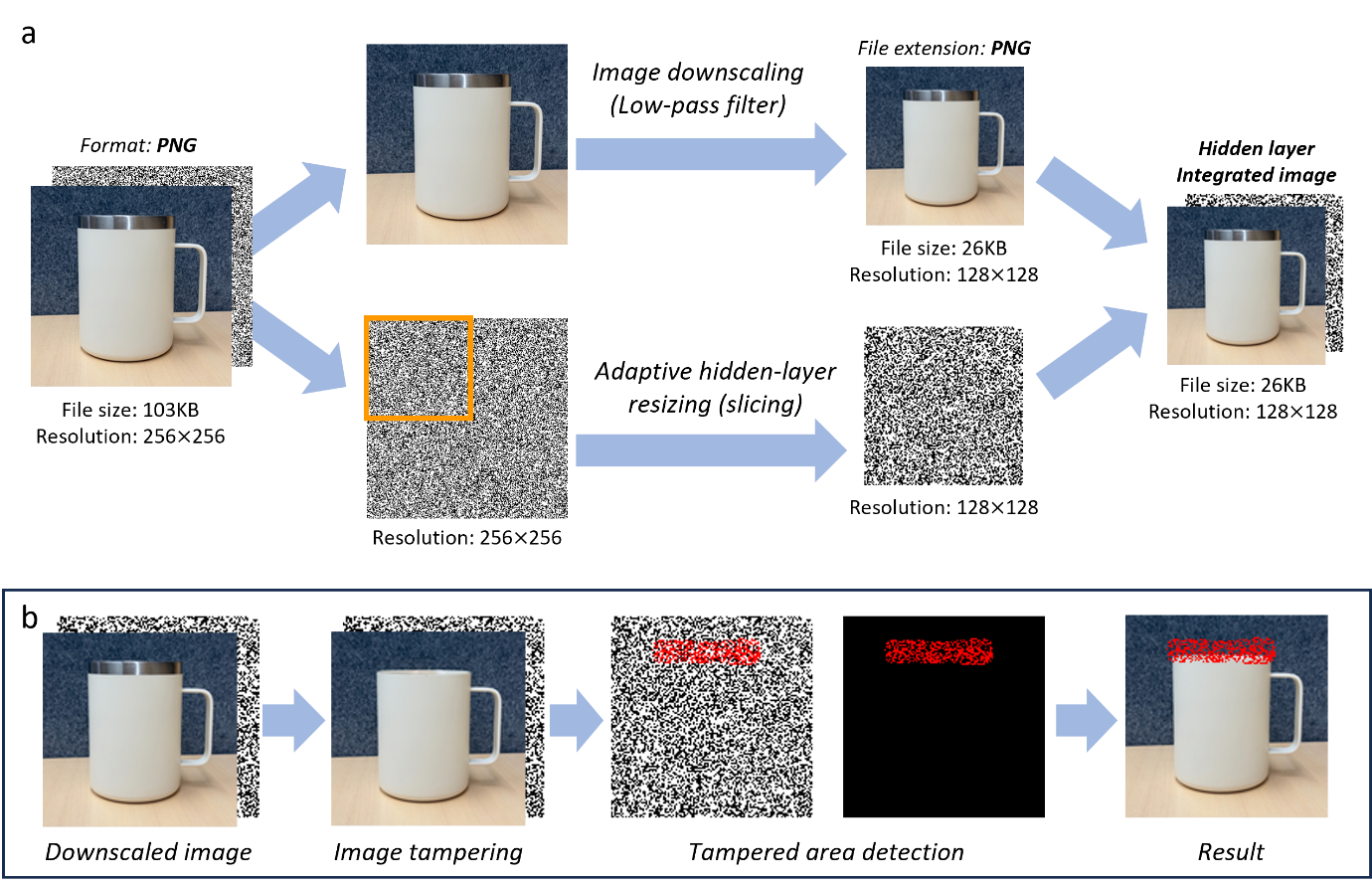


**Figure S60.** Robustness of the PTD system under image resizing and filtering. (a) Original mug image and schematic illustrating the extraction of the hidden layer stored in the LSB domain and its reinsertion through adaptive hidden-layer resizing. (b) Operation of the PTD system on the resized image, demonstrating successful detection even after the image has been resampled through a low-pass filtering process.


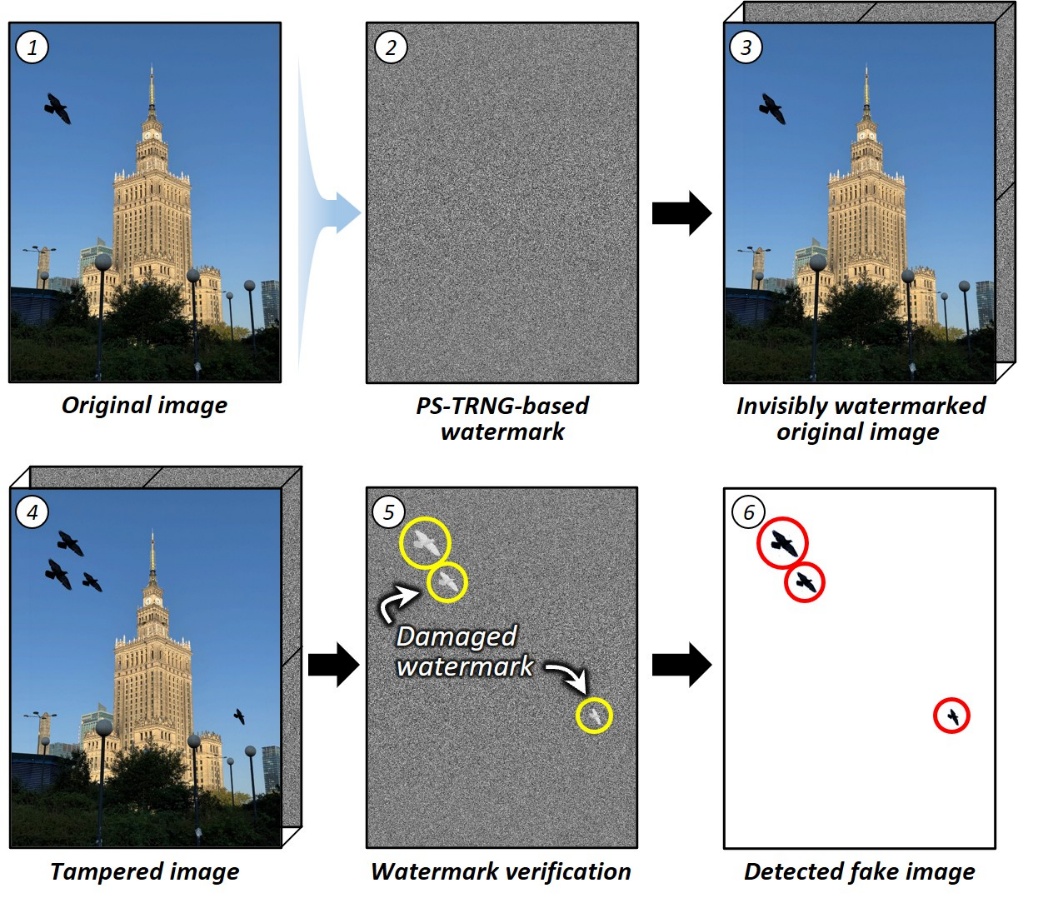


**Figure S61.** Demonstration of PS-TRNG-based watermark generation and image-tamper detection, showing the sequential workflow of the pixel-level tampering diagnosis process.


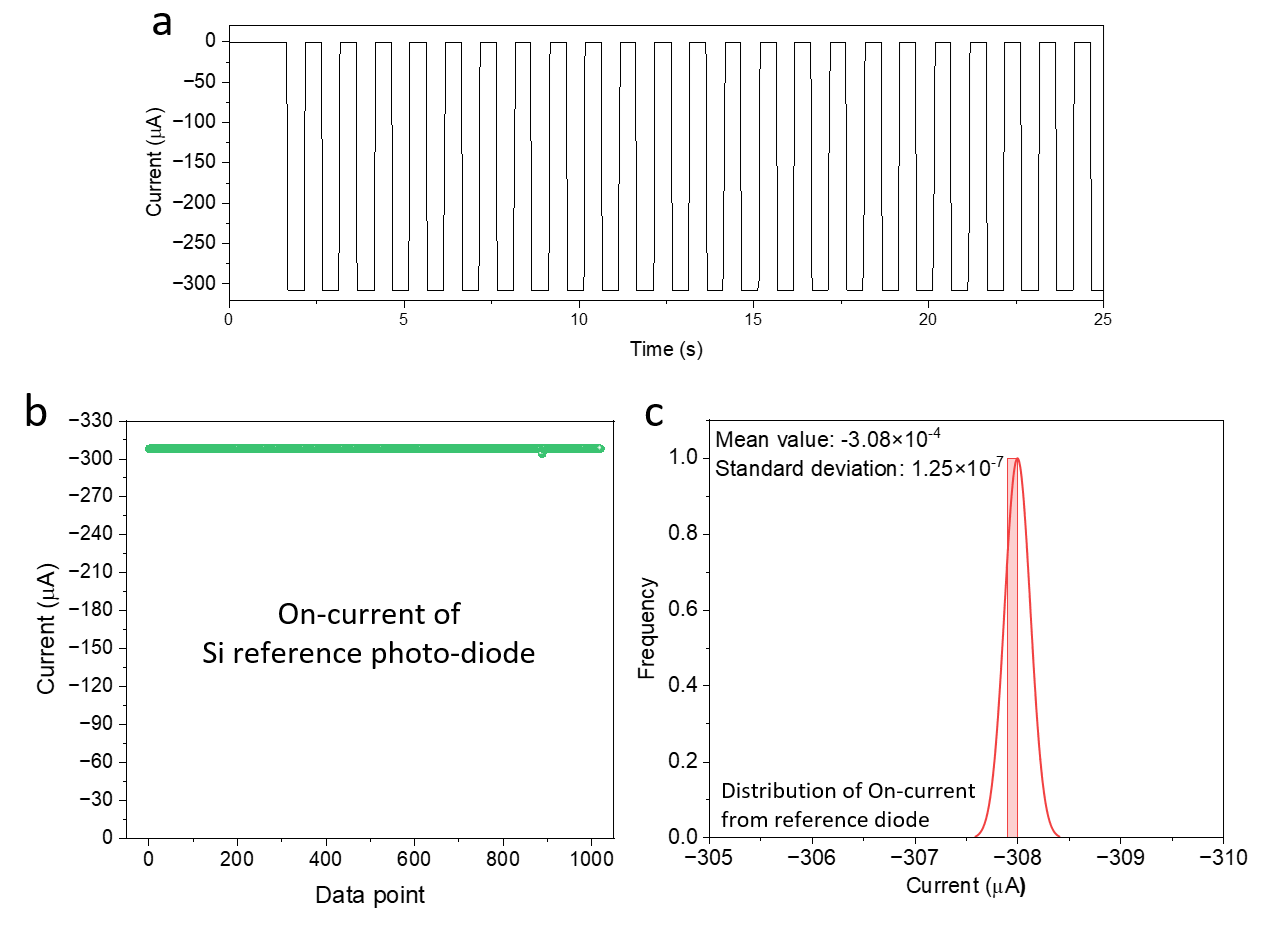


**Figure S62.** Verification of the optical source stability using a reference Si photodiode. (a) Time–current profile showing periodic pulse signals without spiking behavior. (b, c) Photocurrent distribution and intensity uniformity under illumination, maintaining an average value of $-$308 μA with a standard deviation of 1.25 $\times$ 10^-7^ A, confirming the optical source’s stable output.

**Table S1.** NIST test suite results of PS-TRNG (Aspect of 0 and 1).

| **No.** | **Index^*^** | **Statical Test** | **P-value** | **Pass** |
| --- | --- | --- | --- | --- |
| 1 | A1 | Cumulative sums test (mean value) | 0.112 | Passed |
| 2 | A2 | Random excursion test (mean value) | 0.126 | Passed |
| 3 | A3 | Test for the longest run of ones in a block | 0.307 | Passed |
| 4 | A4 | Serial test (mean value) | 0.457 | Passed |
| 5 | A5 | Binary matrix rank test | 0.566 | Passed |
| 6 | B1 | Frequency test within a block | 0.113 | Passed |
| 7 | B2 | Discrete Fourier transform | 0.246 | Passed |
| 8 | B3 | Linear complexity | 0.331 | Passed |
| 9 | B4 | Random excursion variation test (mean value) | 0.428 | Passed |
| 10 | B5 | Overlapping template matching test | 0.873 | Passed |
| 11 | C1 | Frequency (mono bit) test | 0.120 | Passed |
| 12 | C2 | Maurer's "Universal Statistical" test | 0.216 | Passed |
| 13 | C3 | Non-overlapping template matching test | 0.251 | Passed |
| 14 | C4 | Approximate entropy test | 0.466 | Passed |
| 15 | C5 | Runs test | 0.682 | Passed |

**^*^**Each index entry corresponds to a label in #1 of Figure 2g and Figure S18.

**Table S2.** NIST test suite results of PS-TRNG (Aspect of 1 and 2).

| **No.** | **Index^*^** | **Statical Test** | **P-value** | **Pass** |
| --- | --- | --- | --- | --- |
| 1 | A1 | Cumulative sums test (mean value) | 0.208 | Passed |
| 2 | A2 | Random excursion test (mean value) | 0.584 | Passed |
| 3 | A3 | Test for the longest run of ones in a block | 0.647 | Passed |
| 4 | A4 | Serial test (mean value) | 0.797 | Passed |
| 5 | A5 | Binary matrix rank test | 0.761 | Passed |
| 6 | B1 | Frequency test within a block | 0.112 | Passed |
| 7 | B2 | Discrete Fourier transform | 0.343 | Passed |
| 8 | B3 | Linear complexity | 0.471 | Passed |
| 9 | B4 | Random excursion variation test (mean value) | 0.525 | Passed |
| 10 | B5 | Overlapping template matching test | 0.251 | Passed |
| 11 | C1 | Frequency (mono bit) test | 0.423 | Passed |
| 12 | C2 | Maurer's "Universal Statistical" test | 0.245 | Passed |
| 13 | C3 | Non-overlapping template matching test | 0.216 | Passed |
| 14 | C4 | Approximate entropy test | 0.325 | Passed |
| 15 | C5 | Runs test | 0.379 | Passed |

**^*^**Each index entry corresponds to a label in #2 of Figrue 2g and Figure S18.

**Table S3.** NIST test suite results of PS-TRNG (Aspect of 0 and 2).

| **No.** | **Index^*^** | **Statical Test** | **P-value** | **Pass** |
| --- | --- | --- | --- | --- |
| 1 | A1 | Cumulative sums test (mean value) | 0.678 | Passed |
| 2 | A2 | Random excursion test (mean value) | 0.451 | Passed |
| 3 | A3 | Test for the longest run of ones in a block | 0.754 | Passed |
| 4 | A4 | Serial test (mean value) | 0.267 | Passed |
| 5 | A5 | Binary matrix rank test | 0.442 | Passed |
| 6 | B1 | Frequency test within a block | 0.193 | Passed |
| 7 | B2 | Discrete Fourier transform | 0.457 | Passed |
| 8 | B3 | Linear complexity | 0.352 | Passed |
| 9 | B4 | Random excursion variation test (mean value) | 0.452 | Passed |
| 10 | B5 | Overlapping template matching test | 0.386 | Passed |
| 11 | C1 | Frequency (mono bit) test | 0.137 | Passed |
| 12 | C2 | Maurer's "Universal Statistical" test | 0.150 | Passed |
| 13 | C3 | Non-overlapping template matching test | 0.235 | Passed |
| 14 | C4 | Approximate entropy test | 0.561 | Passed |
| 15 | C5 | Runs test | 0.269 | Passed |

**^*^**Each index entry corresponds to a label in #3 of Figrue 2g and Figure S18.

**Table S4.** NIST test suite results of binary PRNG.

| **No.** | **Index^*^** | **Statical Test** | **P-value** | **Pass** |
| --- | --- | --- | --- | --- |
| 1 | A1 | Cumulative sums test (mean value) | 7.45E-6 | Fail |
| 2 | A2 | Random excursion test (mean value) | 0.56 | Passed |
| 3 | A3 | Test for the longest run of ones in a block | 0.04 | Passed |
| 4 | A4 | Serial test (mean value) | 0.73 | Passed |
| 5 | A5 | Binary matrix rank test | 0.99 | Passed |
| 6 | B1 | Frequency test within a block | 0.17 | Passed |
| 7 | B2 | Discrete Fourier transform | 0.33 | Passed |
| 8 | B3 | Linear complexity | 0.78 | Passed |
| 9 | B4 | Random excursion variation test (mean value) | 0.41 | Passed |
| 10 | B5 | Overlapping template matching test | 0.59E-3 | Fail |
| 11 | C1 | Frequency (mono bit) test | 6.8E-9 | Fail |
| 12 | C2 | Maurer's "Universal Statistical" test | 0.95 | Passed |
| 13 | C3 | Non-overlapping template matching test | 0.98 | Passed |
| 14 | C4 | Approximate entropy test | 0.21 | Passed |
| 15 | C5 | Runs test | 0 | Fail |

**^*^**Each index entry corresponds to a label in Figure 2h and 2i.

**Table S5.** Readout circuit specifications for the PS-TRNG system.

| Component | Parameter | Compression type |
| --- | --- | --- |
| ADC | Resolution | 8-bit |
|  | Sampling rate | 100 kHz |
| TIA | Bandwidth | 33.9 kHz |
|  | Amplifier model | AD8605-class |
|  | Voltage noise density | 8–12 nV/√Hz, f = 1 kHz |
|  |  | 6.5 nV/√Hz, f = 10 kHz |
|  | Current noise density | 0.01 pA/√Hz, f = 1 kHz |
|  | Peak-to-peak noise | 2.3–3.5 μV p-p (0.1–10 Hz) |
| System | Nyquist compliance | Yes |
|  | Contribution to entropy | Negligible |

**Table S6.** Evaluation of hidden-layer robustness under format conversion and filtering.

|  | Image format | Compression type | LSB domain | Hidden layer retention (%) | Detection reliability (%) | Image |
| --- | --- | --- | --- | --- | --- | --- |
| 1 | BMP | None | Present | 100 | 100 | 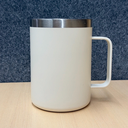 |
| 2 | PNG | Lossless | Present | 100 | 100 | 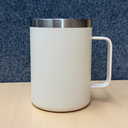 |
| 3 | TIFF | Lossless | Present | 100 | 100 | 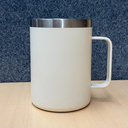 |
| 4 | WEBP | Lossless | Present | 100 | 100 | 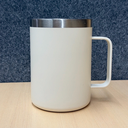 |
| 5 | GIF | Palette-based (Lossy) | Partial | 49.44 | 0 | 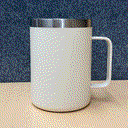 |
| 6 | JPEG | Lossy | Absent | 49.38 | 0 | 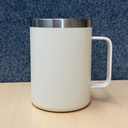 |

**Table S7.** Comparison of optical TRNGs covering optical conditions, random number generation capability, power consumption, and stability.

| **Entropy source** | **Wavelength [nm]** | **Optical type** | **Bit type** | **Bit rate [bit/s]** | **Parallel-channel bit-rate enhancement** | **Power consumption** | **Long-term stability [day]** | **Applications** | **[Ref]** |
| --- | --- | --- | --- | --- | --- | --- | --- | --- | --- |
| Thermally induced atomic rearrangement | 633 | He–Ne laser | Binary | ≈ 1.7 × 10^4^ | - | 5 mW | 1 hour | - | [6] |
| Structural frustration of nematic liquid crystals | - | Electric field driven | Binary | ≈ 6 | - | 10 Vpp, 1 kHz | - | - | [7] |
| Broadband chaotic comb | Pump: ~1550, Comb span: 1300–1740 | Micro-resonator | Binary | 126 × 10^12^ | 450 | 27.15 dBm, 0.52 W | - | - | [8] |
| Rayleigh scattering and modulation instability | Pump: 1480, Emission: 1572 | Raman fiber laser | Binary | 5.4 × 10^11^ | - | 5.6 W | - | - | [9] |
| Pulse-to-pulse intensity fluctuation | Pump: 1556, SC: 1300–1700 | Pulse (10 GHz, MLL) | Binary | 4 × 10^10^ | 4 | ~0.5 mW | - | - | [10] |
| Trap-detrap | 450 | Continuous illumination | Binary | 1 × 10^4^ | - | 7 mW | - | Image encryption | [11] |
| Intensity fluctuation, band competition | Pump: 440–540, Emission: 590, 630 | Pulse (10 Hz, OPO laser) | Binary | 10 | - | 5.5 mJ·cm⁻² per pulse | - | - | [12] |
| Dual-band competition | 200–1000 | Arc-discharge | Binary | 2 × 10^3^ | - | 88.5–330  μW·cm⁻² | 180 | X-ray image generation | [13] |
| Trap-detrap | 660 | Pulse (50 Hz) | Binary, Ternary | 11.89 (156.15 trit/s) | 8 | 0.53 mW·cm⁻² | 460 | Image tamper-prevention/authentication | This work |

**Supporting Movie**

**Movie S1.** Demonstration of real-time ternary random number generation followed by image encryption and decryption using the PS-TRNG. Images of individuals are used with consent.

**Movie S2****.** Demonstration of the PTD system operation using generated random numbers. Images of individuals are used with consent.

**REFERENCE**

[1] M.-w. Kim, B. Joshi, H. Yoon, T. Y. Ohm, K. Kim, S. S. Al-Deyab, S. S. Yoon, Electrosprayed copper hexaoxodivanadate (CuV2O6) and pyrovanadate (Cu2V2O7) photoanodes for efficient solar water splitting, Journal of Alloys and Compounds 2017, 708, 444.

[2] J. Park, T. Park, Y. J. Kim, H. Yoo, Light-induced, room-temperature hydrogen gas detection based on SnO2 quantum Dots/p-Si, Applied Surface Science 2024, 670, 160693.

[3] A. Song, S. Liu, Q. Wang, D. Gao, J. Hu, Comprehensive evaluation of copper vanadate (α-CuV2O6) for use as a photoanode material for photoelectrochemical water splitting, Journal of Environmental Chemical Engineering 2023, 11, 109892.

[4] M. Yang, Z. Ye, H. Pan, M. Farhat, A. E. Cetin, P.-Y. Chen, Electromagnetically unclonable functions generated by non-Hermitian absorber-emitter, Science Advances 2023, 9, eadg7481.

[5] Y. C. Chien, H. Xiang, J. Wang, Y. Shi, X. Fong, K. W. Ang, Attack Resilient True Random Number Generators Using Ferroelectric‐Enhanced Stochasticity in 2D Transistor, Small 2023, 19, 2302842.

[6] Y. Yang, R. Hui, X. Lu, L. Tang, Z. Gan, M. Li, J. Zhang, K. Chen, F. Chen, W. Huang, Optical True Random Number Generator Based on Reconfigurable Ge2Sb2Te5 PUFs, ACS Photonics 2024, 11, 4691.

[7] J. Y. Lee, J. H. Lee, J. S. Kim, J. S. Yu, J. H. Kim, Robust and Accessible True Random Number Generator Exploiting the Structural Frustration of Nematic Liquid Crystals, Advanced Optical Materials 2023, 11, 2301125.

[8] L. Zhao, W. Xie, M. Wu, Y. Xiao, Z. Shen, J. Deng, H. Li, Z. Wu, J. Yang, W. Wei, 126 Tbits/s Massive Parallel Physical Random Bits Generator with Broadband Chaos of Integrated AlGaAs Micro‐Resonator, Laser & Photonics Reviews 2025, e00671.

[9] F. Monet, J.-S. Boisvert, R. Kashyap, A simple high-speed random number generator with minimal post-processing using a random Raman fiber laser, Scientific reports 2021, 11, 13182.

[10] P. Li, K. Li, X. Guo, Y. Guo, Y. Liu, B. Xu, A. Bogris, K. Alan Shore, Y. Wang, Parallel optical random bit generator, Optics letters 2019, 44, 2446.

[11] D. Ahn, M. Lee, W. Kim, Y. K. Lee, J. Y. Lee, G. Y. Jung, H. Choi, Y. Yoon, H. S. Song, H. Lee, Stochastic Photoresponse‐Driven Perovskite TRNGs for Secure Encryption Systems, Advanced Science 2025, 12, 2412139.

[12] L. Sznitko, T. Chtouki, B. Sahraoui, J. Mysliwiec, Bichromatic laser dye as a photonic random number generator, ACS Photonics 2021, 8, 1630.

[13] T. Park, J. Seo, N. Kim, C. Kim, Y. J. Kim, H. Kim, H. H. Kim, S. Oh, D. C. Kim, D. Son, Rolling the Dice with Light Competition: Introducing a True Random Number Generator Powered by Photo‐Induced Polarity Current, Advanced Materials 2025, 37, 2419579.
